# Synthesis of a Complex Brasilicardin Analogue Utilizing a Cobalt-Catalyzed MHAT-Induced Radical Bicyclization Reaction

**DOI:** 10.1021/acs.orglett.3c01019

**Published:** 2023-05-04

**Authors:** Scott W. Niman, Roberta Buono, David A. Fruman, Christopher D. Vanderwal

**Affiliations:** † Department of Chemistry, University of California, 1102 Natural Sciences II, Irvine, California 92697-2025, United States; ‡ Department of Molecular Biology & Biochemistry, University of California, 3205 McGaugh Hall, Irvine, California 92697-2525, United States; § Department of Pharmaceutical Sciences, University of California, 101 Theory #100, Irvine, California 92617, United States

## Abstract

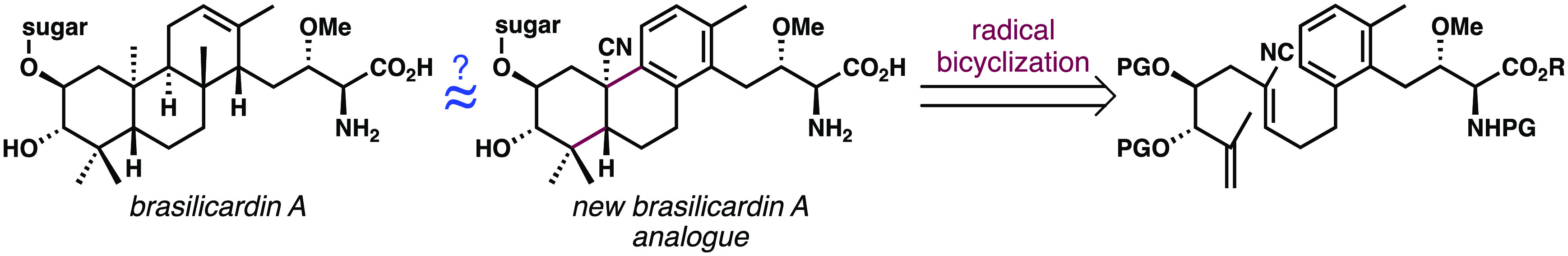

We designed and executed an expedient synthesis of a complex analogue of the potent immunosuppressive natural product brasilicardin A. Our successful synthesis featured application of our recently developed MHAT-initiated radical bicyclization, which delivered the targeted, complex analogue in 17 steps in the longest linear sequence. Unfortunately, this analogue showed no observable immunosuppressive activity, which speaks to the importance of the structural and stereochemical elements of the natural core scaffold.

Polycyclic terpenoid natural products often possess interesting, diverse, and potent biological activities. One example is brasilicardin A (brasA, **1**, Figure 1a) whose unique structure elicits powerful immunosuppressive activity in a mouse mixed-lymphocyte reaction (MLR) assay (IC_50_ = 0.057 μg/mL).^1,2^ Further, brasA appears to possess a novel mode of action that differs from clinically used immunosuppressants, and it has been shown to have very low toxicity in mice, with no negative effects even at a dose of 100 mg/kg.^2^ The stereochemically unusual tricyclic “core” structure, with its B ring confined to a boat conformation, renders the application of biomimetic, cationic polyene cyclization for its synthesis challenging.^3^ The combination of potentially important activity and unusual structure garnered brasA significant interest from synthetic chemists. Two total syntheses have been reported; however, they suffer from lengthy sequences (>30-step longest linear sequence) arising from a difficulty to efficiently install the boat-containing ring system.^4,5^ Inspiring work by the groups of Stegmann, Koch, Méndez, and Gross has demonstrated that total biosynthesis of the brasilicardin A aglycone is now feasible on gram scale.^6^ Despite these key advances, access to unnatural analogues of this secondary metabolite remains an unsolved problem that impedes more substantial structure–activity relationship (SAR) efforts. Such studies could identify simplified structures that maintain high potency and that might serve as leads for immunosuppressive drug discovery efforts.

**Figure 1:**
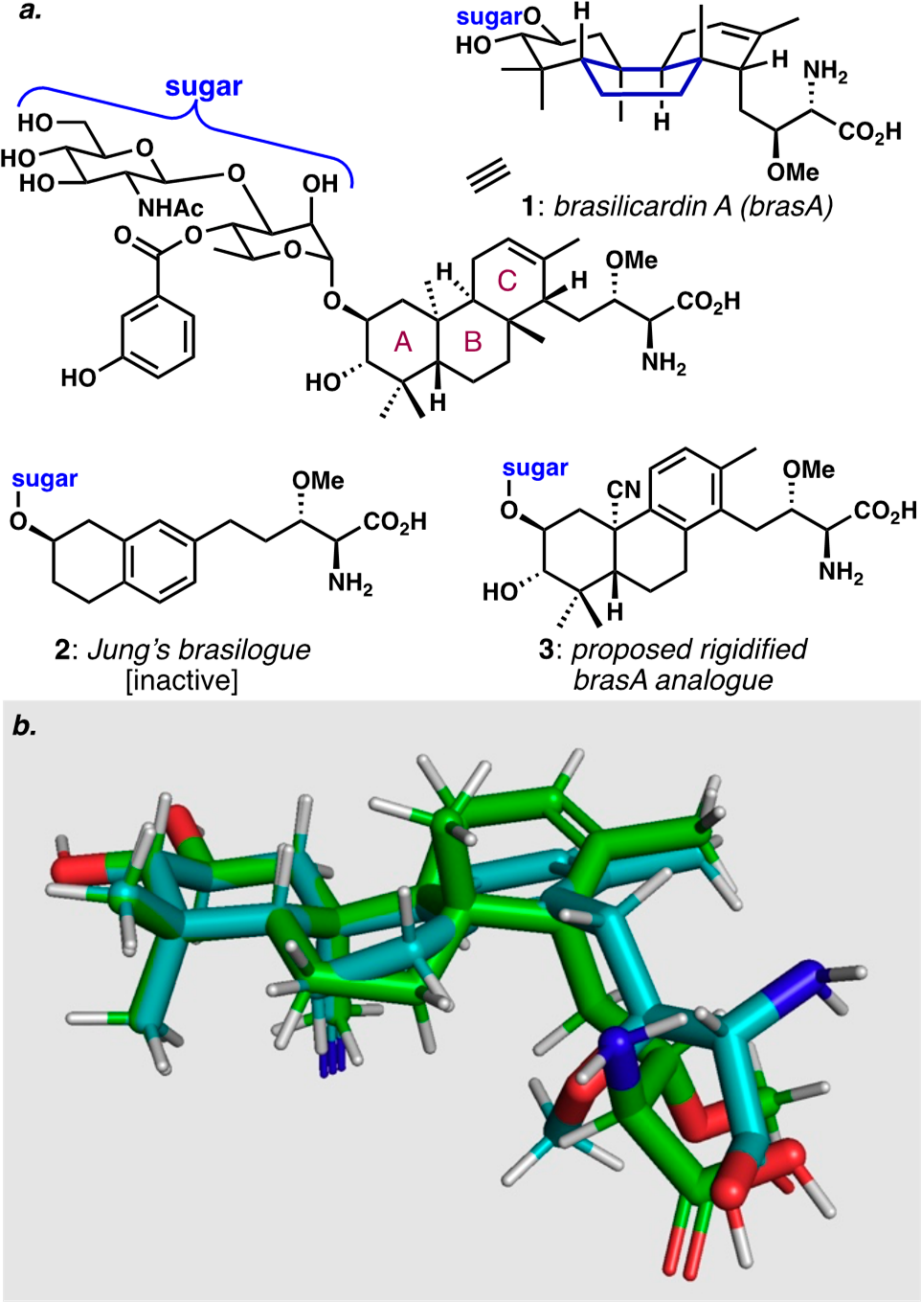
(a) Structures of brasilicardin A (**1**) and two analogues. (b) Overlay of brasilicardin A aglycone (green) and aglycone of proposed analogue **3** (blue) from PyMOL, minimized with ωB97X-D/6-31G(d).

The minimal SAR data available for **1** have implied that both the glycosidic region and the amino acid are critical for potent activity.^7^ The Jung lab probed the importance of the central ring scaffold via the synthesis and evaluation of the unnatural analogue **2** that they named “brasilogue”.^8,9^ This compound evolved from their approximation of the requisite tether distance between the glycosyl unit and the amino acid. However, this bicyclic scaffold also omitted many other features including: multiple space-filling, hydrophobic methyl groups, one hydroxyl group, the entirety of the C ring, and a considerable amount of rigidity between the two critical linked chemotypes. Unfortunately, **2** showed none of the natural product’s immunosuppressive activity. The goal of our work described herein was to see if a readily accessible, rigid yet unnatural, core scaffold would suffice to display the polar groups at each end of the molecule in such a way as to recapitulate the activity of the naturally occurring immunosuppressive agent.

Recently, our group developed a cobalt-catalyzed, MHAT-induced radical bicyclization method that efficiently produces highly oxidized diterpene-like tricyclic products (**4**).^10−12^ We recognized that this protocol might rapidly provide a more rigid but still-simplified analogue of brasA, such as **3**. Indeed, an overlay of the calculated conformational minima of the aglycones of brasA and **3** (Figure 1b) suggested a reasonable approximation in terms of the potential display of both the disaccharide and amino acid motifs. We considered that the synthesis and study of the proposed analogue would be meaningful because the closer approximation of the core structure of **3** to that of brasA (compared to Jung’s analogue **2**(9)) could help to discern whether the unusual natural core structure was truly critical or if other rigid polycycles that appropriately displayed the polar groups would suffice. Further, if active, the significantly reduced synthetic cost could potentially enable further SAR studies of brasA analogues.

We envisioned preparing **3** via late-stage chemoselective glycosylation of unprotected diol **6** (Scheme 1). This strategy has been realized by Yoshimura and co-workers^5^ in their brasA total synthesis, where excellent regio- and anomeric selectivity were observed employing glycosyl fluoride donor of type **5** (X = F). The requisite tricyclic substrate could be synthesized using our group’s cobalt MHAT bicyclization methodology with a suitably protected diene precursor **7**. We planned to prepare the diene by convergent Horner–Wadsworth–Emmons (HWE) alkenylation, which required aldehyde **8** and cyanophosphonate **9**. We recently reported a synthesis of the enantiomer of **9**, which was employed in our synthesis and structural revision of plebeianiol A.^12^ We were confident that this approach would allow for rapid construction of the unnatural core of **3**, further testing the limits of the bicyclization method with the presence of the amino acid side chain, and provide us with sufficient quantities of a complex brasA analogue for biochemical evaluation of immunosuppressive activity.

**Scheme 1:**
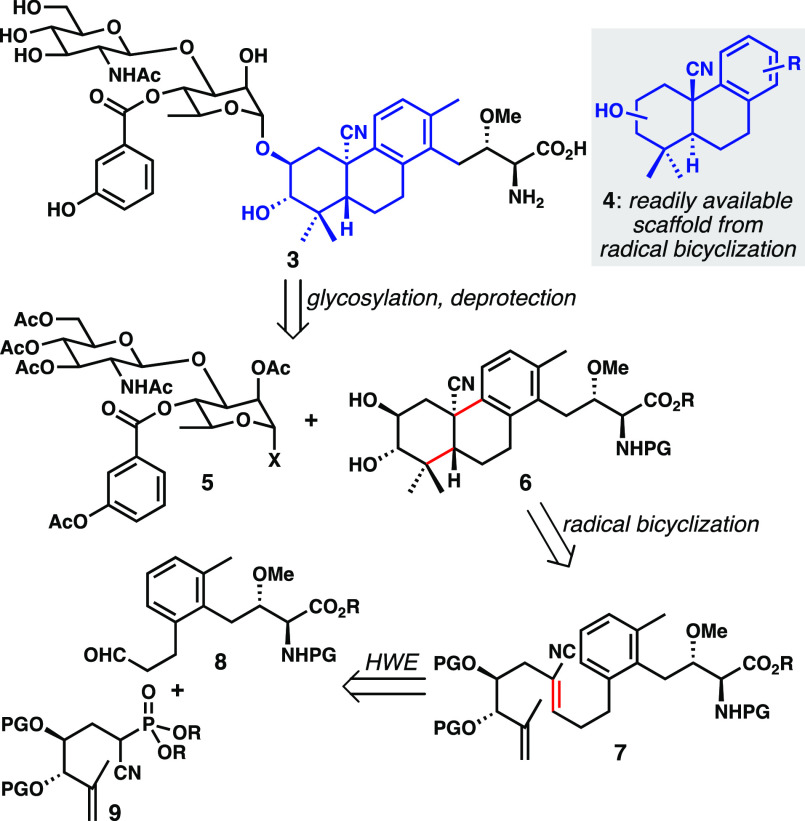
Retrosynthetic Analysis of brasA Analogue **3** (HWE = Horner–Wadsworth–Emmons Alkenylation)

Although we originally developed a successful synthesis based on a key diastereoselective aldol addition (Scheme 2),^13^ we were plagued with serious issues of material throughput derived from a problematic methylation reaction of a secondary alcohol. We therefore elected to build the amino acid motif via Sharpless asymmetric dihydroxylation (SAD) in a fashion related to the work of Yoshimura, Tanino, and co-workers.^5^ Key to both of these disconnections is the intermediate trisubstituted arene **10**, which has handles for both the amino acid installation as well as a late-stage cross coupling to incorporate the propanal substituent.

**Scheme 2:**
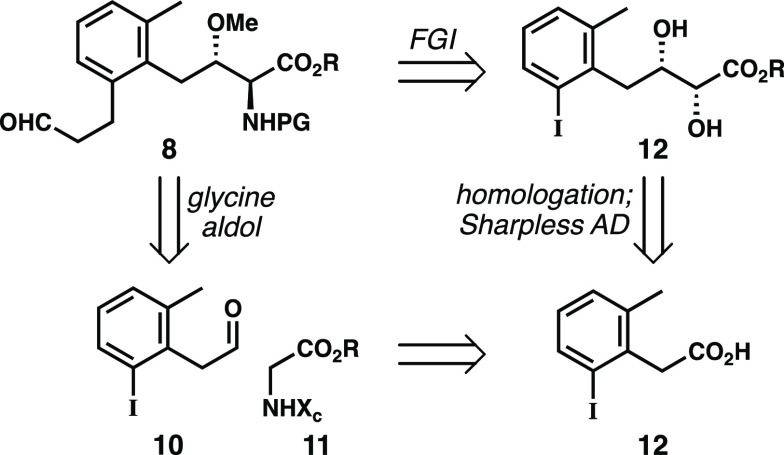
Two Possible Plans for the Synthesis of Aldehyde **8**

Our synthesis began with Yu’s selective iodination of commercially available *o*-tolylacetic acid **13** employing palladium catalysis (Scheme 3).^14^ After borane reduction of the crude iodo-acid, we obtained alcohol **14** on multigram scale and in good yield over two steps. Oxidation of **14** with the Dess–Martin periodinane followed by Wittig alkenylation of the unstable aldehyde in the same pot afforded acrylate **15**.

**Scheme 3:**
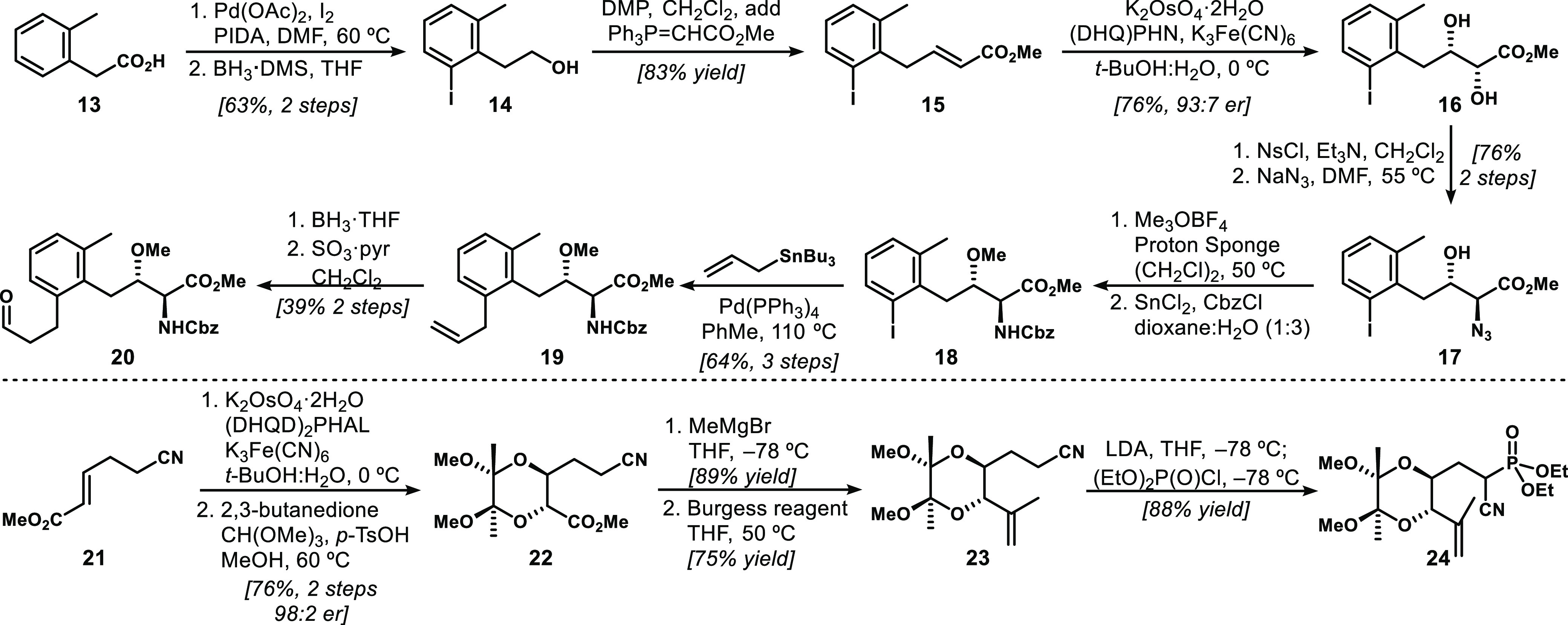
Synthesis of Coupling Fragments **20** and **24**

Sharpless dihydroxylation of **15** using standard AD-mix conditions provided poor levels of enantioselectivity (∼80:20 er). While β-benzyl acrylates such as **15** are a poorly developed class of substrates for SAD chemistry, there are examples of such reactants bearing unfunctionalized arenes that proceed with synthetically useful levels of selectivity.^15^ We hypothesized that, in our system, the ortho substituents in **15** might be the source of the problem, since there are no examples of this type reported in the literature. After extensive screening of common ligands,^16^ we eventually found that one of Sharpless’s early, monomeric ligands, (DHQ)PHN, generated the desired diol **16** with suitable levels of enantioselectivity (93:7 er).^17^

We next shifted our efforts toward installing the *anti*-amino alcohol functionality. To accomplish this goal, we nosylated the α-hydroxyl moiety and displaced it with sodium azide following a protocol reported by Sharpless.^18^ Importantly, this reaction proceeded in excellent yield producing ample quantities of azide **17**. At this stage, we methylated the secondary alcohol with Meerwein’s salt and then subjected the azide to reduction with SnCl_2_ (chosen to avoid reduction of the aryl iodide) along with *in situ* protection as the benzyl carbamate **18**. While we hoped to incorporate the 3-carbon propanal unit through a Jeffery–Heck reaction with allyl alcohol,^19^ this reaction proceeded in low yield while generating large quantities of the corresponding overoxidized cinnamaldehyde. We never managed to suppress formation of this undesired side product, but ultimately circumvented this issue by performing a three-step sequence of Stille allylation, hydroboration, and Parikh–Doering oxidation to yield the desired aldehyde **20**. Although this sequence proved lengthy, we found it suitable for exploring the later stages of our synthesis because it could produce ample quantities of **20**.

The desired enantiomer of cyanophosphonate **24** was made following our previously reported procedures.^12^ This compound is available in six steps from commercial materials in excellent levels of enantiopurity (98:2 er) using (DHQ)_2_PHAL as a chiral ligand (Scheme 3). While we previously observed good selectivity (97:3 er) with (DHQD)_2_PHAL for our work toward plebeianiol A, initially only modest selectivity (90:10 er) was observed under the same conditions with (DHQ)_2_PHAL in this context. We found, however, that simply tailoring the catalyst and ligand loadings furnished the corresponding diol in high enantiopurity (98:2 er) and on a gram scale.

With access to suitable quantities of aldehyde **20** and cyanophosphonate **24**, we executed the convergent HWE alkenylation reaction (Scheme 4). Employing KHMDS at cryogenic temperatures, we observed efficient and highly stereocontrolled (>20:1 *Z:E*) alkene formation. Upon treatment of this diene (**25**) with our standard cobalt-catalyzed, MHAT-initiated radical bicyclization conditions, we observed high-yielding formation of the desired tetracyclic product **26** with complete diastereocontrol, as expected on the basis of our previous work.^11,12^ This reaction could be performed on a >1.0 mmol scale producing >600 mg of the protected aglycone of our targeted brasA analogue **3**.

**Scheme 4:**
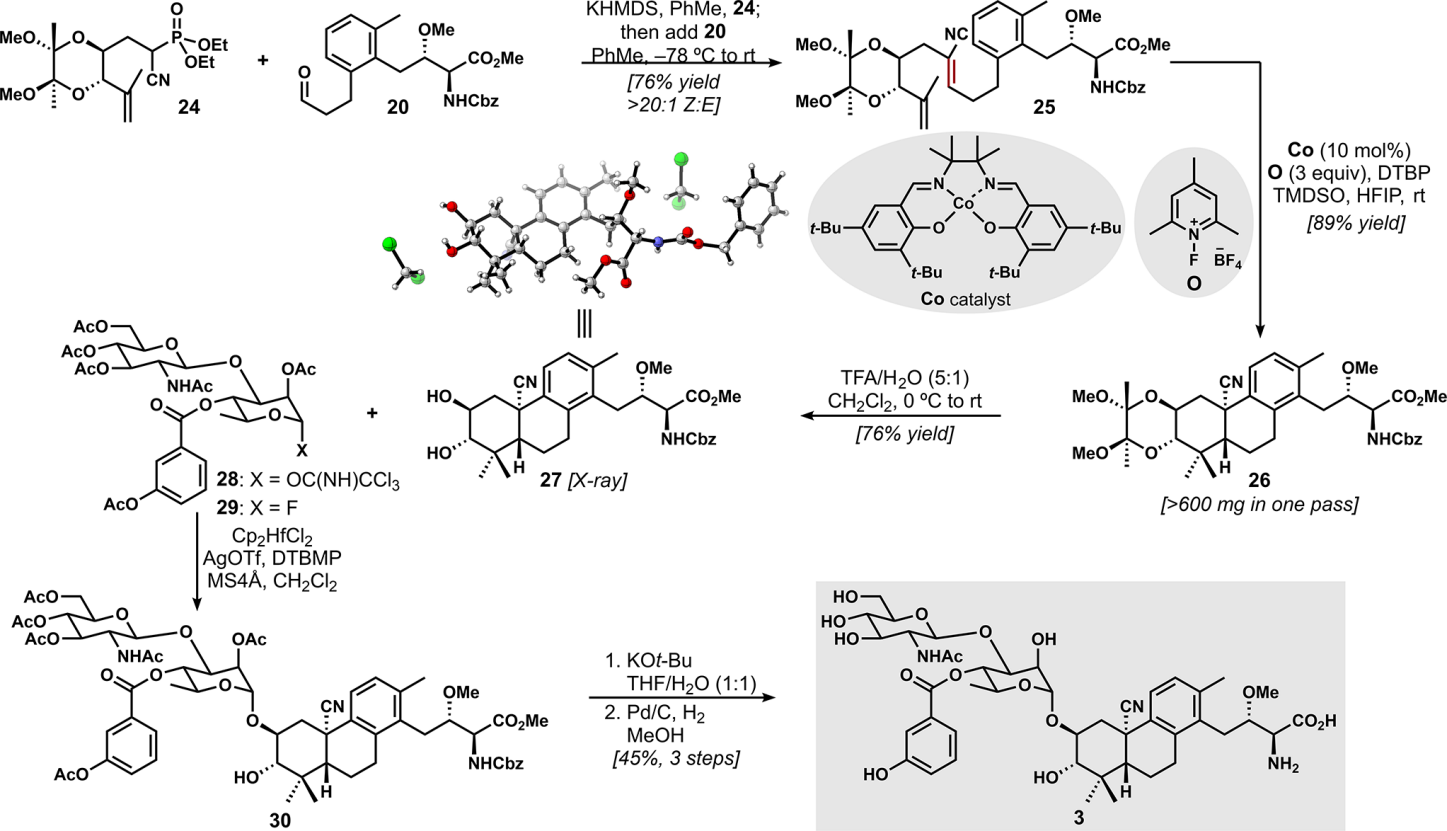
Synthesis of the Targeted brasA Analogue **3** For the X-ray crystallographic structure of **27**, the thermal ellipsoid plot is shown at the 50% probability level.

At this stage, we needed to remove protecting groups and couple the disaccharide unit to our unnatural tricyclic scaffold. Using deprotection conditions reported by Ley,^20^ we cleaved the butanedione acetal with aqueous trifluoroacetic acid to provide diol **27**. Fortunately, this compound proved to be highly crystalline, which allowed confirmation of its configuration via X-ray crystallographic analysis. For the final phase of the synthesis, we investigated the regioselective glycosylation of **27**. Glycosylation using Jung’s trichloroacetimidate **28** with monoprotected brasA-type diols has been effected successfully,^4^ as well as recently with an unprotected diol.^6^ While we prepared **28** and attempted its coupling with **27** under typical Schmidt conditions,^21^ we obtained mixtures of anomeric compounds that were difficult to purify. Instead, on the basis of the procedure of Yoshimura and Tanino,^5^ we executed regioselective glycosylation with fluoride **29**, which provided a crude glycoside with high selectivity for **30**. The glycosylated product was immediately deprotected via basic hydrolysis with KO*t*-Bu and palladium-catalyzed hydrogenolysis to afford **3** in ∼45% yield over three steps. We were able to generate ∼7 mg of analogue **3** via the final 17-step longest linear sequence (LLS), thus providing sufficient material for biological testing.

To test the immunosuppressive potential of analogue **3**, we used two cell-based assays commonly used to study mTOR inhibitors such as rapamycin. mTOR is a kinase that senses signals from both growth factors and nutrients to promote cell growth and proliferation.^22,23^ First, we measured cell cycle distribution in IL-2-dependent CTLL-2 cells treated for 24 h with **3** (30 or 100 nM) or rapamycin (100 nM) (Figure 2). Whereas rapamycin consistently slowed the cell cycle as measured by an increase in the percentage of cells in G1 phase and decrease in S and G2 phases, the brasA analogue did not alter cell cycle distribution compared to untreated control. Next, we measured the phosphorylation of mTOR kinase substrates: eIF4E-binding protein-1 (4E-BP1, a direct substrate of mTOR complex-1) and ribosomal S6 (a substrate of S6 kinases downstream of mTOR complex-1). As expected, rapamycin strongly suppressed p-S6 and partially reduced p-4E-BP1.^16,22,23^ In cells treated with **3**, there was no change in substrate phosphorylation.^16^

**Figure 2:**
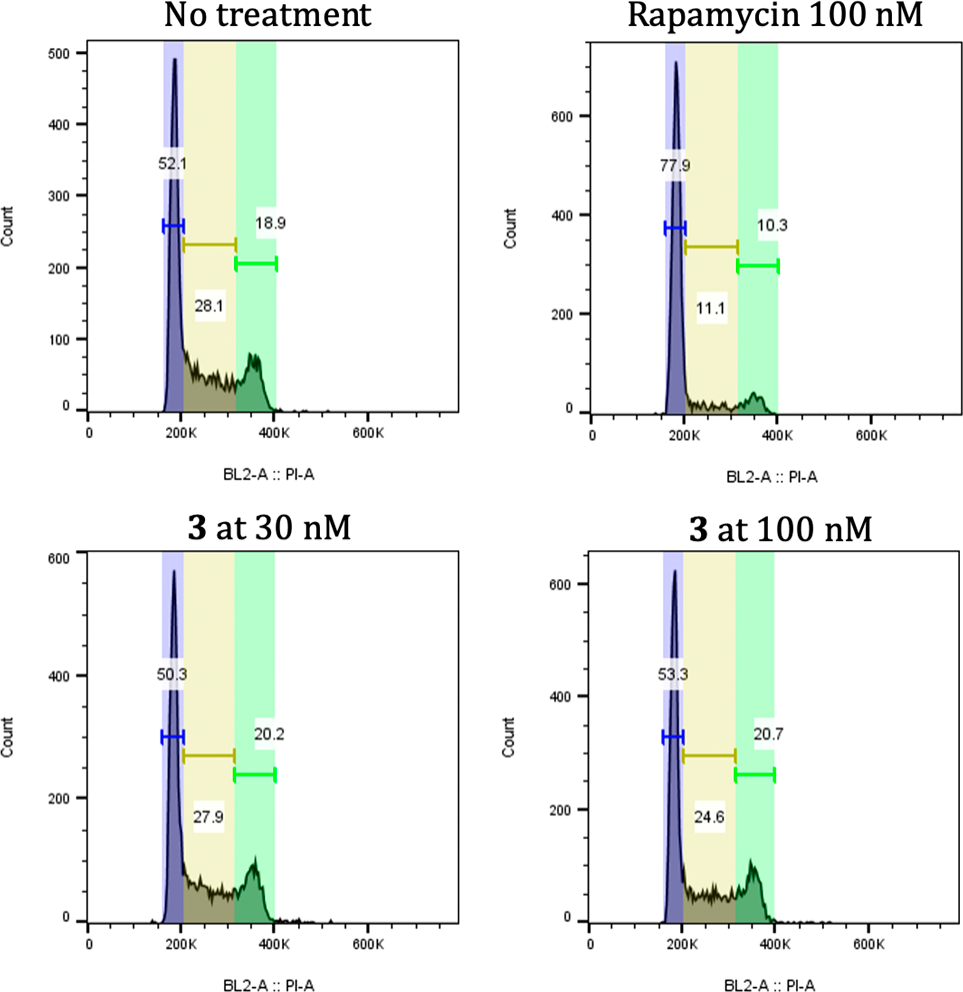
Cell cycle distribution after treatment with no drug, rapamycin as positive control, and brasA analogue **3** at two concentrations.

We have generated a structurally complex analogue of the potent immunosuppressive agent brasilicardin A, whose rigid tricyclic core was designed to mimic that of the natural product and thus to hold the disaccharide and amino acid motifs in a similar orientation. The synthesis leveraged a cobalt-catalyzed, MHAT-initiated radical bicyclization in a particularly complex setting, further demonstrating the utility of this (and other) radical-based polyene cyclizations to make polyfunctional targets. Although the targeted analogue did not show immunosuppressive activity in our biological evaluations, this project did provide new information. While the lack of activity of Jung’s earlier, flexible “brasilogue” **2** was attributed to a lack of rigidity of the scaffold used to space the disaccharide and amino acid structures, we have now shown that rigidity in the spacing unit is not sufficient, and that is in spite of the fact that structural overlay of brasA and our analogue suggested strong structural similarities. Of course, the axial nitrile in our analogue, which is critical to the chemistry we used to generate the molecule, could be the culprit involved in abolishing activity. In any case, our work adds to the minimal available structure–activity relationship information available for brasilicardin A and suggests the possibility that it is a trifunctional molecule, wherein the identity of the diterpenoid core is as critical as the two polar groups at each end.

## Data Availability

The data underlying this study are available in the Supporting Information.

## Supporting Information Available

Experimental procedures, characterization data, NMR spectra for all new compounds, X-ray crystallographic data, computational data, and methods/materials for immunosuppression assay (PDF)

## References

[ref1] Shigemori H.; Komaki H.; Yazawa K.; Mikami Y.; Nemoto A.; Tanaka Y.; Sasaki T.; In Y.; Ishida T.; Kobayashi J.; Brasilicardin A. A Novel Tricyclic Metabolite with Potent Immunosuppressive Activity from Actinomycete *Nocardia brasiliensis*. J. Org. Chem. 1998, 63, 6900–6904. 10.1021/jo9807114. 11672311

[ref2] Komaki H.; Nemoto A.; Tanaka Y.; Takagi H.; Yazawa K.; Mikami Y.; Shigemori H.; Kobayashi J.; Ando A.; Nagata Y.; Brasilicardin A. a New Terpenoid Antibiotic from Pathogenic *Nocardia brasiliensis*: Fermentation, Isolation and Biological Activity. J. Antibiot. 1999, 52, 13–19. 10.7164/antibiotics.52.13. 10092191

[ref3] Yoder R. A.; Johnston J. N. A Case Study in Biomimetic Total Synthesis: Polyolefin Carbocyclizations to Terpenes and Steroids. Chem. Rev. 2005, 105, 4730–4756. 10.1021/cr040623l. 16351060 PMC2575671

[ref4] Anada M.; Hanari T.; Kakita K.; Kurosaki Y.; Katsuse K.; Sunadoi Y.; Jinushi Y.; Takeda K.; Matsunaga S.; Hashimoto S. Total Synthesis of Brasilicardins A and C. Org. Lett. 2017, 19, 5581–5584. 10.1021/acs.orglett.7b02728. 28976203

[ref5] Yoshimura F.; Itoh R.; Torizuka M.; Mori G.; Tanino K. Asymmetric Total Synthesis of Brasilicardins. Angew. Chem., Int. Ed. 2018, 57, 17161–17167. 10.1002/anie.201811403. 30383323

[ref6] Botas A.; Eitel M.; Schwarz P. N.; Buchmann A.; Costales P.; Núñez L. E.; Cortés J.; Morís F.; Krawiec M.; Wolański M.; Gust B.; Rodriguez M.; Fischer W.-N.; Jandeleit B.; Zakrzewska-Czerwińska J.; Wohlleben W.; Stegmann E.; Koch P.; Méndez C.; Gross H. Genetic Engineering in Combination with Semi-Synthesis Leads to a New Route for Gram-Scale Production of the Immunosuppressive Natural Product Brasilicardin A. Angew. Chem., Int. Ed. 2021, 60, 13536–13541. 10.1002/anie.202015852. PMC8251711 33768597

[ref7] Komatsu K.; Tsuda M.; Tanaka Y.; Mikami Y.; Kobayashi J. SAR Studies of Brasilicardin A for Immunosuppressive and Cytotoxic Activities. Bioorg. Med. Chem. 2005, 13, 1507–1513. 10.1016/j.bmc.2004.12.029. 15698766

[ref8] Jung M. E.; Koch P. An Efficient Synthesis of the Protected Carbohydrate Moiety of Brasilicardin A. Org. Lett. 2011, 13, 3710–3713. 10.1021/ol2013704. 21678905

[ref9] Jung M. E.; Chamberlain B. T.; Koch P.; Niazi K. R. Synthesis and Bioactivity of a Brasilicardin A Analogue Featuring a Simplified Core. Org. Lett. 2015, 17, 3608–3611. 10.1021/acs.orglett.5b01712. 26144210

[ref10] Vrubliauskas D.; Vanderwal C. D. Cobalt-Catalyzed Hydrogen-Atom Transfer Induces Bicyclizations That Tolerate Electron-Rich and Electron-Deficient Intermediate Alkenes. Angew. Chem., Int. Ed. 2020, 59, 6115–6121. 10.1002/anie.202000252. PMC7124983 31991035

[ref11] Vrubliauskas D.; Gross B. M.; Vanderwal C. D. Stereocontrolled Radical Bicyclizations of Oxygenated Precursors Enable Short Syntheses of Oxidized Abietane Diterpenoids. J. Am. Chem. Soc. 2021, 143, 2944–2952. 10.1021/jacs.0c13300. 33555176 PMC8112877

[ref12] Johnson L. K.; Niman S. W.; Vrubliauskas D.; Vanderwal C. D. Stereocontrolled Synthesis and Structural Revision of Plebeianiol A. Org. Lett. 2021, 23, 9569–9573. 10.1021/acs.orglett.1c03791. 34851132 PMC8766249

[ref13] aSolladie-Cavallo A.; Koessler J. L. A Four-Step Diastereoselective Synthesis of D-*erythro*-Sphingosine by an Enantioselective Aldol Reaction Using a Titatnium Enolate Derived from a Chiral Iminoglycinate. J. Org. Chem. 1994, 59, 3240–3242. 10.1021/jo00090a052.

[ref14] Mei T.-S.; Wang D.-H.; Yu J.-Q. Expedient Drug Synthesis and Diversification via Ortho-C–H Iodination Using Recyclable PdI_2_ as the Precatalyst. Org. Lett. 2010, 12, 3140–3143. 10.1021/ol1010483. 20550215

[ref15] aSato S.; Tetsuhashi M.; Sekine K.; Miyachi H.; Naito M.; Hashimoto Y.; Aoyama H. Degradation-promoters of cellular inhibitor of apoptosis protein 1 based on bestatin and actinonin. Bioorg. Med. Chem. 2008, 16, 4685–4698. 10.1016/j.bmc.2008.02.024. 18313309

[ref16] Please see the Supporting Information for more information.

[ref17] For measurement of the enantiomeric ratios of these diols, we found that James’s method using 2-formylboronic acid and different enantiomers of 1-phenylethylamine was particularly convenient (see Figure S1): Kelly A. M.; Pérez-Fuertes Y.; Fossey J. S.; Yeste S. L.; Bull S. D.; James T. D. Simple Protocols for NMR Analysis of the Enantiomeric Purity of Chiral Diols. Nat. Protoc. 2008, 3, 215–219. 10.1038/nprot.2007.523. 18274523

[ref18] Fleming P. R.; Sharpless K. B. Selective Transformations of Threo-2,3-Dihydroxy Esters. J. Org. Chem. 1991, 56, 2869–2875. 10.1021/jo00008a051.

[ref19] Jeffery T. On the Efficiency of Tetraalkylammonium Salts in Heck Type Reactions. Tetrahedron 1996, 52, 10113–10130. 10.1016/0040-4020(96)00547-9.

[ref20] Ley S. V.; Baeschlin D. K.; Dixon D. J.; Foster A. C.; Ince S. J.; Priepke H. W. M.; Reynolds D. J. 1,2-Diacetals: A New Opportunity for Organic Synthesis. Chem. Rev. 2001, 101 (1), 53–80. 10.1021/cr990101j. 11712194

[ref21] Schmidt R. R.; Michel J. Facile Synthesis of α- and β-*O*-Glycosyl Imidates: Preparation of Glycosides and Disaccharides. Angew. Chem., Int. Ed. 1980, 19, 731–732. 10.1002/anie.198007311.

[ref22] Saxton R. A.; Sabatini D. M. mTOR Signaling in Growth, Metabolism, and Disease. Cell 2017, 168, 960–976. 10.1016/j.cell.2017.02.004. 28283069 PMC5394987

[ref23] Lee J.-H. S.; Vo T.-T.; Fruman D. A. Targeting mTOR for the treatment of B cell malignancies. Br. J. Clin. Pharmacol. 2016, 82, 1213–1228. 10.1111/bcp.12888. 26805380 PMC5061788

