# Supplementary material for: Synthesis of a Complex Brasilicardin Analogue Utilizing a Cobalt-Catalyzed MHAT-Induced Radical Bicyclization Reaction
Source: Org Lett. 2023 May 4;25(19):3451–5. doi: 10.1021/acs.orglett.3c01019 (PMC10204089; doi:10.1021/acs.orglett.3c01019)
Supplement: Supplementary file 1 — ol3c01019_si_001.pdf [file ol3c01019_si_001.pdf]

Synthesis of a Complex Brasilicardin Analogue Utilizing a Cobalt-Catalyzed MHAT-Induced Radical Bicyclization Reaction

Scott W. Niman[1], Roberta Buono[2], David Fruman[2], Christopher D. Vanderwal[1,3,*]

[1]Department of Chemistry, 1102 Natural Sciences II, University of California, Irvine, CA 92697-2025, USA.

[2]Department of Molecular Biology & Biochemistry, 3205 McGaugh Hall, University of California, Irvine, CA 92697-2525, USA.

[3]Department of Pharmaceutical Sciences, 101 Theory #100, University of California, Irvine, CA 92617, USA.

cdv@uci.edu

Supporting Information

Table of Contents:

I. Experimental Information:

Materials and Methods..**S3**

Experimental Procedures and Characterization Data..**S5**

Method for Computing Lowest Energy Conformers..**S23**

PyMOL Overlays of the Aglycones for Structures **1**, **2**, and **3**..**S29**

Immunosuppression Assay Methods and Materials..**S30**

II. References...**S32**

III. Experimental Data:

X-ray Data for **27**...**S34**

1D and 2D NMR Spectra...**S55**

I. Experimental Information

Materials and Methods

All reactions were performed in oven-dried (120 °C) or flame-dried glassware under an atmosphere of dry argon unless otherwise noted. Reaction solvents including dichloromethane (CH_2Cl_2, Fisher, HPLC Grade), hexanes (Fisher, HPLC Grade), diethyl ether (Et_2O, Fisher, BHT stabilized, HPLC Grade), benzene (C_6H_6, Fisher, HPLC Grade), tetrahydrofuran (THF, Fisher, HPLC Grade), and toluene ($PhCH_3$, Fisher, HPLC Grade) were dried by percolation through a column packed with neutral alumina and a column packed with Q5 reactant, a supported copper catalyst for scavenging oxygen, under a positive pressure of argon.

Solvents for workup and chromatography were: hexanes (Fisher or EMD, ACS Grade), EtOAc (Fisher, ACS Grade), dichloromethane (CH_2Cl_2, Fisher, ACS Grade), and diethyl ether (Fisher, ACS Grade). Column chromatography was performed using EMD Millipore 60 Å (0.040–0.063 mm) mesh silica gel (SiO_2). Analytical and preparatory thin-layer chromatography was performed on Merck silica gel 60 F254 TLC plates. Visualization was accomplished with UV (254 or 210 nm), and p-anisaldehyde, vanillin, potassium permanganate, 2,4-dinitrophenylhydrazine, or ceric ammonium molybdate and heat as developing agents. Chloroform-d ($CDCl_3$, 99.8% D, DLM-7) and Dimethyl sulfoxide-D6 (99.9% D, DLM-10) were purchased from Cambridge Isotope Laboratories. K_2CO_3 (anhydrous, 99%, Alfa Aesar), $NaHCO_3$ (ACS grade, Fisher), NaOH (ACS grade, Macron or Fisher), $Na_2S_2O_3$ (ACS grade, Fisher), 1-Fluoro-2,4,6-trimethylpyridinium tetrafluoroborate (TCI or Sigma Aldrich, 95-99%) were purchased and used without further purification. Triethylamine (EMD, CaH_2) and pyridine (Alfa Aesar, CaH_2) were distilled from the indicated drying agents prior to use.

Proton and carbon magnetic resonance spectra (^1H NMR and ^{13}C NMR) were recorded at 298K on a Bruker CRYO500 (500 MHz, ^1H; 125 MHz, ^{13}C) or a Bruker AVANCE600 (600 MHz, ^1H; 151 MHz, ^{13}C) spectrometer with solvent residual as the internal standard for ^1H NMR: $CHCl_3$ at 7.27 ppm, and solvent as internal standard for ^{13}C NMR: $CDCl_3$ at 77.16 ppm. ^1H NMR data are reported as follows: chemical shift, multiplicity (s = singlet, d = doublet, t = triplet, q = quartet, dd = doublet of doublets, ddd

= doublet of doublet of doublets, td = triplet of doublets, tdd = triplet of doublet of doublets, qd = quartet of doublets, m = multiplet, br. s. = broad singlet), coupling constants (Hz), and integration. High resolution mass spectra (HRMS) were recorded on a Waters LCT Premier S2 spectrometer using ESI-TOF (electrospray ionization-time of flight) and data are reported in the form of (m/z). Optical rotation was measured on a Jasco P-1010 polarimeter with an optical path of 5 cm at 22-25 °C with the measurement performed in $CHCl_3$.

Experimental Procedures and Characterization Data

*Aldehyde Fragment Synthesis (**20**):*

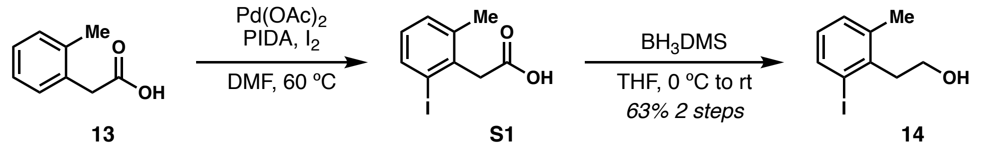

The following procedure was adapted from Yu:[1] To a 250 mL Ace tube was added phenylacetic acid (**13**) (4.0 g, 26.6 mmol, 1.0 equiv.), $Pd(OAc)_2$ (299 mg, 1.33 mmol, 0.05 equiv.), PIDA (9.43 g, 29.29 mmol,1.1 equiv.), and iodine (7.43 g, 29.29 mmol, 1.1 equiv.) open to air at room temperature. The flask was then charged with dry DMF (76 mL, 0.35M), sealed, and wrapped in aluminum foil to exclude light. The pressure vessel was then submerged in a preheated 60 ºC oil bath. After 48 hours, the reaction was allowed to cool to room temperature and diluted with EtOAc (300 mL). The mixture was transferred to a separatory funnel and washed with saturated aqueous sodium thiosulfate (3 x 50 mL), H_2O (3 x 50 mL), and brine (3 x 50 mL). The organic extract was then dried over $MgSO_4$, filtered, and concentrated *in vacuo*. The resulting yellowish solid (11.9 g) was used in the next step without further purification. *Spectral data for **S1** matched those reported by Yu.*

To the crude acid **S1** (ca. 26.6 mmol, 11.9 g) in THF (52 mL) at 0 ºC was added BH_3DMS (26.0 mL, 52.0 mmol, 2.0 M in THF, 2.0 equiv.) slowly. After 30 minutes, the ice bath was removed and allowed to warm to room temperature overnight. After 19 hours, an NMR aliquot indicated consumption of the starting material and the reaction mixture was cooled to 0 ºC. The excess borane was quenched with MeOH (30 mL) slowly (CAUTION!) then allowed to warm to room temperature for 30 minutes. The solution was concentrated and the resulting crude residue was purified by column chromatography (SiO_2, 10:1 then 1:1 hexanes:EtOAc) to afford **14** (4.40 g, 63% yield over 2 steps) as a colorless solid.

^{1}H NMR (600 MHz, $CDCl_3$) δ 7.70 (d, J = 7.8 Hz, 1H), 7.14 (d, J = 7.5 Hz, 1H), 6.81 (t, J = 7.7 Hz, 1H), 3.82 (t, J = 7.5 Hz, 2H), 3.14 (t, J = 7.5 Hz, 2H), 2.44 (s, 3H).
^{13}C NMR (126 MHz, $CDCl_3$) δ 139.0, 138.5, 137.8, 130.7, 128.4, 102.6, 61.5, 41.0, 21.5.
HRMS (CI+) *m/z* calc'd for $C_9H_{11}IO$ $[M]^+$: 261.9855, found 261.9847.
TLC: R_f = 0.27 (3:1 hexanes:EtOAc, visualized by $KMnO_4$).

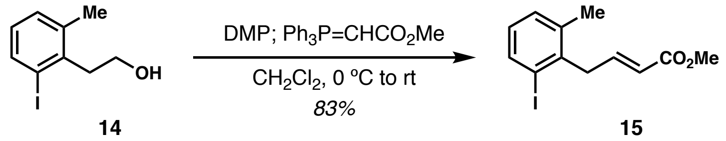

No attempt was made to exclude air or moisture. To the carbinol **14** (2.02 g, 7.71 mmol, 1.0 equiv.) in CH_2Cl_2 (26 mL) at 0 ºC was added DMP (3.92 g, 9.23 mmol, 1.2 equiv.) in portions. The ice bath was removed after complete addition of the periodinane. After 40 minutes, TLC analysis indicated consumption of the starting carbinol and formation of a

less polar compound. The reaction mixture was cooled to 0 ºC, treated with a solution of the Wittig reagent (3.87 g, 11.56 mmol, 1.5 equiv.) in CH_2Cl_2 (13 mL) and then allowed to warm to room temperature. After 1 hour, the reaction mixture was concentrated and subsequently diluted with Et_2O (30 mL). The heterogeneous solution was filtered through a short SiO_2 plug eluting with Et_2O (2 x 15 mL) to remove excess triphenylphosphine oxide. The resulting yellow filtrate was concentrated, and the resulting crude oil was purified by column chromatography (SiO_2, 20:1 then 9:1 hexanes:EtOAc) to afford **15** (2.03 g, 83% yield) as a light yellow oil.

^{1}H NMR (500 MHz, $CDCl_3$) δ 7.72 (d, *J* = 7.8 Hz, 1H), 7.14 (d, *J* = 7.4 Hz, 1H), 7.04 (dt, *J* = 15.7, 5.7 Hz, 1H), 6.85 (t, *J* = 7.7 Hz, 1H), 5.67 (dt, *J* = 15.7, 1.9 Hz, 1H), 3.77 (dd, *J* = 5.7, 2.0 Hz, 2H), 3.72 (s, 3H), 2.34 (s, 3H).
^{13}C NMR (126 MHz, $CDCl_3$) δ 167.0, 145.1, 138.5, 138.3, 137.9, 130.8, 128.8, 121.9, 102.5, 51.6, 40.9, 21.2.
HRMS (ES+) *m/z* calc'd for $C_{12}H_{13}IO_2$ $[M+Na]^+$: 338.9858, found 338.9857.
TLC: R_f = 0.47 (1:1 hexanes:EtOAc, visualized by $KMnO_4$).

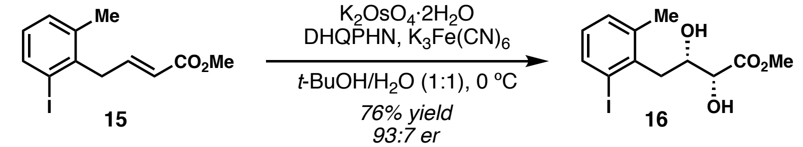

To a 250 mL round bottomed flask was added $K_2OsO_4{\bullet}2H_2O$ (34.1 mg, 0.0927 mmol, 0.7 mol%), DHQPHN (86.5 mg, 0.172 mmol, 1.3 mol%), $H_3CSO_2NH_2$ (1.26 g, 13.25 mmol, 1.0 equiv.), K_2CO_3 (5.49 g, 39.75 mmol, 3.0 equiv.), $NaHCO_3$ (3.34 g, 39.75 mmol, 3 equiv.), and $K_3Fe(CN)_6$ (13.08 g, 39.75 mmol, 3.0 equiv.) at room temperature and open to air. The flask was then charged with water (67 mL) and t-BuOH (57 mL) and stirred vigorously to form a homogenous biphasic mixture (ca. 15 minutes). The flask was then submerged in an ice bath until salts began to precipitate. Alkene **15** (4.19 g, 13.25 mmol, 1.0 equiv.) was added slowly and rinsed into the reaction flask with *t*-BuOH (10 mL). At this point the reaction mixture was moved to a Cryocool set to 0 ºC and maintained at this temperature for 96 hours with vigorous stirring. Then, an additional 0.1 mol% $K_2OsO_42H_2O$ (4.9 mg) was added and reacted for a further 24 hours. After a total of 120 hours, the reaction was quenched with Na_2SO_3 (ca. 8 g) and stirred vigorously while warming to room temperature over an hour. The mixture was diluted with EtOAc (40 mL) and the layers were separated. The aqueous phase was extracted with EtOAc (4 x 40 mL) and the combined organic extracts were dried over $MgSO_4$, filtered, and concentrated *in vacuo*. The resulting crude residue was purified by column chromatography (SiO_2, 3:2 then 2:3 hexanes:EtOAc) to afford **16** (3.52 g, 76% yield) as a slight yellow solid. The absolute configuration of diol **16** was assumed based on the mnemonic rule for the facial selectivity of the Sharpless dihydroxylation reaction[2] and later confirmed with X-ray crystallography.

^{1}H NMR (500 MHz, $CDCl_3$) δ 7.72 (d, *J* = 7.6 Hz, 1H), 7.15 (d, *J* = 7.6 Hz, 1H), 6.83 (t, *J* = 7.7 Hz, 1H), 4.33 (ddd, *J* = 8.6, 5.2, 1.9 Hz, 1H), 4.16 (d, *J* = 2.0 Hz, 1H), 3.83 (s, 3H), 3.26 (dd, *J* = 14.1, 8.6 Hz, 1H), 3.22 (brs, 1H), 3.12 (dd, *J* = 14.1, 5.3 Hz, 1H), 2.45 (s, 2H), 2.11 (brs, 1H).
^{13}C NMR (126 MHz, $CDCl_3$) δ 173.8, 139.2, 138.8, 138.0, 131.0, 128.6, 103.1, 72.8, 72.3, 53.1, 41.2, 21.6.
HRMS (ES+) *m/z* calc'd for $C_{12}H_{15}IO_4$ $[M+Na]^+$: 372.9913, found 372.9940.
TLC: R_f = 0.47 (1:1 hexanes:EtOAc, visualized by $KMnO_4$).
$[\alpha]^{21.8}_D$ –30.6 (c = 1.01, $CHCl_3$).

The method reported by James[3] was performed on enantiomerically enriched diol **16** made by Sharpless asymmetric dihydroxylation. The diol (**16**) was treated separately with both enantiomers of 1-phenylethylamine as chiral resolving agents and analyzed by ^{1}H NMR. Integration of the quartets at 5.44 ppm and 5.37 ppm of the diastereomeric boronate esters indicated that the enantiomeric ratio of **16** was approximately 93:7 er (Figure **S1**).

*Note: While Mosher's analysis of the bis-ester can be used to obtain enantiopurity, we found the James method to be more reliable with **16** and quicker to perform.*

A general procedure is detailed below:

Diol **16** (35 mg, 0.1 mmol), 2-formylphenylboronic acid (15.7 mg, 0.105 mmol), and 4Å mol sieves (100 mg) were combined in a 20 mL vial at room temperature and open to air. Next, the vial was charged with $CDCl_3$ (2.0 mL) and then (*R*)-1-phenylethylamine (0.02 mL, 0.150 mmol) was added dropwise. After 30 minutes, approximately 1.0 mL of the reaction mixture was taken up in a syringe and passed through an HPLC filter into an NMR tube. Analysis of the ^{1}H NMR spectrum indicated diastereomeric ratios which correspond to the enantiopurity of diol **16**. This procedure was also performed with (*S*)-1-phenylethylamine to obtain the alternative diastereomeric complex for comparison.

Figure S1. Comparison of the boronate esters generated from both enantiomers of 1-phenylethylamine complexed with 2-formylphenylboronic acid and diol **16**.

Boronate complex of **16** with (*R*)-phenethylamine (ca. 93:7 er)

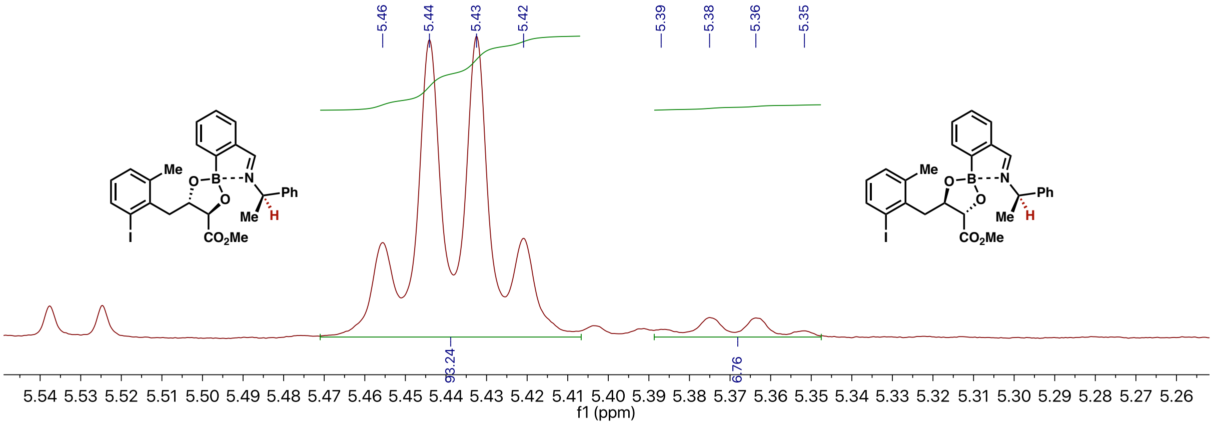

Boronate complex of **16** with (*S*)-phenethylamine (ca. 93:7 er)

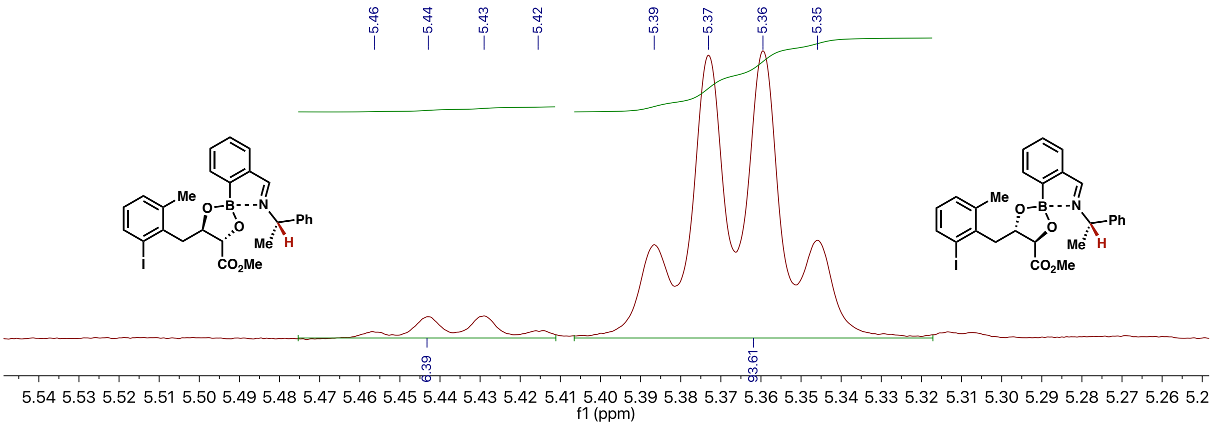

We evaluated a variety of ligands for the dihydroxylation of **15**, finding that most of the commonly used ligands provided poor enantioselectivity to **16** (**Table S1**). After extensive screening, we discovered that the monomeric phenanthrene alkaloid DHQPHN produced reasonable enantiopurity, which could be further improved by tailoring the osmium and ligand loadings.

Note: all of the enantiomeric ratios were obtained via the corresponding boronate complexes as shown in **Figure S1**.

Table S1. Sharpless asymmetric dihydroxylation optimization

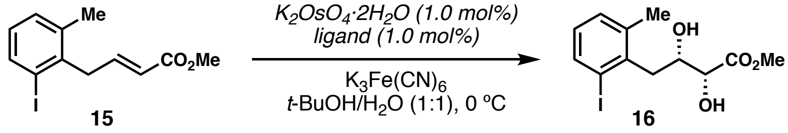

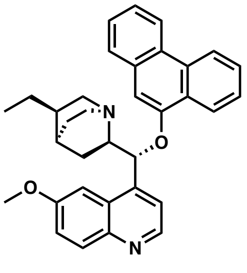

DHQPHN

Entry	*ligand*	*er*
1	$(DHQ)_2PHAL$	83:17
2	$(DHQ)_2AQN$	81:19
3*	$(DHQ)_2AQN$	81:19
4	$(DHQ)_2PYR$	72:28
5	DHQPHN	88:12
**6****	**DHQPHN**	**93:7**

* = 5.0 mol% ligand
** = 0.7 mol% osmium and 1.3 mol% ligand

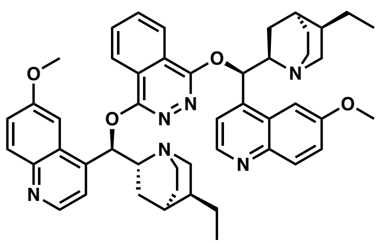

$(DHQ)_2PHAL$

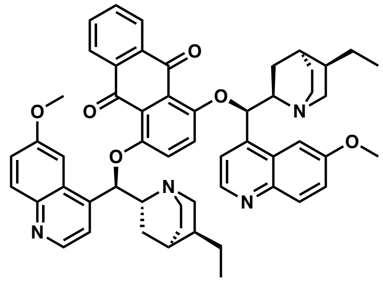

$(DHQ)_2AQN$

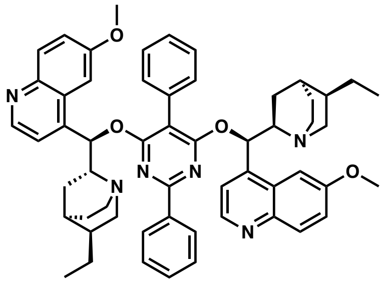

$(DHQ)_2PYR$

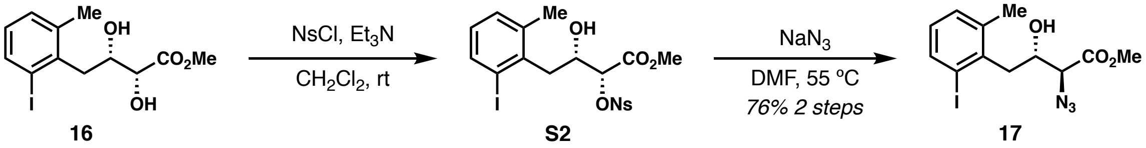

To a solution of diol **16** (3.45 g, 9.85 mmol) in CH_2Cl_2 (65 mL) at 0 ºC and open to air was added Et_3N (2.1 mL, 14.78 mmol, 1.5 equiv.) followed by *p*-NsCl (2.29 g, 10.34 mmol, 1.05 equiv.) in portions. After complete addition, the ice bath was removed, and the reaction was allowed to warm to room temperature. After 3 hours, the mixture was diluted with CH_2Cl_2 (50 mL) and washed with 1M HCl (30 mL) and sat. aq. $NaHCO_3$ (20 mL). The solution was dried over $MgSO_4$, filtered, and concentrated *in vacuo*. Analysis of the crude ^{1}H NMR spectrum indicated approximately 90% conversion and the crude yellow solids (6.2 g) were used in the next step without further purification. A small portion of the crude mixture was purified (SiO_2, 3:1 hexanes:EtOAc) for analytical purposes.

*Analytical sample of nosylate **S2**:*
^{1}H NMR (500 MHz, $CDCl_3$) δ 8.43 (d, J = 8.9 Hz, 2H), 8.24 (d, J = 8.9 Hz, 2H), 7.71 (d, J = 8.0 Hz, 1H), 7.15 (d, J = 7.5 Hz, 1H), 6.84 (t, J = 7.7 Hz, 1H), 5.23 (d, J = 3.2 Hz, 1H),

4.52 (ddt, J = 10.4, 6.9, 2.9 Hz, 1H), 3.74 (s, 3H), 3.26 (dd, J = 14.3, 10.8 Hz, 1H), 2.95 (dd, J = 14.4, 2.5 Hz, 1H), 2.41 (s, 3H), 2.06 – 1.99 (m, 1H).
^{13}C NMR (125 MHz, $CDCl_3$) δ 167.1, 151.0, 142.1, 139.3, 138.0, 137.8, 131.0, 129.7 (2C), 129.0, 124.5 (2C), 102.9, 81.2, 71.4, 53.2, 40.3, 21.7.
HRMS (ES+) *m/z* calc'd for $C_{18}H_{18}INO_8S$ $[M+Na]^+$: 557.9695, found 557.9698.
TLC: R_f = 0.30 (3:1 hexanes:EtOAc, visualized by $KMnO_4$).
$[\alpha]^{22.4}_D$ –51.8 (c = 1.00, $CHCl_3$).

To the crude nosylate **S2** (6.2 g) in DMF (20 mL) at room temperature and open to air was added NaN_3 (3.20 g, 49.25 mmol, 5.0 equiv.) in one portion. The flask was fitted with a rubber septum, the headspace was purged with Ar, and the mixture was heated to 55 ºC. After 19 hours, the reaction mixture was cooled to rt and diluted with EtOAc (120 mL) and H_2O (50 mL). The biphasic mixture was transferred to a separatory funnel and the layers were separated. The organic layer was washed successively with brine (3 x 25 mL), dried over $MgSO_4$, filtered, and concentrated *in vacuo*. The resulting crude residue was purified by column chromatography (SiO_2, 5:1 hexanes:EtOAc) to afford **17** (2.81 g, 76% yield 2 steps) as a slight yellow oil. A small quantity of diol **16** was also recovered (570 mg, 16% of mass balance).

^{1}H NMR (500 MHz, $CDCl_3$) δ 7.72 (d, *J* = 7.9 Hz, 1H), 7.16 (d, *J* = 7.5 Hz, 1H), 6.84 (t, *J* = 7.7 Hz, 1H), 4.44 – 4.37 (m, 1H), 4.19 (d, *J* = 5.1 Hz, 1H), 3.87 (s, 3H), 3.24 (dd, *J* = 14.2, 10.5 Hz, 1H), 3.03 (dd, *J* = 14.2, 3.2 Hz, 1H), 2.43 (s, 3H), 2.19 (d, *J* = 6.0 Hz, 1H).
^{13}C NMR (125 MHz, $CDCl_3$) δ 169.1, 139.4, 138.3, 138.0, 131.0, 128.8, 102.8, 72.0, 66.5, 53.0, 40.3, 21.7.
HRMS (ES+) *m/z* calc'd for $C_{12}H_{14}IN_3O_3$ $[M+Na]^+$: 397.9978, found 397.9978.
TLC: R_f = 0.42 (3:1 hexanes:EtOAc, visualized by $KMnO_4$).
$[\alpha]^{22.0}_D$ –27.2 (c = 1.01, $CHCl_3$).

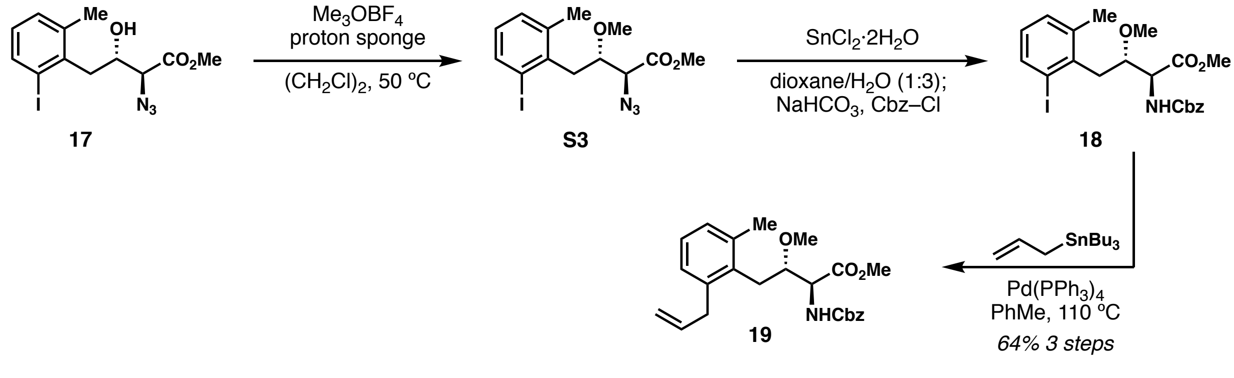

To a solution of azide **17** (2.81 g, 7.48 mmol, 1.0 equiv.) in $(CH_2Cl)_2$ (15 mL) at room temperature was added sequentially Proton Sponge (3.52 g, 16.45 mmol, 2.2 equiv.) and Me_3OBF_4 (1.99 g, 13.46 mmol, 1.8 equiv.). The reaction flask was then submerged in a 50 ºC preheated oil bath and allowed to react at this temperature overnight. After 19 hours, the mixture was cooled to room temperature. TLC analysis indicated consumption of the starting material and the reaction was quenched with H_2O (40 mL), 1M HCl (20 mL), and diluted with EtOAc (100 mL). The biphasic mixture was filtered over celite, and

the layers were separated. The aqueous phase was extracted with EtOAc (50 mL) and the combined organic extracts were washed with 1M HCl (2 x 20 mL) and sat. aq. $NaHCO_3$ (20 mL). The organic solution was dried over $MgSO_4$, filtered, and concentrated *in vacuo*. The resulting crude methyl ether **S3** (3.1 g) was used in the next step without purification. An analytical sample was obtained by column chromatography (SiO_2, 3:1 hexanes:EtOAc) for characterization purposes.

*Analytical sample of methyl ether **S3**:*
^{1}H NMR (500 MHz, $CDCl_3$) δ 7.70 (d, J = 8.0 Hz, 1H), 7.14 (d, J = 7.5 Hz, 1H), 6.82 (t, J = 7.6 Hz, 1H), 4.23 (d, J = 4.4 Hz, 1H), 4.05 (ddd, J = 10.1, 4.4, 3.4 Hz, 1H), 3.82 (s, 3H), 3.32 (dd, J = 14.1, 10.2 Hz, 1H), 3.13 (s, 3H), 2.90 (dd, J = 14.2, 3.5 Hz, 1H), 2.45 (s, 3H).
^{13}C NMR (125 MHz, $CDCl_3$) δ 168.8, 140.0, 138.6, 137.8, 130.8, 128.5, 102.3, 81.9, 64.4, 59.4, 52.9, 39.0, 21.8.
HRMS (ES+) *m/z* calc'd for $C_{13}H_{16}IN_3O_3$ $[M+Na]^+$: 412.0134, found 412.0148.
TLC: R_f = 0.61 (2:1 hexanes:EtOAc, visualized by $KMnO_4$).
$[\alpha]^{22.4}_D$ –15.6 (c = 1.00, $CHCl_3$).

To the crude azide **S3** (3.1 g) in dioxane/H_2O (1:3, 50 mL) at 0 ºC was added $SnCl_2{\bullet}2H_2O$ (8.44 g, 37.4 mmol, 5.0 equiv.) in small portions. After complete addition, the ice bath was removed and allowed to warm to room temperature. After 4 hours, TLC analysis indicated consumption of the starting material and the mixture was cooled to 0 ºC. The reaction was quenched with sat. aq. $NaHCO_3$ (ca. 80 mL) to a pH of ~8.5, then Cbz–Cl (1.6 mL, 11.22 mmol, 1.5 equiv.) was added slowly and the ice bath was removed. After 18 hours, the brown heterogenous mixture was diluted with EtOAc (50 mL) and then filtered over celite rinsing with EtOAc (2 x 50 mL). The filtrate was transferred to a separatory funnel and the layers were separated. The aqueous phase was extracted with EtOAc (20 mL) and the combined organic extracts were washed with 1M HCl (100 mL). The HCl wash was back extracted with EtOAc (2 x 20 mL) and the combined organic extracts were dried over $MgSO_4$, filtered, and concentrated *in vacuo*. The resulting crude residue was purified by column chromatography (SiO_2, 9:1 hexanes:EtOAc) to afford **18** (3.4 g) contaminated with ~40 mol% Cbz–Cl which was used without further purification. An analytical sample was obtained by column chromatography (SiO_2, 9:1 then 5:1 hexanes:EtOAc) for characterization purposes.

*Analytical sample of carbamate **18**:*
^{1}H NMR (500 MHz, $CDCl_3$) δ 7.69 (d, J = 7.9 Hz, 1H), 7.41 – 7.31 (m, 5H), 7.12 (d, J = 7.4 Hz, 1H), 6.81 (t, J = 7.7 Hz, 1H), 5.60 (d, J = 8.9 Hz, 1H), 5.16 (s, 2H), 4.72 (dd, J = 8.8, 4.2 Hz, 1H), 3.94 – 3.86 (m, 1H), 3.82 (s, 3H), 3.23 (dd, J = 14.2, 10.7 Hz, 1H), 3.09 (s, 3H), 2.86 (dd, J = 14.3, 2.9 Hz, 1H), 2.41 (s, 3H).
^{13}C NMR (125 MHz, $CDCl_3$) δ 170.9, 156.1, 139.7, 139.0, 137.8, 136.3, 130.7, 128.7 (2C), 128.4, 128.4, 128.3 (2C), 102.3, 82.2, 67.3, 59.7, 56.9, 52.7, 39.4, 21.8.
HRMS (ES+) *m/z* calc'd for $C_{21}H_{24}INO_5$ $[M+Na]^+$: 520.0597, found 520.0585.
TLC: R_f = 0.39 (3:1 hexanes:EtOAc, visualized by $KMnO_4$).
$[\alpha]^{22.3}_D$ +9.1 (c = 1.04, $CHCl_3$).

To a solution of crude iodide **18** (3.4 g) and allyl tributyltin (4.65 mL, 15.0 mmol) in PhMe (60 mL) open to air was added $Pd(PPh_3)_4$ (693 mg, 0.6 mmol). The flask was fitted with a rubber septum promptly, and the headspace was purged with Ar. The reaction was heated to 110 ºC in a preheated oil bath where the color gradually changed from vibrant yellow to orange to dark brown over the course of an hour. After a total of 3.5 hours, the reaction was cooled to room temperature and concentrated *in vacuo*. The resulting crude residue was purified by column chromatography (SiO_2, 5:1 hexanes:EtOAc) to afford impure **19** (2.8 g) as a dark orange oil. A second purification (SiO_2, 9:1 hexanes:EtOAc) afforded pure **19** (1.96 g, 64% over 3 steps) as a yellow viscous oil. *Note: although two purifications were performed, we were not able to completely remove all presumed tin-containing byproducts (ca. 10% impurity) from this reaction.*

^{1}H NMR (500 MHz, $CDCl_3$) δ 7.44 – 7.31 (m, 5H), 7.14 – 6.97 (m, 3H), 5.94 (tt, J = 12.5, 5.1 Hz, 1H), 5.58 (d, J = 8.7 Hz, 1H), 5.16 (s, 2H), 5.05 (dd, J = 10.2, 1.7 Hz, 1H), 4.96 (d, J = 17.1 Hz, 1H), 4.71 (dd, J = 8.7, 3.8 Hz, 1H), 3.83 (s, 3H), 3.73 (d, J = 10.7 Hz, 1H), 3.54 (dd, J = 15.9, 6.0 Hz, 1H), 3.38 (dd, J = 15.9, 6.0 Hz, 1H), 3.10 (s, 3H), 3.08 – 3.03 (m, 1H), 2.70 (dd, J = 14.5, 2.5 Hz, 1H), 2.33 (s, 3H).
^{13}C NMR (125 MHz, $CDCl_3$) δ 171.0, 156.1, 139.1, 137.7, 137.5, 136.3, 135.1, 128.7, 128.7 (2C), 128.4, 128.3 (2C), 127.8, 126.6, 115.7, 83.0, 67.3, 59.6, 57.1, 52.6, 37.6, 31.1, 20.6.
HRMS (ES+) *m/z* calc'd for $C_{24}H_{29}NO_5$ $[M+Na]^+$: 434.1943, found 434.1929.
TLC: R_f = 0.38 (3:1 hexanes:EtOAc, visualized by $KMnO_4$).
$[\alpha]^{22.3}_D$ +15.4 (c = 1.02, $CHCl_3$).

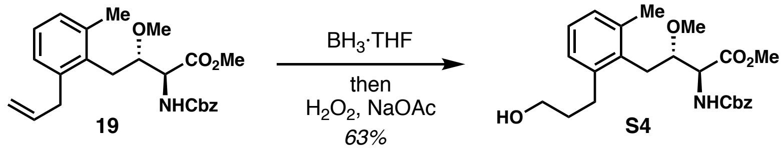

To a solution of alkene **19** (320 mg, 0.777 mmol) In THF (3.8 mL) at 0 ºC was added BH_3THF (1.55 mL, 1.55 mmol, 1.0 M in THF) slowly. After 5 minutes following complete addition of the borane reagent, the ice bath was removed and allowed to warm to room temperature (the color gradually changes from pale yellow to brown). After 75 minutes, TLC analysis indicated consumption of the starting material and the solution was cooled to 0 ºC. The reaction was quenched cautiously with MeOH (10 mL) and then NaOAc (255 mg) was added in one portion followed by H_2O_2 (1.5 mL, 30% solution in water). The biphasic mixture was stirred for 1 hour at room temperature then diluted with EtOAc (10 mL) and H_2O (10 mL). The layers were separated, and the aqueous phase was extracted with EtOAc (2 x 10 mL). The combined organic extracts were dried over $MgSO_4$, filtered, and concentrated *in vacuo*. The resulting crude residue was purified by column chromatography (SiO_2, 3:2 hexanes:EtOAc) to afford **S4** (210 mg, 63% yield) as a pale yellow viscous oil. *Note: this compound was contaminated with presumed tin-containing byproducts (ca. 10% impurity) and was used in the next step without further purification.*

^{1}H NMR (500 MHz, $CDCl_3$) δ 7.43 – 7.31 (m, 5H), 7.12 – 6.94 (m, 3H), 5.79 (d, J = 8.2 Hz, 1H), 5.16 (s, 2H), 4.67 (dd, J = 8.2, 3.8 Hz, 1H), 3.83 (s, 3H), 3.78 – 3.72 (m, 1H),

3.73 – 3.63 (m, 2H), 3.13 (m, 1H *overlapping*), 3.08 (s, 3H), 2.85 (dq, J = 15.7, 7.9 Hz, 1H), 2.78 (d, J = 14.5 Hz, 1H), 2.74 – 2.65 (m, 1H), 2.34 (s, 3H), 1.86 – 1.78 (m, 2H), 1.66 (brs, 1H).
^{13}C NMR (125 MHz, $CDCl_3$) δ 170.9, 156.2, 141.2, 137.6, 136.3, 134.9, 128.7 (2C), 128.4 (2C), 128.2 (2C), 127.3, 126.6, 83.3, 67.3, 62.5, 59.9, 57.6, 52.7, 34.3, 31.3, 29.3, 20.6.
HRMS (ES+) *m/z* calc'd for $C_{24}H_{31}NO_6$ $[M+H]^+$: 430.2230, found 430.2229
TLC: R_f = 0.30 (1:1 hexanes:EtOAc, visualized by $KMnO_4$).
$[\alpha]^{21.8}_D$ +16.0 (c = 1.00, $CHCl_3$).

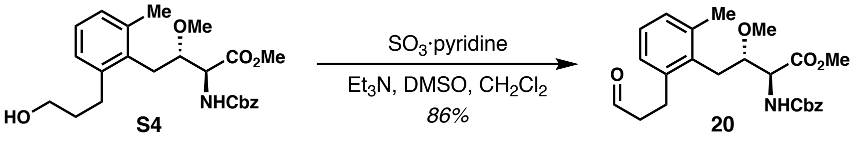

To a solution of carbinol **S4** (38.1 mg, 0.0887 mmol) in CH_2Cl_2 (0.9 mL) at room temperature and open to air was added DMSO (0.12 mL, 1.77 mmol) and Et_3N (0.12 mL, 0.887 mmol). Next, SO_3•pyridine complex (70.5 mg, 0.443 mmol) was added in one portion and the homogenous solution was reacted for one hour. At this point, TLC analysis indicated consumption of the starting material and the reaction was quenched with sat. aq. $NaHCO_3$ (5 mL) and transferred to a separatory funnel. The aqueous phase was extracted with CH_2Cl_2 (3 x 5 mL) and the combined organic extracts were dried over $MgSO_4$, filtered, and concentrated *in vacuo*. The crude residue was then filtered through a short pH 7 SiO_2 plug eluting with EtOAc (5 mL) which provided **20** (32.8 mg, 86%) as a colorless viscous oil in suitable purity (>90%). The aldehyde was used promptly in the subsequent alkenylation reaction without further purification.

^{1}H NMR (500 MHz, $CDCl_3$) δ 9.81 (s, 1H), 7.42 – 7.32 (m, *J* = 21.9, 4.8 Hz, 5H), 7.13 – 6.87 (m, 3H), 5.62 (d, *J* = 8.2 Hz, 1H), 5.16 (s, 2H), 4.70 (dd, *J* = 8.4, 3.6 Hz, 1H), 3.83 (s, 3H), 3.71 (d, *J* = 11.4 Hz, 1H), 3.16 – 3.00 (m, 5H), 2.95 (dq, *J* = 13.9, 7.2, 6.5 Hz, 1H), 2.79 – 2.63 (m, 3H), 2.33 (s, 3H).
^{13}C NMR (125 MHz, $CDCl_3$) δ 201.8, 170.9, 156.1, 139.8, 137.6, 136.3, 134.8, 128.9, 128.7 (2C), 128.4, 128.3 (2C), 127.0, 126.8, 82.9, 67.4, 59.7, 57.1, 52.8, 45.2, 31.1, 25.4, 20.6.
HRMS (ES+) *m/z* calc'd for $C_{24}H_{29}NO_6$ $[M+Na]^+$: 450.1893, found 450.1892.
TLC: R_f = 0.60 (1:1 hexanes:EtOAc, visualized by CAM).
$[\alpha]^{22.3}_D$ +16.8 (c = 1.04, $CHCl_3$).

*Cyanophosphonate Fragment Synthesis (**24**):*

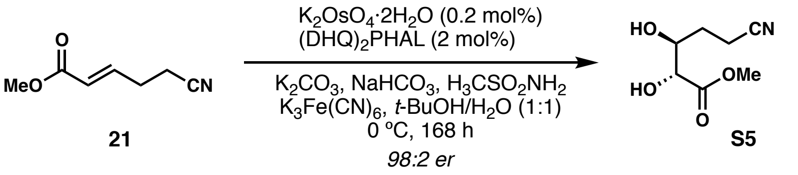

To a 250 mL round-bottomed flask at room temperature and open to air was added $K_2OsO_4 \cdot 2H_2O$ (5.5 mg, 0.0151 mmol, 0.002 equiv.), $(DHQ)_2PHAL$ (117.5 mg, 0.151 mmol, 0.02 equiv.), $H_3CSO_2NH_2$ (717 mg, 7.54 mmol, 1.0 equiv.), K_2CO_3 (3.13 g, 22.62 mmol, 3.0 equiv.), $NaHCO_3$ (1.90 g, 22.62 mmol, 3.0 equiv.) and $K_3Fe(CN)_6$ (7.45 g, 22.62 mmol, 3.0 equiv.). The solid reagents were dissolved in H_2O (38.0 mL) and *t*-BuOH (28.0 mL) and cooled to 0 °C. After 10 minutes at this temperature, the alkene **21** (1.05 g, 7.54 mmol, 1.0 equiv.) dissolved in *t*-BuOH (10.0 mL) was added slowly. The heterogeneous mixture was stirred vigorously at 0 °C for 1 week. The reaction was quenched with Na_2SO_3 (~5 g) and allowed to warm to room temperature with vigorous stirring for 30 minutes. The solution was transferred to a separatory funnel and extracted with EtOAc (8 x 20 mL). The combined organics were dried over $MgSO_4$, filtered, and concentrated *in vacuo*. The resulting crude residue (2.0 g) was used in the next reaction without further purification. A small portion of the crude reaction mixture was purified by column chromatography (SiO_2, 95:5 CH_2Cl_2/MeOH) for analytical purposes (the enantiomeric ratio was estimated to be 98:2 by Mosher's ester analysis; see below). The absolute configuration of diol **S5** was assumed based on the mnemonic rule for the facial selectivity of the Sharpless dihydroxylation reaction[2] and later confirmed with X-ray crystallography.

^{1}H NMR (600 MHz, $CDCl_3$) δ 4.12 (dd, J = 4.7, 2.3 Hz, 1H), 4.05 (t, J = 8.5 Hz, 1H), 3.87 (s, 3H), 3.19 (d, J = 4.9 Hz, 1H), 2.70 – 2.43 (m, 2H), 2.26 (d, J = 8.9 Hz, 1H), 2.05 – 1.85 (m, 2H).
^{13}C NMR (151 MHz, $CDCl_3$): δ 173.3, 119.5, 73.1, 70.6, 53.3, 29.7, 13.9.
HRMS (ES+) *m/z* calc'd for $C_7H_{11}NO_4$ $[M+Na]^+$: 196.0586, found 196.0581.
TLC: R_f = 0.43 (EtOAc, visualized by $KMnO_4$).
$[\alpha]^{21.6}_D$ –46.5 (c = 1.00, $CHCl_3$).

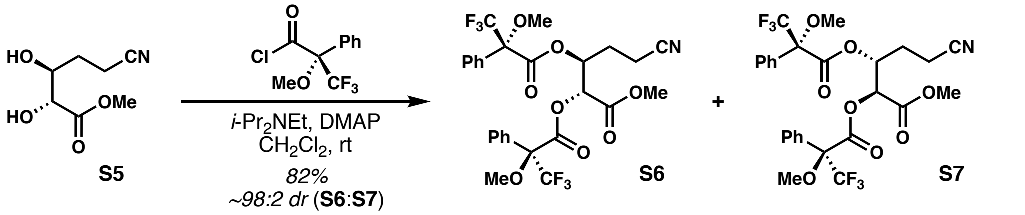

S5 S6 S7

This procedure was adapted from Vanderwal:[4] To a vial containing **S5** (10 mg, 0.058 mmol, 1.0 equiv.) and CH_2Cl_2 (0.8 mL) under Ar was added *i*-Pr_2NEt (50 μL, 0.290 mmol) and DMAP (7.0 mg, 0.058 mmol). Next, (*R*)-(–)-MTPA-Cl (43.8 mg, 0.144 mmol) in CH_2Cl_2 (0.2 mL) was added slowly to the above homogeneous solution. The vial was capped, and the reaction was maintained for 18 hours at room temperature. Upon completion, the reaction was quenched by dilution with CH_2Cl_2 (10 mL) and saturated aqueous $NaHCO_3$ (10 mL). The biphasic mixture was transferred to a separatory funnel and the layers were separated. The aqueous phase was extracted with CH_2Cl_2 (2 x 5 mL) and the combined organic extracts were dried over $MgSO_4$, filtered, and concentrated *in vacuo*. The resulting crude residue was purified by column chromatography (SiO_2, 3:1 hexanes:EtOAc) to afford predominantly **S6** as a colorless oil (30.0 mg, 82% yield).

Bis-(*S*)-MTPA ester (*2R,3S*)-S6:

^{1}**H NMR** (600 MHz, $CDCl_3$) δ 7.7 – 7.3 (m, 9H), 5.6 (dq, *J* = 9.9, 2.1 Hz, 1H), 5.3 (d, *J* = 2.1 Hz, 1H), 3.8 (s, 3H), 3.5 (s, 3H), 3.4 (d, *J* = 1.4 Hz, 3H), 2.2 – 2.0 (m, 2H), 2.0 – 1.9 (m, 2H).

^{13}**C NMR** (151 MHz, $CDCl_3$): δ 166.1, 166.1, 166.0, 131.9, 131.0, 130.1, 130.1, 128.7 (2C), 128.6 (2C), 127.9 (2C), 127.1 (2C), 123.1 (q, *J* = 289.0 Hz, 2C), 117.8, 85.1 (q, *J* = 28.2 Hz), 84.5 (q, *J* = 27.8 Hz), 73.7, 71.9, 55.9, 55.6, 53.4, 27.1, 13.2.

^{19}**F NMR** (376 MHz, $CDCl_3$): δ –71.37, –72.03.

TLC: R_f = 0.21 (3:1 hexanes:EtOAc, visualized by $KMnO_4$).

Figure S2. Comparison of the ^{19}F NMR spectra for the (S)-MTPA esters of **S6** and racemic **S6** derived from Sharpless dihydroxylation.

Bis-(*S*)-MTPA ester of rac-S6

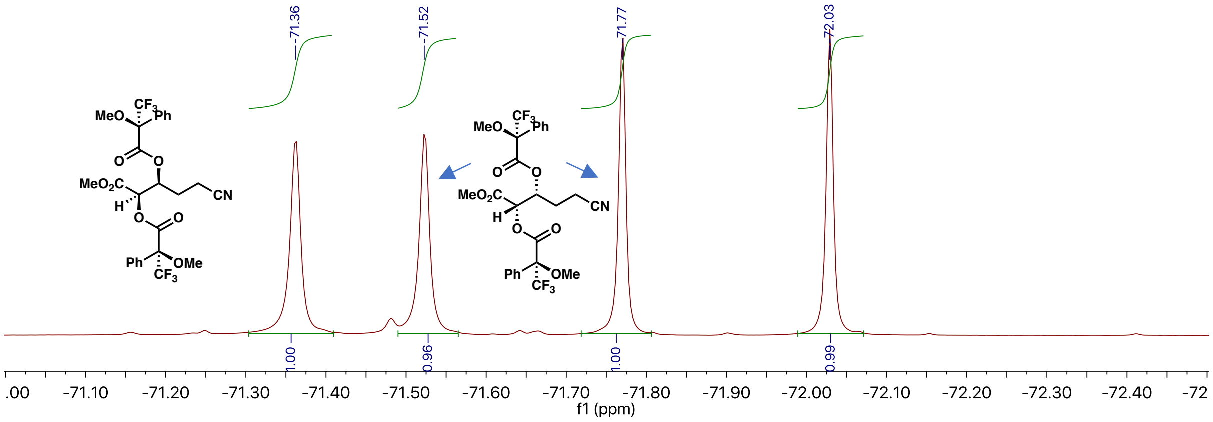

Bis-(*S*)-MTPA ester of predominantly S6 (ca. 98:2 dr)

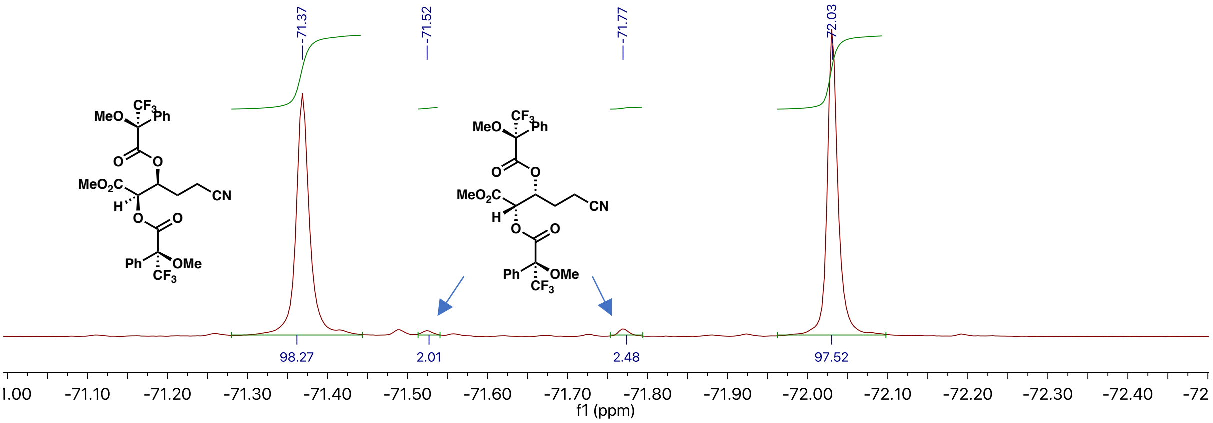

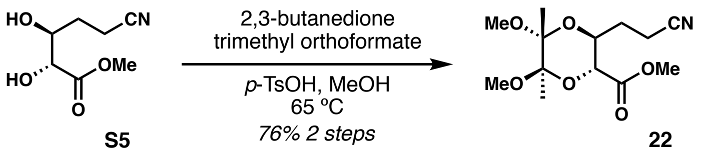

To a 50 mL round bottomed flask charged with crude diol **S5** (2.0 g) was added 2,3-butanedione (0.72 mL, 8.29 mmol), trimethyl orthoformate (4.1 mL, 37.7 mmol), and MeOH (9.4 mL, 0.8 M) open to air. *p*-TsOH•H_2O (143 mg, 0.754 mmol) was added in one portion, the flask was fitted with a reflux condenser and submerged in a preheated 80 °C oil bath. After 18 hours, the reaction mixture was cooled to room temperature and monitored by TLC which indicated moderate conversion. More *p*-TsOH (143 mg, 0.754 mmol) was added, and the mixture was again heated to reflux. After 5 hours, the reaction mixture was cooled to room temperature and K_2CO_3 (ca. 250 mg) was added and stirred vigorously for 5 minutes. The solution was diluted with CH_2Cl_2 (10 mL), filtered, and concentrated *in vacuo*. The resulting crude residue was purified by column chromatography (SiO_2, 3:1 hexanes:EtOAc) to afford **22** as a pale orange viscous oil (1.65 g, 76% yield over two steps).

^{1}H NMR (600 MHz, $CDCl_3$) δ 4.17 (d, J = 9.8 Hz, 1H), 4.01 (td, J = 10.1, 2.8 Hz, 1H), 3.80 (s, 3H), 3.31 (s, 6H), 2.56 (ddd, J = 7.8, 6.3, 2.8 Hz, 2H), 1.99 (dtd, J = 14.0, 8.2, 2.8 Hz, 1H), 1.83 (dddd, J = 14.0, 10.3, 7.0, 5.6 Hz, 1H), 1.37 (s, 3H), 1.32 (s, 3H).
^{13}C NMR (151 MHz, $CDCl_3$) δ 169.0, 119.3, 99.4, 99.0, 72.0, 66.6, 52.7, 48.6, 48.5, 26.9, 17.7, 17.5, 13.0.
HRMS (ES+) *m/z* calc'd for $C_{13}H_{21}NO_6$ $[M+Na]^+$: 310.1266, found 310.1266.
TLC: R_f = 0.22 (3:1 hexanes:EtOAc, visualized by CAM).
$[\alpha]^{21.9}_D$ –144.4 (c = 1.00, $CHCl_3$).

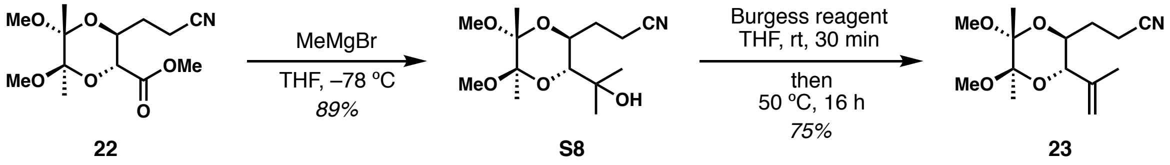

To a cooled (–78 °C) solution of **22** (1.55 g, 5.39 mmol, 1.0 equiv.) in THF (27.0 mL, 0.2 M) was added dropwise MeMgBr (10.8 mL, 32.34 mmol, 3.0 M in Et_2O, 6.0 equiv.) over 5 minutes. *Note: an excess of Grignard is necessary for obtaining high yields of **S8***. After 2 hours at –78 °C, TLC analysis indicated complete consumption of the starting material and the reaction was quenched slowly with AcOH (2.0 mL) at –78 °C. The cooling bath was removed, and the mixture was diluted with EtOAc (30 mL) and saturated aqueous NH_4Cl (30 mL). The biphasic solution was transferred to a separatory funnel and the layers were separated. The aqueous layer was extracted with EtOAc (2 x 15 mL) and the combined organic extracts were dried over $MgSO_4$ and purified over a short SiO_2 plug eluting with EtOAc (20 mL). The filtrate was concentrated *in vacuo* affording **S8** as a viscous yellow-orange oil (1.39 g, 89% yield) which was used without further purification.

^{1}H NMR (600 MHz, $CDCl_3$) δ 3.88 (t, J = 9.9 Hz, 1H), 3.35 (d, J = 9.5 Hz, 1H), 3.28 (s, 3H), 3.26 (s, 3H), 2.57 (dtd, J = 22.8, 16.7, 7.0 Hz, 2H), 2.23 (dt, J = 14.7, 7.8 Hz, 1H), 1.91 (br s, 1H), 1.81 (ddd, J = 12.2, 8.7, 5.6 Hz, 1H), 1.29 (s, 6H), 1.28 (s, 3H), 1.26 (s, 3H).
^{13}C NMR (150 MHz, $CDCl_3$) δ 120.0, 99.0, 98.6, 76.7, 71.7, 66.9, 48.4, 48.2, 28.1, 27.9, 25.4, 17.6, 17.6, 13.0.
HRMS (ES+) *m/z* calc'd for $C_{14}H_{25}NO_5$ $[M+Na]^+$: 310.1631, found 310.1626.
TLC: R_f = 0.10 (1:1 hexanes:Et_2O, visualized by CAM).
$[\alpha]^{22.5}_D$ –168.3 (c = 1.02, $CHCl_3$).

To a flame-dried 100 mL round-bottomed flask was added Burgess reagent (1.04 g, 4.35 mmol, 1.25 equiv.) followed by THF (10.0 mL). This flask was placed in a room temperature water bath before slow addition of **S8** (1.0 g, 3.48 mmol, 1.0 equiv) in THF (7.4 mL). After 15 minutes at room temperature, TLC analysis indicated consumption of the starting material and the presence of a more polar compound. The flask was submerged in a preheated 50 °C oil bath and allowed to react at this temperature overnight. After 20 hours, the flask was cooled to room temperature and concentrated *in vacuo*. The crude solids were dry loaded onto approximately 4 g of SiO_2 and purified by column chromatography (SiO_2, 7:1 then 4:1 hexanes:EtOAc) to afford **23** as a colorless crystalline solid (705 mg, 75% yield).

^{1}H NMR (500 MHz, $CDCl_3$) δ 5.02 – 4.99 (m, 2H), 3.92 (d, J = 9.7 Hz, 1H), 3.87 (td, J = 10.0, 2.8 Hz, 1H), 3.30 (s, 3H), 3.26 (s, 3H), 2.66 – 2.45 (m, 2H), 1.80 (d, J = 1.3 Hz, 3H), 1.78 – 1.71 (m, 1H), 1.70 – 1.62 (m, 1H), 1.31 (s, 3H), 1.30 (s, 3H).
^{13}C NMR (126 MHz, $CDCl_3$) δ 141.6, 119.6, 116.7, 99.1, 99.0, 76.6, 66.9, 48.3, 48.1, 26.9, 18.1, 17.8, 17.8, 13.1.
HRMS (ES+) *m/z* calc'd for $C_{14}H_{23}NO_4$ $[M+Na]^+$: 292.1525, found 292.1523.
TLC: R_f = 0.48 (1:1 hexanes:Et_2O, visualized by CAM).
$[\alpha]^{21.6}_D$ –212.5 (c = 1.01, $CHCl_3$).

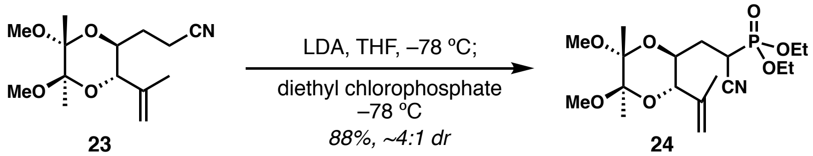

To a solution of *i*-Pr_2NH (0.69 mL, 4.90 mmol, 2.2 equiv) in THF (8.1 mL) at 0 °C was added dropwise *n*-BuLi (3.0 mL, 1.55 M, 4.66 mmol, 2.1 equiv.). The fresh LDA solution was maintained at this temperature for 30 minutes before cooling to –78 °C. After 10 minutes, a solution of **23** (600 mg, 2.22 mmol, 1.0 equiv.) in THF (3.0 mL) was added dropwise over 10 minutes to the LDA solution. The solution color gradually becomes yellow-orange at this stage of the reaction. After 30 minutes, diethyl chlorophosphate (0.39 mL, 2.66 mmol, 1.2 equiv.) was added neat dropwise over ~5 minutes. After 3 hours, the mixture was quenched at –78 °C with sat. aq. NH_4Cl (20 mL) and diluted with EtOAc

(20 mL). The layers were separated, and the aqueous phase was extracted with EtOAc (2 x 10 mL). The combined organic extracts were dried over $MgSO_4$ and concentrated *in vacuo*. The crude residue was purified by column chromatography (SiO_2, 1:1 then 1:2 hexanes:EtOAc) to afford **24** (1.10 g, 88% yield) as a pale yellow waxy solid upon standing in a –20 °C freezer, and (surprisingly!) as an inconsequential ~4:1 mixture of diastereomers. *Note: signals are reported for the major diastereomer only.*

^{1}H NMR (500 MHz, $CDCl_3$) δ 5.02 (s, 1H), 5.01 (s, 1H), 4.24 (ddq, J = 12.5, 8.7, 7.1 Hz, 4H), 4.06 – 3.99 (m, 1H), 3.92 (d, J = 9.6 Hz, 1H), 3.44 – 3.35 (m, 1H), 3.33 (s, 3H), 3.26 (s, 3H), 1.90 – 1.84 (m, 2H), 1.81 (s, 3H), 1.38 (t, J = 7.1 Hz, 6H), 1.30 (s, 3H), 1.29 (s, 3H).
^{13}C NMR (126 MHz, $CDCl_3$) δ 141.3, 117.0, 116.1 (d, J = 9.2 Hz), 99.1 (2C), 76.6, 65.8, 65.7, 64.3 (d, J = 6.8 Hz), 63.9 (d, J = 6.9 Hz), 48.6, 48.1, 28.6 (d, J = 3.6 Hz), 26.0 (d, J = 146.1 Hz), 18.0, 17.8 (2C), 16.5 (d, J = 5.8 Hz).
Note that these carbon assignments are tentative and they are assigned to the best of our abilities given the ^{31}P splitting and that this compound is a mixture of diastereomers.
HRMS (ES+) *m/z* calc'd for $C_{18}H_{32}NO_7P$ $[M+Na]^+$: 428.1814, found 428.1823.
TLC: R_f = 0.20 (1:1 hexanes:EtOAc, visualized by $KMnO_4$).
$[\alpha]^{22.2}_D$ –126.2 (c = 1.00, $CHCl_3$).

*Convergent Fragment Coupling to Diene **25**:*

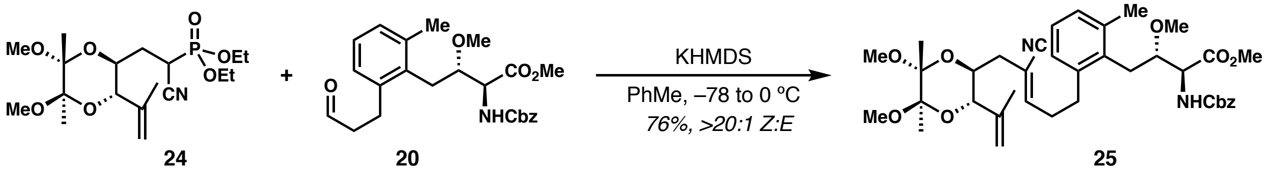

To flame-dried 20 mL vial containing a solution of cyanophosphonate (**24**) (46.6 mg, 0.115 mmol, 1.5 equiv.; azeotroped from PhMe 2 x 5 mL prior to use) in dry PhMe (0.5 mL) at –78 °C was added a freshly prepared solution of KHMDS (18.4 mg in 0.5 mL PhMe, 0.092 mmol, 1.2 equiv.) dropwise and the mixture was stirred at this temperature for 20 minutes. A solution of aldehyde **20** (32.8 mg, 0.0767 mmol, 1.0 equiv.; azeotroped from PhMe 2 x 5 mL prior to use) in PhMe (0.5 mL) was then added dropwise slowly down the side of the vial at –78 °C to the cyanophosphonate/KHMDS mixture. *Note: we found the addition down the side of the vessel to be important for obtaining high Z:E selectivities.* After 2 hours at –78 °C, the reaction was allowed to warm to 0 °C and stirred at this temperature for one hour. TLC analysis indicated consumption of the aldehyde **20** and formation of a less polar, UV active compound. The reaction was quenched at 0 °C with sat. aq. NH_4Cl (10 mL) and the biphasic mixture was transferred to a separatory funnel. The layers were separated, and the aqueous phase was extracted with CH_2Cl_2 (3 x 5 mL). The combined organic extracts were dried over $MgSO_4$, filtered, and concentrated *in vacuo*. The crude residue was purified by column chromatography (SiO_2, 85:15 then 7:3 hexanes:EtOAc) to afford **25** (39.6 mg, 76% yield, >20:1 *Z:E*) as a colorless viscous oil.

^{1}H NMR (600 MHz, $CDCl_3$) δ 7.45 – 7.30 (m, 5H), 7.10 – 6.99 (m, 3H), 6.30 (t, *J* = 7.6 Hz, 1H), 5.68 (d, *J* = 8.3 Hz, 1H), 5.26 – 5.10 (m, 2H), 5.01 (s, 1H), 5.00 (s, 1H), 4.71 (d, *J* = 8.3 Hz, 1H), 4.02 – 3.88 (m, 2H, *overlapping*), 3.82 (s, 3H), 3.69 (d, *J* = 11.0 Hz, 1H), 3.26 (s, 3H), 3.24 (s, 3H), 3.10 – 3.03 (m, 4H), 2.97 – 2.89 (m, 1H), 2.75 – 2.67 (m, 2H), 2.67 – 2.53 (m, 2H), 2.31 (s, 3H), 2.26 (d, *J* = 14.5 Hz, 1H), 2.19 (dd, *J* = 14.3, 9.1 Hz, 1H), 1.81 (s, 3H), 1.29 (s, 3H), 1.28 (s, 3H).
^{13}C NMR (150 MHz, $CDCl_3$) δ 170.9, 156.3, 149.8, 141.8, 139.7, 137.5, 136.4, 134.9, 128.9, 128.7 (2C), 128.3, 128.2 (2C), 127.2, 126.7, 117.5, 116.8, 111.5, 99.1, 99.0, 82.9, 76.5, 67.3 (2C), 59.6, 57.2, 52.7, 48.2, 48.1, 35.9, 33.0, 32.1, 31.1, 20.5, 18.1, 17.8 (2C).
HRMS (ES+) *m/z* calc'd for $C_{38}H_{50}N_2O_9$ $[M+Na]^+$: 701.3414, found 701.3420.
TLC: R_f = 0.75 (1:1 hexanes:EtOAc, visualized by CAM).
$[\alpha]^{21.7}_D$ –43.0 (c = 1.01, $CHCl_3$).

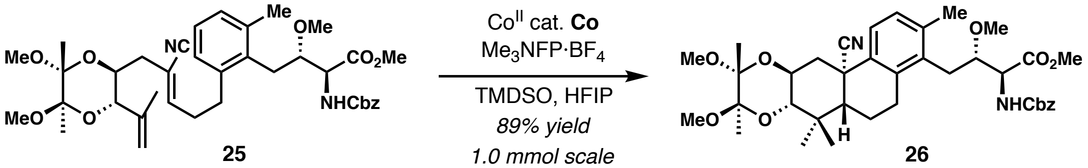

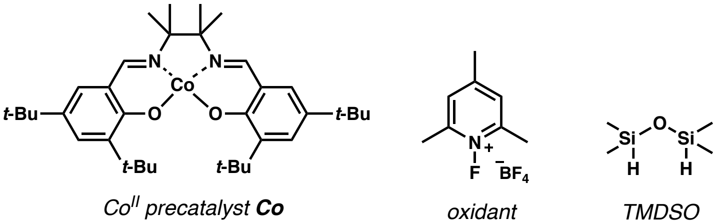

A 25 mL round bottomed flask was charged with cyclization substrate **25** (695 mg, 1.02 mmol, 1.0 equiv.), cobalt(II) precatalyst (62.0 mg, 0.102 mmol, 0.1 equiv.), and 1-fluoro-2,4,6-trimethylpyridinium tetrafluoroborate (694.5 mg, 3.06 mmol, 3.0 equiv.) open to air. The flask was capped with a rubber septum and the reagents were dissolved in HFIP (6.8 mL; a dark forest green solution forms immediately). A balloon with a syringe needle was used to bubble argon through solution for 20 minutes (a syringe needle was used as an outlet). The vial was sealed from the atmosphere and 1,1,3,3-tetramethyldisiloxane (0.54 mL, 3.06 mmol, 3.0 equiv.) was added dropwise slowly (approximately 1 drop per 3 seconds) over 15 minutes. The resulting solution gradually changed from dark green to a dark red color. After 4.5 hours, the reaction mixture was exposed to the atmosphere and volatiles were removed *in vacuo.* The resulting crude residue was purified by column chromatography (SiO_2, 7:3 hexanes:EtOAc) to afford **26** (619 mg, 89% yield) as a pale brown foamy solid in ca. 90% purity. Recrystallization from hot hexanes (60 ºC) provided a colorless powder. *Note: we believe that the inseparable impurity (ca. 10%) generated from this reaction is derived from the enantiopurity of aldehyde* ***20*** *(93:7 er). We found that using an aldehyde of lesser enantiopurity resulted in commensurate quantities of the inseparable impurity observed following this reaction.*

^{1}H NMR (600 MHz, $CDCl_3$) δ 7.55 – 7.31 (m, 5H), 7.23 (d, *J* = 8.2 Hz, 1H), 7.05 (d, *J* = 8.2 Hz, 1H), 5.57 (d, *J* = 8.6 Hz, 1H), 5.16 (s, 2H), 4.69 (dd, *J* = 8.4, 4.0 Hz, 1H), 4.19 (td,

J = 11.4, 10.1, 3.9 Hz, 1H), 3.82 (s, 3H), 3.69 (d, J = 10.5 Hz, 1H), 3.38 – 3.27 (m, 4H, *overlapping*), 3.25 – 3.21 (m, 4H, *overlapping*), 3.12 – 2.98 (m, 4H, *overlapping*), 2.90 (dd, J = 12.7, 4.1 Hz, 1H), 2.65 (d, J = 14.6 Hz, 2H), 2.30 (s, 3H), 2.09 (d, J = 8.4 Hz, 1H), 1.99 – 1.84 (m, 1H), 1.70 (t, J = 12.4 Hz, 1H), 1.36 (d, J = 11.9 Hz, 1H), 1.32 (s, 6H), 1.19 (s, 3H), 1.12 (s, 3H).
^{13}C NMR (150 MHz, $CDCl_3$) δ 170.8, 156.1, 137.3, 136.2, 136.1, 135.3, 135.1, 129.2, 128.7 (2C), 128.4, 128.3 (2C), 123.9, 123.0, 99.8, 99.3, 82.6, 77.3, 67.4, 64.8, 59.9, 57.3, 52.7, 49.4, 48.2, 47.8, 39.9, 39.5, 38.1, 31.3, 28.3, 26.9, 21.3, 20.2, 17.9 (2C), 15.1.
HRMS (ES+) *m/z* calc'd for $C_{38}H_{50}N_2O_9$ $[M+Na]^+$: 701.3414, found 701.3431.
TLC: R_f = 0.21 (7:3 hexanes:EtOAc, visualized by CAM).
$[\alpha]^{22.3}_D$ –52.2 (c = 1.00, $CHCl_3$).

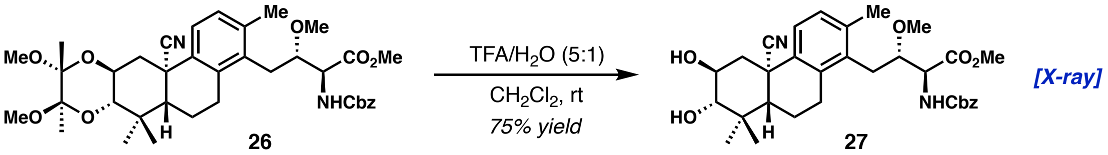

BDA protected **26** (110 mg, 0.162 mmol) was dissolved in CH_2Cl_2 (3.2 mL) open to air at 0 ºC, and then TFA/H_2O (5:1, 280 μL) was added slowly. After complete addition of the acid mixture, the ice bath was removed and allowed to warm to room temperature. After 25 minutes, TLC analysis indicated consumption of the starting material and the reaction was quenched with sat. aq. $NaHCO_3$ (10 mL) and diluted with CH_2Cl_2 (15 mL). The layers were separated, and the aqueous phase was extracted with CH_2Cl_2 (2 x 15 mL). The combined organic extracts were dried over $MgSO_4$, filtered, and concentrated *in vacuo*. The resulting crude residue was purified by column chromatography (SiO_2, 4:6 then 3:7 hexanes:EtOAc) to afford **27** (69.0 mg, 75% yield) as a colorless crystalline solid. *Note: the purity of diol **27** was estimated to be ~90% due to a presumed diastereomeric impurity derived from the convergent HWE coupling (see above).*

*X-ray quality crystals were grown by slow evaporation from a mixture of hexanes:CH_2Cl_2 (ca. 1:1) over several days. The setup for crystallization is as follows: diol **27** (ca. 20 mg) was transferred to a 1-dram vial and dissolved in a minimum amount of the solvent mixture (ca. 750 μL). This vial was then placed in a 20 mL scintillation vial containing hexanes (ca. 5-8 mL) which was capped. Slow diffusion of CH_2Cl_2 out of the inner vial over approximately 72 hours produced crystals of suitable quality for X-ray diffraction studies.*

^{1}H NMR (600 MHz, $CDCl_3$) δ 7.46 – 7.30 (m, 5H), 7.24 (d, J = 8.2 Hz, 1H), 7.07 (d, J = 8.0 Hz, 1H), 5.61 (d, J = 8.5 Hz, 1H), 5.16 (d, J = 1.8 Hz, 2H), 4.70 (dd, J = 8.7, 3.7 Hz, 1H), 4.11 (ddd, J = 11.6, 9.6, 4.1 Hz, 1H), 3.83 (s, 3H), 3.70 (d, J = 9.9 Hz, 1H), 3.34 – 3.27 (m, 1H), 3.22 – 2.93 (m, 6H, *overlapping*), 2.65 (d, J = 14.2 Hz, 3H, *overlapping*), 2.30 (s, 3H), 2.10 (dd, J = 12.9, 6.0 Hz, 1H), 1.92 (qd, J = 12.2, 5.6 Hz, 1H), 1.61 (t, J = 12.3 Hz, 1H), 1.38 (d, J = 11.4 Hz 1H), 1.15 (s, 5H, *overlapping*).

^{13}C NMR (150 MHz, $CDCl_3$) δ 170.9, 156.2, 137.4, 136.2 (2C), 135.2, 135.1, 129.2, 128.7 (2C), 128.4, 128.3 (2C), 124.0, 123.1, 82.5, 82.0, 68.7, 67.4, 59.9, 57.2, 52.7, 49.1, 42.0, 39.6, 39.4, 31.2, 28.4, 27.5, 21.6, 20.2, 14.8.
HRMS (ES+) *m/z* calc'd for $C_{32}H_{40}N_2O_7$ $[M+Na]^+$: 587.2733, found 587.2742.
TLC: R_f = 0.18 (1:1 hexanes:EtOAc, visualized by CAM).
$[\alpha]^{22.3}_D$ –12.2 (c = 1.05, $CHCl_3$).

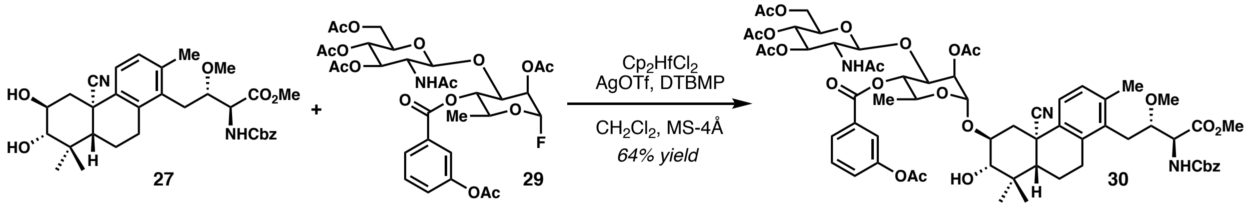

The procedure for glycosylation was modified from Yoshimura:[5] Cp_2HfCl_2 (48.6 mg, 0.128 mmol), AgOTf (65.8 mg, 0.256 mmol), and 4Å MS (70 mg) were combined in a flame-dried 10 mL round bottomed flask under Ar. The flask was charged with CH_2Cl_2 (1.7 mL) and stirred vigorously for 15 minutes at room temperature. Next, the flask was cooled to –78 ºC and, after 10 minutes, a solution of **27** (18.1 mg, 0.032 mmol), **29** (56.0 mg, 0.0801 mmol) and DTBMP (3.3 mg, 0.016 mmol) in CH_2Cl_2 (1.0 mL) was added over 5 minutes to the promoter solution followed by a rinse with CH_2Cl_2 (0.5 mL). Note: **27** and **29** were combined and azeotroped from PhMe (2 x 3 mL) prior to use. After 10 minutes the cooling bath was removed, and the reaction was allowed to warm to room temperature. After 35 minutes, the reaction was quenched with sat. aq. $NaHCO_3$ (5 mL) and then filtered through a celite pad eluting with EtOAc (2 x 5 mL). The filtrate was transferred to a separatory funnel and the layers were separated. The aqueous phase was extracted with EtOAc (2 x 10 mL) and the combined organic extracts were dried over $MgSO_4$, filtered, and concentrated *in vacuo*. The resulting crude residue was purified by column chromatography (pH 7 SiO_2, 1:1 then 3:7 hexanes:EtOAc) to afford **30** (25.4 mg, 64%) as a colorless film.

^{1}H NMR (600 MHz, CD_3OD) δ 7.97 (dt, *J* = 7.8, 1.4 Hz, 1H), 7.81 (t, *J* = 2.0 Hz, 1H), 7.64 (d, *J* = 8.7 Hz, 1H), 7.56 (t, *J* = 7.9 Hz, 1H), 7.42 – 7.37 (m, 3H), 7.38 – 7.32 (m, 2H), 7.33 – 7.29 (m, 1H), 7.24 (d, *J* = 8.2 Hz, 1H), 7.09 (d, *J* = 8.2 Hz, 1H), 5.46 (dd, *J* = 3.5, 1.8 Hz, 1H), 5.28 (t, *J* = 9.9 Hz, 1H), 5.22 (d, *J* = 1.9 Hz, 1H), 5.22 – 5.08 (m, 3H), 4.94 (t, *J* = 9.7 Hz, 1H), 4.89 (d, *J* = 8.3 Hz, 1H), 4.68 (d, *J* = 5.0 Hz, 1H), 4.29 (dd, *J* = 9.8, 3.5 Hz, 1H), 4.22 (dd, *J* = 12.2, 2.5 Hz, 1H), 4.19 – 4.13 (m, 1H), 4.13 (dd, *J* = 12.2, 3.6 Hz, 1H), 4.04 (ddd, *J* = 11.8, 9.8, 4.2 Hz, 1H), 3.79 (s, 3H), 3.78 – 3.70 (m, 2H), 3.61 (dd, *J* = 10.5, 8.3 Hz, 1H), 3.26 (d, *J* = 9.8 Hz, 1H), 3.26 – 3.17 (m, 1H), 3.13 (dd, *J* = 12.9, 4.3 Hz, 1H), 3.11 – 3.03 (m, 1H), 3.03 (s, 3H), 2.73 (ddd, *J* = 17.7, 12.1, 6.8 Hz, 1H), 2.64 (d, *J* = 13.5 Hz, 1H), 2.35 – 2.27 (m, 6H, *overlapping*), 2.15 (s, 3H), 2.14 – 2.10 (m, 1H), 2.09 (s, 3H), 1.97 (d, *J* = 2.4 Hz, 4H, *overlapping*), 1.90 (s, 3H), 1.87 – 1.76 (m, 1H), 1.71 (t, *J* = 12.3 Hz, 1H), 1.45 (d, *J* = 12.2 Hz, 1H), 1.25 (d, *J* = 6.0 Hz, 3H), 1.23 (s, 3H), 1.20 – 1.16 (m, 1H), 1.16 (s, 3H), 1.11 (s, 3H).
^{13}C NMR (150 MHz, CD_3OD) δ 173.2, 173.0, 172.4, 171.8, 171.7, 171.1 (2C), 165.9, 158.7, 152.4, 138.8, 138.2, 137.6, 136.5, 136.4, 132.4, 131.0, 130.0, 129.5, 129.1, 128.9, 128.4, 128.1, 124.7, 124.4, 124.3, 102.3, 100.5, 83.0, 81.4, 78.2, 77.2, 74.1, 73.7, 73.1,

72.6, 69.8, 68.1, 67.8, 62.6, 59.4, 58.1, 55.9, 52.8, 49.8, 42.5, 40.9, 40.8, 37.6, 31.8, 29.4, 27.8, 25.6, 22.7, 22.3, 21.0, 20.9 (2C), 20.6, 20.5 (2C), 18.0, 15.3.
HRMS (ES+) *m/z* calc'd for $C_{63}H_{77}N_3O_{23}$ $[M+Na]^+$: 1266.4845, found 1266.4863.
TLC: R_f = 0.46 (EtOAc, visualized by UV and $KMnO_4$).
$[\alpha]^{21.5}_D$ –11.2 (c = 1.03, CH_3OH).

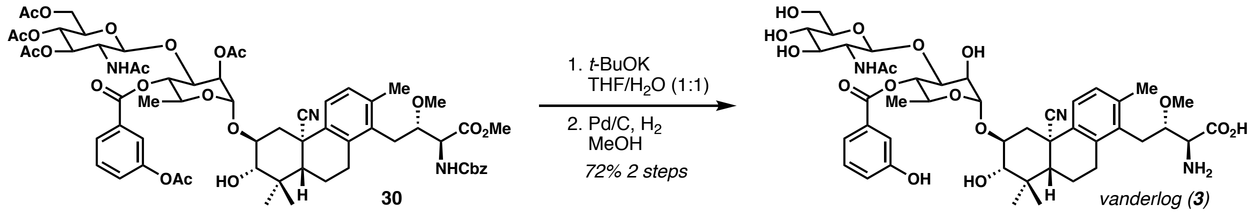

The procedure for glycoside hydrolysis was adapted from Stegmann:[6] Protected glycoside **30** (14.0 mg, 0.0112 mmol) was dissolved in a solution of 0.1 M *t*-BuOK in 1:1 THF/H_2O (1.1 mL, 0.11 mmol) at room temperature and open to air in a 2-dram vial. A pale-yellow solution formed immediately, and the vial was capped. After 5 hours, the reaction mixture was concentrated to remove THF, and then H_2O (1 mL) and EtOAc (5 mL) were added. The layers were separated, and the organic phase was discarded. The aqueous phase was acidified to pH ~2 with 0.5 M HCl (~1 mL) and then extracted with EtOAc (4 x 10 mL). The combined organic extracts were dried over $MgSO_4$, filtered, and concentrated *in vacuo*. The resulting crude residue was dissolved in MeOH (1.0 mL) and treated with 10% Pd/C (5 mg) in one portion. The heterogeneous solution was sparged with H_2 gas for a minute at room temperature then the bleeding needle was removed and allowed to react overnight. After 16 hours, the reaction mixture was filtered over celite and rinsed with MeOH (~5 mL). The filtrate was concentrated, and the material was purified on Sephadex LH-20 resin eluting with MeOH which afforded brasilicardin analog **3** (7.2 mg, 72% over 2 steps) as a colorless foamy solid.

^{1}H NMR (600 MHz, CD_3OD) δ 7.55 (d, *J* = 7.7 Hz, 1H), 7.48 (s, 1H), 7.33 (t, *J* = 7.8 Hz, 1H), 7.24 (d, *J* = 8.4 Hz, 1H), 7.09 (d, *J* = 8.1 Hz, 1H), 7.05 (dd, *J* = 8.2, 2.6 Hz, 1H), 5.33 (t, *J* = 9.8 Hz, 1H), 5.19 (s, 1H), 4.54 (d, *J* = 8.5 Hz, 1H), 4.39 (s, 1H), 4.15 – 4.06 (m, 2H), 4.04 – 3.97 (m, 2H), 3.92 (d, *J* = 11.0 Hz, 1H), 3.88 (d, *J* = 11.6 Hz, 1H), 3.70 (dd, *J* = 11.9, 5.3 Hz, 1H), 3.56 (q, *J* = 9.0, 8.6 Hz, 1H), 3.41 – 3.31 (m, 2H), 3.29 – 3.17 (m, 2H), 3.13 (s, 3H), 3.06 – 3.00 (m, 1H), 2.84 – 2.73 (m, 1H), 2.69 (d, *J* = 14.0 Hz, 1H), 2.35 (s, 3H), 2.27 (t, *J* = 7.5 Hz, 1H), 2.21 – 2.12 (m, 1H), 1.89 – 1.74 (m, 1H), 1.70 (t, *J* = 12.2 Hz, 1H), 1.63 – 1.56 (m, 2H), 1.53 – 1.42 (s, 3H), 1.23 (d, *J* = 6.4 Hz, 3H), 1.15 (s, 3H), 1.12 (s, 3H). *Note that only C–Hs are accounted for in the tabulated data above.*
^{13}C NMR (150 MHz, CD_3OD) δ 174.0, 167.0, 159.0, 139.1, 137.2, 136.5, 136.5, 132.4, 130.9, 130.0, 129.4, 124.8, 124.4, 122.0, 121.5, 117.5, 104.1, 103.3, 81.5, 81.3, 79.8, 78.3, 77.8, 75.2, 74.0, 72.0, 71.7, 68.4, 62.5, 58.5, 57.5, 42.7, 40.9, 40.8, 33.1, 30.5, 27.8, 23.7, 22.6, 20.6, 18.0, 15.3, 14.4.
HRMS (ES+) *m/z* calc'd for $C_{44}H_{59}N_3O_{16}$ $[M+Na]^+$: 908.3793, found 908.3818.
TLC: R_f = 0.13 (MeOH, visualized by CAM).
$[\alpha]^{21.1}_D$ –44.6 (c = 1.01, CH_3OH).

Method for Computing Lowest Energy Conformers

All calculations were performed using Spartan 18[7] on the aglycones of structures **1**, **2**, and **3** in the gas phase and plotted using Cylview[8] and PyMOL. A general method for computing the lowest energy conformer for each compound is as follows: Lowest energy conformers were obtained via a conformer search using Molecular Mechanics MMFF which were then subjected to an equilibrium geometry calculation. The remaining conformers were refined with ωB97X-D/6-31G* and only the lowest energy conformer was plotted against the other aglycones structures for a rough approximation of structural similarity.

The above procedure generated many conformers for each structure, mostly varying in the conformation of the amino acid side chain. However, we only intended to compare the structural rigidity of the terpene-like cores and the vectors at which the disaccharide and the amino acid motifs would be presented from those core scaffolds. We did not rigorously model the amino acid side chain because of its expected conformational mobility and because the energies of different conformers would be expected to be impacted by solvation and of course by macromolecule binding. The intent was not to rigorously compare the entire structures, but rather to see how good an approximation of the natural product core scaffold our designed tricyclic core should be.

Jung's brasilogue aglycone

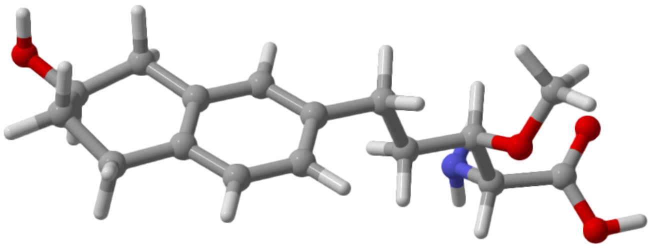

44

C	-1.125732	3.862035	0.080157
C	0.821608	2.941761	1.431511
C	0.066941	5.352423	1.693354
C	0.832435	4.179122	2.306512
C	-1.300117	4.892299	1.195018
C	-0.121776	2.776520	0.410692
O	-2.121363	5.969664	0.784151
C	-0.115474	1.598859	-0.340929
C	0.807431	0.581972	-0.117388
C	1.748602	0.761289	0.899784

C	1.749875	1.921932	1.658626
C	0.763558	-0.701406	-0.909699
C	-0.048425	-1.787204	-0.193016
C	-0.087326	-3.100021	-0.963282
C	-0.989438	-4.138496	-0.273932
C	-0.747205	-5.487495	-0.939611
N	-2.381980	-3.721278	-0.370396
O	1.216778	-3.652171	-1.062519
C	1.715577	-3.747985	-2.378022
O	-1.036144	-5.728689	-2.091301
O	-0.220982	-6.405587	-0.116505
H	-0.786411	4.394858	-0.822624
H	-2.097832	3.423611	-0.170368
H	0.633559	5.766917	0.846084
H	-0.061786	6.155423	2.426230
H	1.866335	4.472379	2.521626
H	0.380167	3.929395	3.276722
H	-1.841558	4.431296	2.029711
H	-1.667846	6.415896	0.057453
H	-0.857076	1.476947	-1.129542
H	2.484473	-0.015721	1.094042
H	2.489352	2.047136	2.446870
H	1.782151	-1.072501	-1.071394
H	0.327302	-0.513419	-1.899413
H	0.394190	-1.976017	0.793470
H	-1.076240	-1.446707	-0.029247
H	-0.495720	-2.928224	-1.971334
H	-0.708286	-4.207481	0.780587
H	-2.965524	-4.284784	0.240536
H	-2.707088	-3.880737	-1.321718
H	2.710483	-4.193505	-2.305511
H	1.804368	-2.759255	-2.850885
H	1.077980	-4.386811	-3.003831
H	-0.111841	-7.216243	-0.640667

Brasilogue aglycone

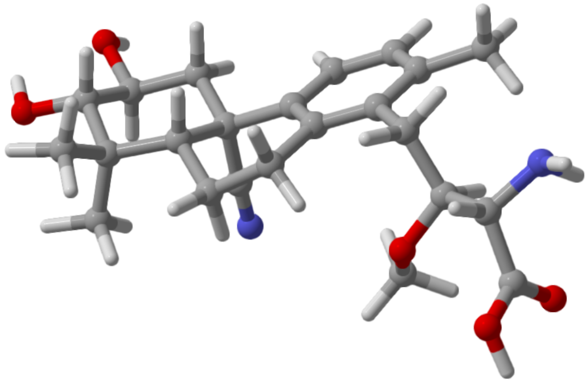

62

C	0.362591	3.565322	-0.104852
C	-0.358618	1.779040	1.541717
C	-0.377951	4.197435	2.207072
C	-1.036256	2.828443	2.469162
C	-0.389140	4.588472	0.736273
C	-0.247162	2.151881	0.033288
C	0.583214	1.082367	-0.711453
C	0.689506	-0.232544	-0.230820
C	0.043661	-0.647869	1.078289
C	-0.939607	0.371405	1.640955
C	1.426317	-1.183453	-0.965845
C	2.062734	-0.823168	-2.164139
C	1.928131	0.482931	-2.628629
C	1.192408	1.416685	-1.920582
C	-2.562155	2.905977	2.300586
C	-0.738064	2.464003	3.935552
H	0.691610	1.725732	1.869936
C	-1.569314	2.154302	-0.631246
C	2.902203	-1.804530	-2.947039
C	1.494595	-2.628491	-0.519988
C	0.401240	-3.485882	-1.171796
C	0.676113	-4.986698	-0.979648
O	-0.876229	-3.211932	-0.626105
N	1.837684	-5.364076	-1.773743
C	-0.580494	-5.737450	-1.402614
O	-0.967183	-5.790690	-2.548785
O	-1.201913	-6.359812	-0.392268
C	-1.736899	-2.476669	-1.475370
N	-2.579832	2.153551	-1.198532
O	0.237328	5.866597	0.673674
O	-1.017992	5.188445	2.977219
H	1.407005	3.525955	0.224915

H	0.360562	3.893084	-1.148613
H	0.685075	4.121875	2.503569
H	-1.431200	4.670494	0.397155
H	0.842107	-0.811299	1.817603
H	-0.459879	-1.608286	0.948303
H	-1.164675	0.111277	2.678853
H	-1.888710	0.324176	1.093028
H	2.403991	0.772542	-3.561756
H	1.093414	2.416571	-2.328010
H	-2.966119	3.643690	2.997948
H	-2.875152	3.189976	1.293841
H	-3.019824	1.936961	2.524645
H	-1.275349	1.563580	4.248310
H	0.334061	2.295087	4.094463
H	-1.051818	3.284462	4.586274
H	3.812673	-2.071132	-2.396818
H	2.378315	-2.743428	-3.151296
H	3.207967	-1.373226	-3.904184
H	2.457875	-3.068935	-0.791183
H	1.397823	-2.725012	0.565031
H	0.381473	-3.299745	-2.255715
H	0.877839	-5.184889	0.076979
H	1.553499	-5.435484	-2.748750
H	2.176444	-6.280788	-1.497602
H	-1.989636	-6.784021	-0.770851
H	-1.319064	-1.493898	-1.726558
H	-1.941723	-3.034058	-2.399260
H	-2.670443	-2.335121	-0.926925
H	0.001269	6.281965	-0.162791
H	-0.668326	6.028302	2.649128

Brasilicardin aglycone

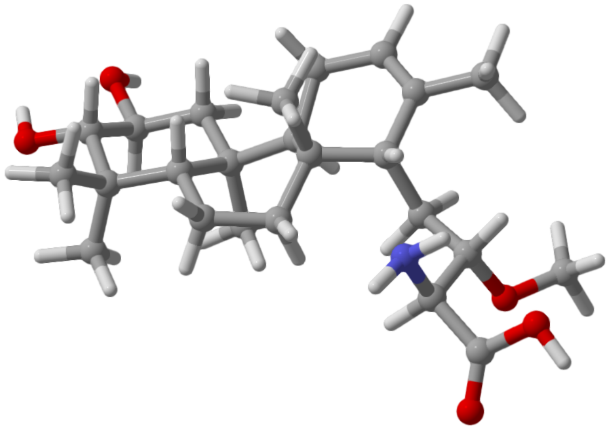

71

C	-3.427504	0.091483	-0.304728
C	-1.818805	-0.002768	1.620222
C	-4.283722	0.013804	2.051398
C	-2.934699	-0.473359	2.611769
C	-4.519666	-0.435503	0.616609
C	-2.004934	-0.340711	0.112564
C	-0.909690	0.423020	-0.743811
C	0.389352	0.843390	0.009655
C	0.707635	-0.213493	1.071971
C	-0.399046	-0.322064	2.135168
C	1.502692	0.976181	-1.072453
C	1.044903	1.884481	-2.203179
C	-0.244845	2.152378	-2.424501
C	-1.379811	1.604678	-1.600020
C	-1.846783	-1.845334	-0.196653
H	-0.570049	-0.322688	-1.474586
C	0.328932	2.219734	0.714537
C	2.136290	2.453400	-3.069722
C	2.025721	-0.358635	-1.670611
H	-1.924119	1.089568	1.645201
C	-2.994917	-1.983719	2.904178
C	-2.713940	0.235209	3.963103
C	3.539321	-0.574095	-1.589831
O	3.898404	-1.694715	-2.382128
C	4.224388	-1.373114	-3.710125
C	4.076370	-0.888681	-0.183319
C	5.451750	-1.537714	-0.301551
N	4.109895	0.306053	0.655441

O	5.784230	-2.591226	0.179639
O	6.324630	-0.728339	-0.947727
O	-5.338063	-0.428555	2.879533
O	-5.798282	0.093907	0.265843
H	-3.515440	1.183368	-0.291155
H	-3.632843	-0.230259	-1.335236
H	-4.262396	1.119230	2.034411
H	-4.569877	-1.532272	0.593287
H	1.666148	0.023784	1.546701
H	0.840368	-1.189797	0.596356
H	-0.174528	0.362494	2.959711
H	-0.366376	-1.328961	2.564487
H	2.351243	1.461514	-0.576150
H	-0.516027	2.814401	-3.246495
H	-1.799802	2.407529	-0.975700
H	-2.186591	1.297338	-2.275306
H	-1.888943	-2.005845	-1.280416
H	-0.890103	-2.240306	0.156240
H	-2.630540	-2.458370	0.249271
H	-0.487573	2.312062	1.432745
H	1.265297	2.368564	1.264993
H	0.236140	3.041227	0.000094
H	1.735302	3.086948	-3.866502
H	2.729027	1.657627	-3.541118
H	2.834660	3.053251	-2.471766
H	1.543811	-1.226880	-1.211778
H	1.753396	-0.410709	-2.731123
H	-3.692478	-2.160916	3.726529
H	-3.344025	-2.580703	2.060432
H	-2.016694	-2.370636	3.204266
H	-2.549552	1.311364	3.826512
H	-3.593670	0.103794	4.599001
H	-1.848197	-0.176274	4.491829
H	4.066046	0.321920	-1.957904
H	3.394109	-0.873151	-4.230955
H	5.110912	-0.723433	-3.753312
H	4.442424	-2.312449	-4.222558
H	3.428269	-1.629618	0.292718
H	4.831729	0.939939	0.321529
H	4.348069	0.055467	1.610639
H	7.174616	-1.196181	-0.949720
H	-6.075072	-0.314456	-0.561555
H	-6.146478	-0.233177	2.385112

PyMOL overlays of the aglycones for structures 1, 2, and 3

The structures of the aglycones were plotted in PyMOL by pair fitting the carbon atoms of the A ring to each respective compound. Note that these overlays are based on electronic structure calculations that do not take solvation into account.

Figure S3. Overlay of the aglycones for brasilicardin A (**1**) and proposed brasilogue (**3**)

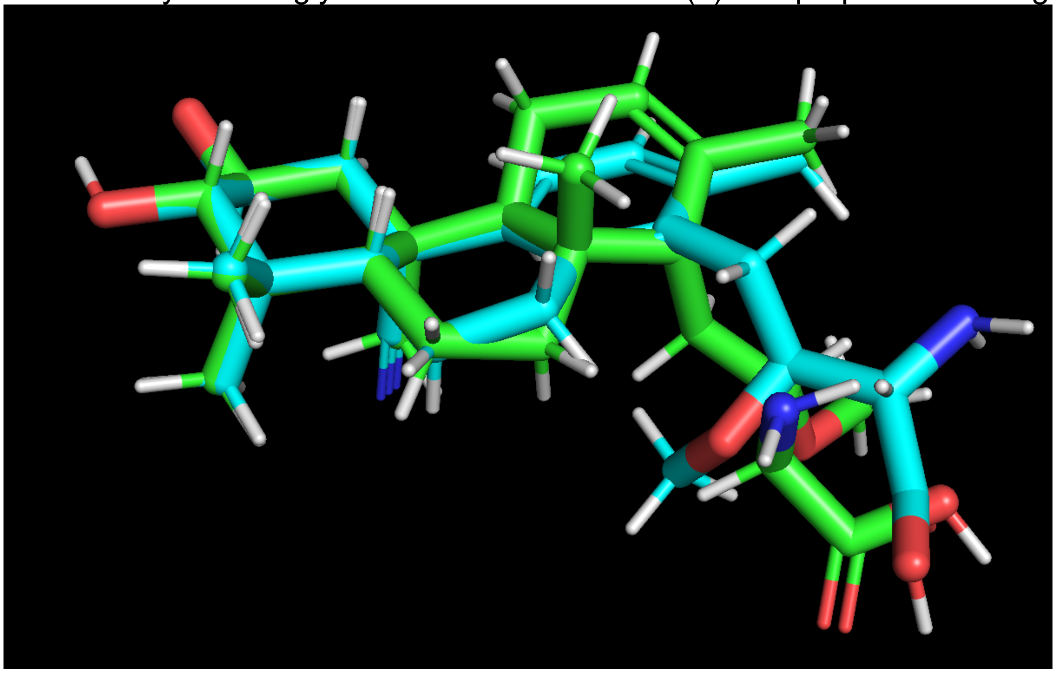

Figure S4. Overlay of the aglycones for brasilicardin A (**1**) and Jung's brasilog (**2**)

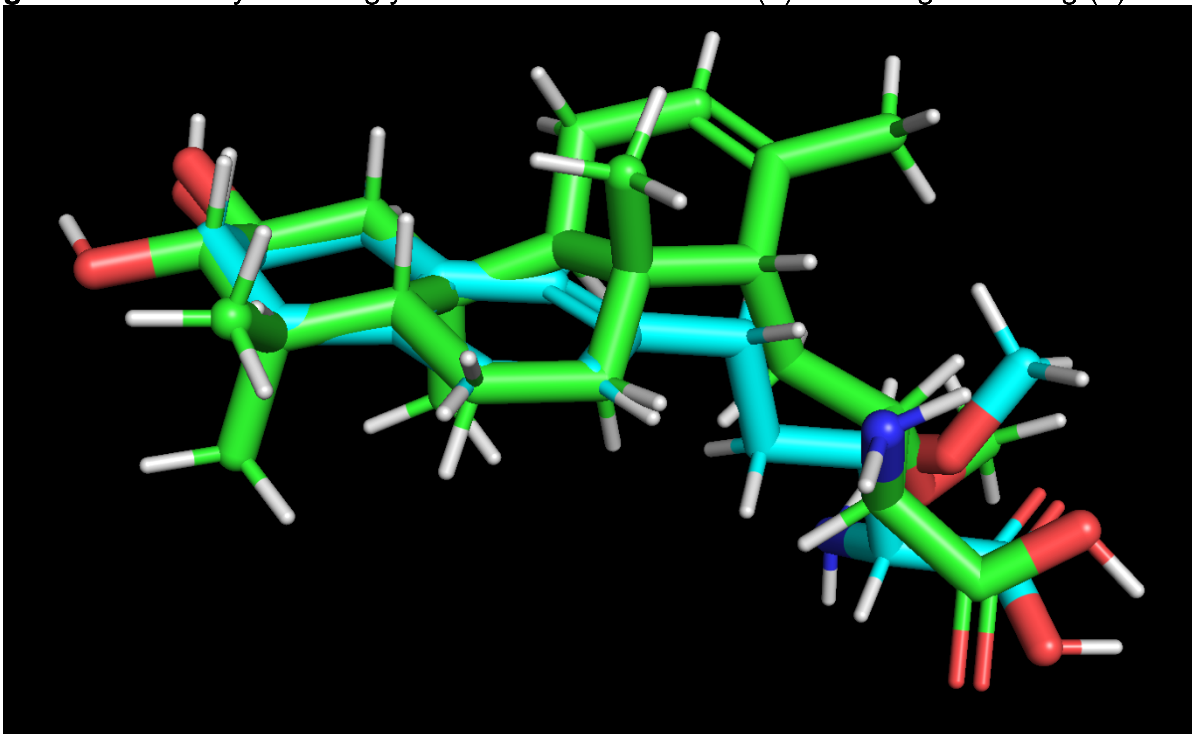

Immunosuppression Assay Materials and Methods

Cell Culture and Cell cycle

A mouse T cell lymphoma cell line, CTLL-2, was obtained from ATCC and maintained in RPMI 1640 medium supplemented with 10% fetal bovine serum, 2 mM L-glutamine; additional 1mM sodium pyruvate and 10% T-STIM with Con A (Becton Dickinson) at 37 ºC with 5% CO_2. Cells were treated for 24h with rapamycin (100 nM) or with the brasilicardin analog **3** (30 and 100 nM). Negative control cells were treated with the vehicle alone (0.1% DMSO, shown at NT (not treated) in the figure. After 24h cells were harvested and washed in PBS with 5 mM EDTA, then fixed in cold ethanol for 30min at room temperature. After 30 min, cells were spined down, washed and treated with ribonuclease for 30min at 37 ºC. Cells were stained with propidium iodide, and 10,000 cellular events were collected by flow cytometry using Attune NxT (Thermo Fisher). Cell cycle was quantified using FlowJo software v10.8.1 (FlowJo LLC).

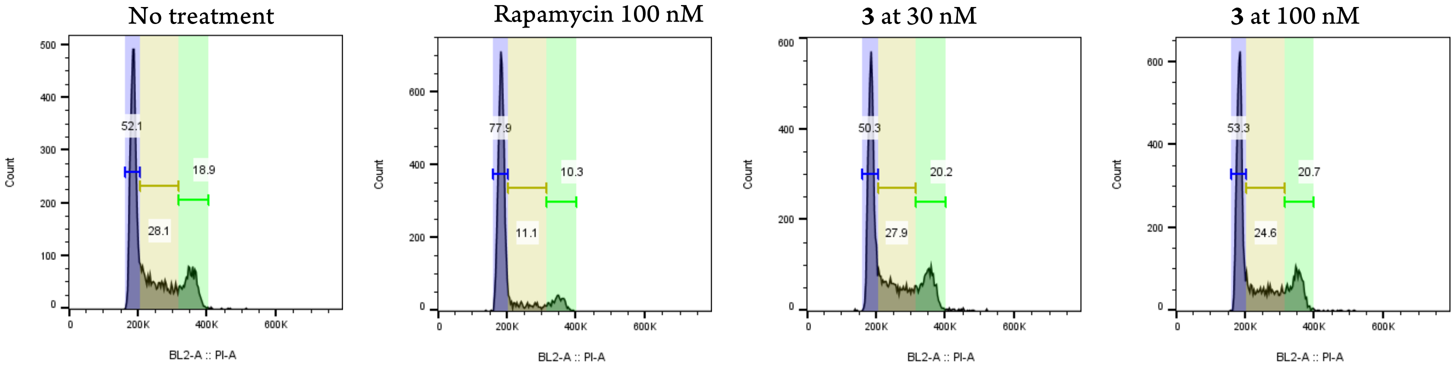

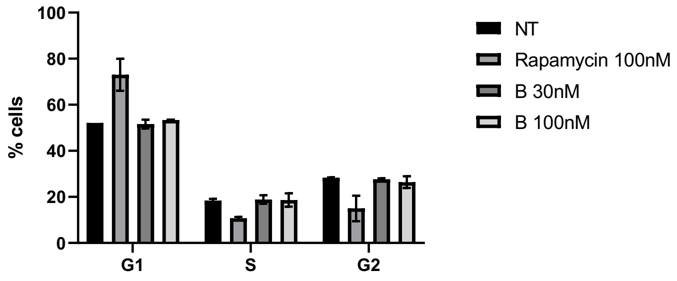

Western Blotting Analysis

Total cell lysates were prepared using RIPA buffer. Protein concentration was measured with Bradford assay (Bio-Rad Laboratories, Hercules, CA). Equal amounts of protein (20 µg) were heat-denatured in sample buffer (Bio-Rad Laboratories), resolved by SDS-PAGE using Novex 4-20% Tris-Glycine MiniProtein Gels (Thermo Fisher Scientific, Waltham, MA), and transferred to nitrocellulose membranes (Bio-Rad Laboratories).The filters were blocked in 5% BSA for 1h at room temperature and then incubated overnight at 4 ºC with specific antibodies for vinculin (13901 1:1000), pS6 S240/244 (5364 1:1000), p4E-BP1 Thr37/46 (2855 1:1000) and total 4E-BP1 (9644 1:1000) purchased from Cell Signaling Technologies (Danvers, MA). Goat anti rabbit-peroxidase conjugated IgG (W4011 1:5000, Promega Madison, WI) was used as secondary antibody.

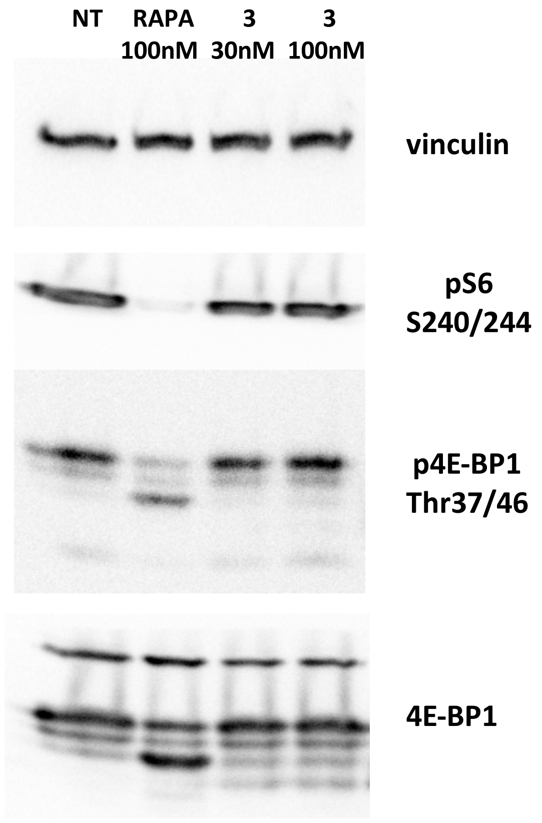

II. References

1. Mei, T.-S.; Wang, D.-H.; Yu, J.-Q. Expedient Drug Synthesis and Diversification via Ortho-C−H Iodination Using Recyclable PdI_2 as the Precatalyst. *Org. Lett.* **2010**, *12*, 3140–3143.
2. Kolb, H. C.; VanNieuwenhze, M. S.; Sharpless, K. B. Catalytic Asymmetric Dihydroxylation. *Chem. Rev.* **1994**, *94*, 2483–2547.
3. Kelly, A. M.; Pérez-Fuertes, Y.; Fossey, J. S.; Yeste, S. L.; Bull, S. D.; James, T. D. Simple Protocols for NMR Analysis of the Enantiomeric Purity of Chiral Diols. *Nat. Protoc.* **2008**, *3*, 215–219.
4. Johnson, L. K.; Niman, S. W.; Vrubliauskas, D.; Vanderwal, C. D. Stereocontrolled Synthesis and Structural Revision of Plebeianiol A. *Org. Lett.* **2021**, *23*, 9569–9573.
5. Yoshimura, F.; Itoh, R.; Torizuka, M.; Mori, G.; Tanino, K. Asymmetric Total Synthesis of Brasilicardins. *Angew. Chem. Int. Ed.* **2018**, *57*, 17161–17167.
6. Botas, A.; Eitel, M.; Schwarz, P. N.; Buchmann, A.; Costales, P.; Núñez, L. E.; Cortés, J.; Morís, F.; Krawiec, M.; Wolański, M.; Gust, B.; Rodriguez, M.; Fischer, W.-N.; Jandeleit, B.; Zakrzewska-Czerwińska, J.; Wohlleben, W.; Stegmann, E.; Koch, P.; Méndez, C.; Gross, H. Genetic Engineering in Combination with Semi-Synthesis Leads to a New Route for Gram-Scale Production of the Immunosuppressive Natural Product Brasilicardin A. *Angew. Chem. Int. Ed.* **2021**, *60*, 13536–13541.
7. (a) Spartan 18, Wavefunction Inc., Irvine CA. (b) Shao, Y.; Gan, Z.; Epifanovsky, E.; Gilbert, A. T. B.; Wormit, M.; Kussmann, J.; Lange, A. W.; Behn, A.; Deng, J.; Feng, X.; Ghosh, D.; Goldey, M.; Horn, P. R.; Jacobson, L. D.; Kaliman, I.; Khaliullin, R. Z.; Kuś, T.; Landau, A.; Liu, J.; Proynov, E. I.; Rhee, Y. M.; Richard, R. M.; Rohrdanz, M. A.; Steele, R. P.; Sundstrom, E. J.; Woodcock, H. L., III; Zimmerman, P. M.; Zuev, D.; Albrecht, B.; Alguire, E.; Austin, B.; Beran, G. J. O.; Bernard, Y. A.; Berquist, E.; Brandhorst, K.; Bravaya, K. B.; Brown, S. T.; Casanova, D.; Chang, C.-M.; Chen, Y.; Chien, S. H.; Closser, K. D.; Crittenden, D. L.; Diedenhofen, M.; DiStasio, R. A., Jr.; Do, H.; Dutoi, A. D.; Edgar, R. G.; Fatehi, S.; Fusti-Molnar, L.; Ghysels, A.; Golubeva-Zadorozhnaya, A.; Gomes, J.; Hanson-Heine, M. W. D.; Harbach, P. H. P.; Hauser, A. W.; Hohenstein, E. G.; Holden, Z. C.; Jagau, T.-C.; Ji, H.; Kaduk, B.; Khistyaev, K.; Kim, J.; Kim, J.; King, R. A.; Klunzinger, P.; Kosenkov, D.; Kowalczyk, T.; Krauter, C. M.; Lao, K. U.; Laurent, A. D.; Lawler, K. V.; Levchenko, S. V.; Lin, C. Y.; Liu, F.; Livshits, E.; Lochan, R. C.; Luenser, A.; Manohar, P.; Manzer, S. F.; Mao, S.-P.; Mardirossian, N.; Marenich, A. V.; Maurer, S. A.; Mayhall, N. J.; Neuscamman, E.; Oana, C. M.; Olivares-Amaya, R.; O'Neill, D. P.; Parkhill, J. A.; Perrine, T. M.; Peverati, R.; Prociuk, A.; Rehn, D. R.; Rosta, E.; Russ, N. J.; Sharada, S. M.; Sharma, S.; Small, D. W.; Sodt, A.; Stein, T.; Stück, D.; Su, Y.-C.; Thom, A. J. W.; Tsuchimochi, T.; Vanovschi, V.; Vogt, L.; Vydrov, O.; Wang, T.; Watson, M. A.; Wenzel, J.; White, A.; Williams, C. F.; Yang, J.; Yeganeh, S.; Yost, S. R.; You, Z.-Q.; Zhang, I. Y.; Zhang, X.; Zhao, Y.; Brooks, B. R.; Chan, G. K. L.; Chipman, D. M.; Cramer, C. J.; Goddard Iii, W. A.; Gordon, M. S.; Hehre, W. J.; Klamt, A.; Schaefer, H. F., III; Schmidt, M. W.; Sherrill, C. D.; Truhlar, D. G.; Warshel, A.; Xu, X.; Aspuru-Guzik, A.; Baer, R.; Bell, A. T.; Besley, N. A.; Chai, J.-D.; Dreuw, A.; Dunietz, B. D.; Furlani, T. R.; Gwaltney, S. R.; Hsu, C.-P.; Jung, Y.; Kong, J.; Lambrecht, D. S.; Liang, W.; Ochsenfeld, C.; Rassolov, V. A.; Slipchenko, L. V.; Subotnik, J. E.; Van Voorhis, T.; Herbert, J. M.;

Krylov, A. I.; Gill, P. M. W.; Head-Gordon, M. Advances in Molecular Quantum Chemistry Contained in the Q-Chem 4 Program Package. *Molecular Physics* **2014**, *113*, 184–215.
8. CYLview, 1.0b; Legault, C. Y., Université de Sherbrooke, 2009 (http://www.cylview.org).

III. Experimental Data

X-ray Data for 27

X-ray Data Collection, Structure Solution and Refinement for ***27*** *cdv88.*

A colorless crystal of approximate dimensions 0.221 x 0.289 x 0.360 mm was mounted in a cryoloop and transferred to a Bruker X8 Prospector diffractometer system. The APEX3[1] program package was used to determine the unit-cell parameters and for data collection (4-20 sec/frame scan time). The raw frame data was processed using SAINT[2] and SADABS[3] to yield the reflection data file. Subsequent calculations were carried out using the SHELXTL[4] program package. There were no systematic absences. The noncentrosymmetric triclinic space group *P*1 was assigned and later determined to be correct.

The structure was solved by direct methods and refined on F^2 by full-matrix least-squares techniques[4]. The analytical scattering factors[5] for neutral atoms were used throughout the analysis. Hydrogen atoms H(1), H(2) and H(3) were located from a difference-Fourier map and refined (x,y,z and U_{iso}). The remaining hydrogen atoms were included using a riding model. There were two molecules of dichloromethane solvent present.

Least-squares analysis yielded wR2 = 0.1491 and Goof = 1.027 for 441 variables refined against 6426 data (0.83 Å), R1 = 0.0551 for those 6381 data with $I > 2.0\sigma(I)$. The absolute structure was assigned by refinement of the Flack parameter[6]

References:

1. APEX3 Version 2018.1-0, Bruker AXS, Inc.; Madison, WI 2018.

2. SAINT Version 8.38a, Bruker AXS, Inc.; Madison, WI 2013.

3. Sheldrick, G. M. SADABS, Version 2014/5, Bruker AXS, Inc.; Madison, WI 2014.

4. Sheldrick, G. M. SHELXTL, Version 2014/7, Bruker AXS, Inc.; Madison, WI 2014.

5. International Tables for Crystallography 1992, Vol. C., Dordrecht: Kluwer Academic Publishers.

6. Parsons, S., Flack, H. D., Wagner, T. Acta Cryst. B69, 249-259, 2013.

Definitions:

$wR2 = [\Sigma[w(F_o^2-F_c^2)^2] / \Sigma[w(F_o^2)^2]]^{1/2}$

$R1 = \Sigma||F_o|-|F_c|| / \Sigma|F_o|$

$Goof = S = [\Sigma[w(F_o^2-F_c^2)^2] / (n-p)]^{1/2}$ where n is the number of reflections and p is the total
number of parameters refined.

The thermal ellipsoid plot is shown at the 50% probability level.

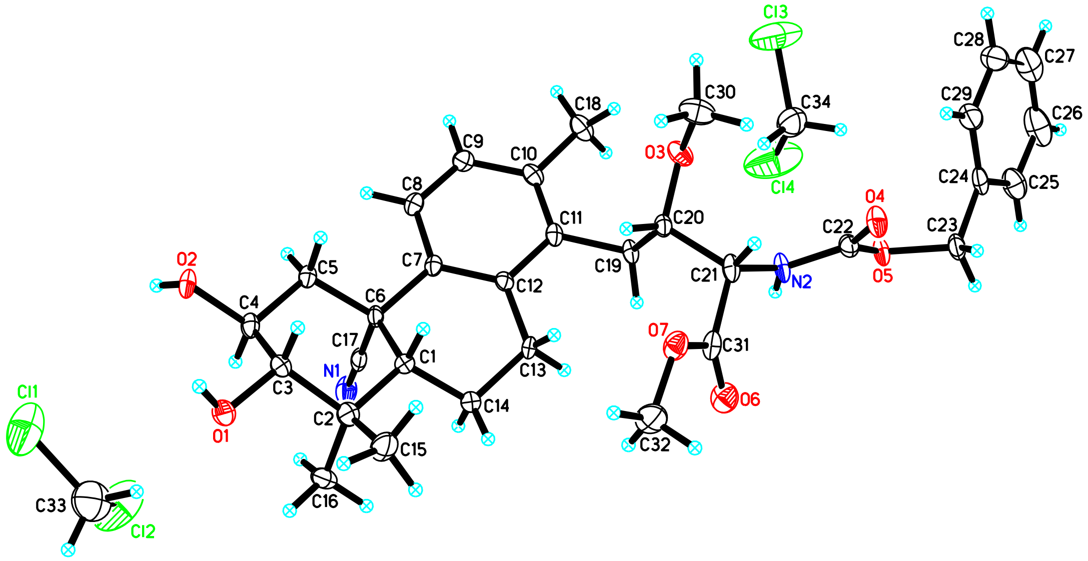
Cl1
Cl2
C33
O1
O2
C4
C3
C5
C6
C17
N1
C2
C1
C16
C15
C14
C13
C12
C7
C8
C9
C10
C11
C18
C19
C20
O3
C30
Cl3
C34
Cl4
C21
N2
C22
O4
O5
C23
C24
C25
C26
C27
C28
C29
O7
C31
O6
C32

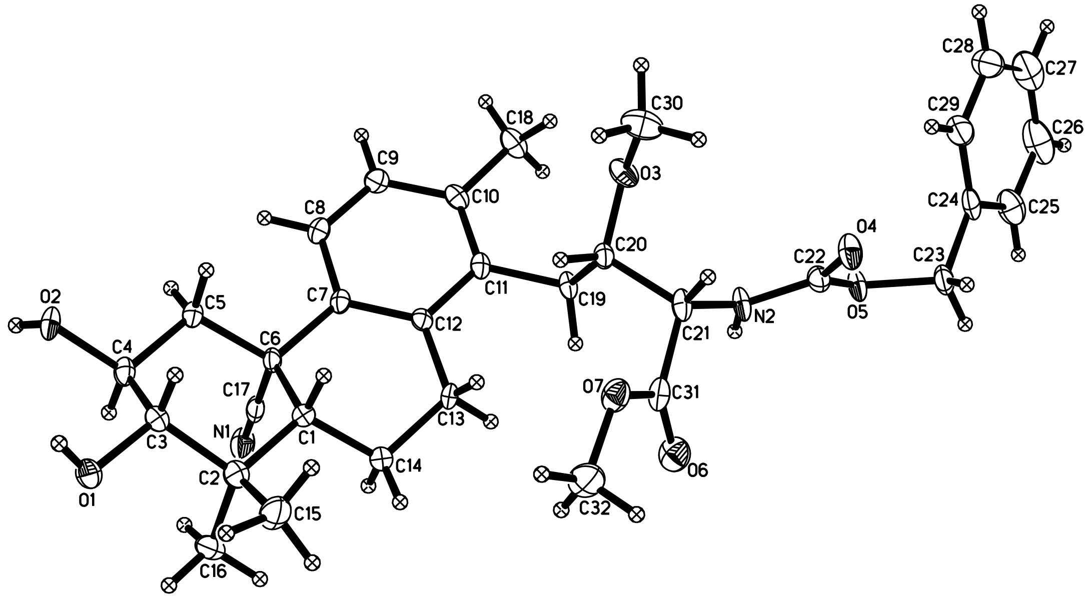
C28
C27
C30
C29
C26
C18
C9
O3
C24
C25
C10
C8
O4
C20
C22
C23
C11
C7
C19
O5
C12
C5
C21
N2
C6
O2
C4
C17
O7
C31
C13
C1
C3
N1
C14
O6
C2
O1
C15
C32
C16

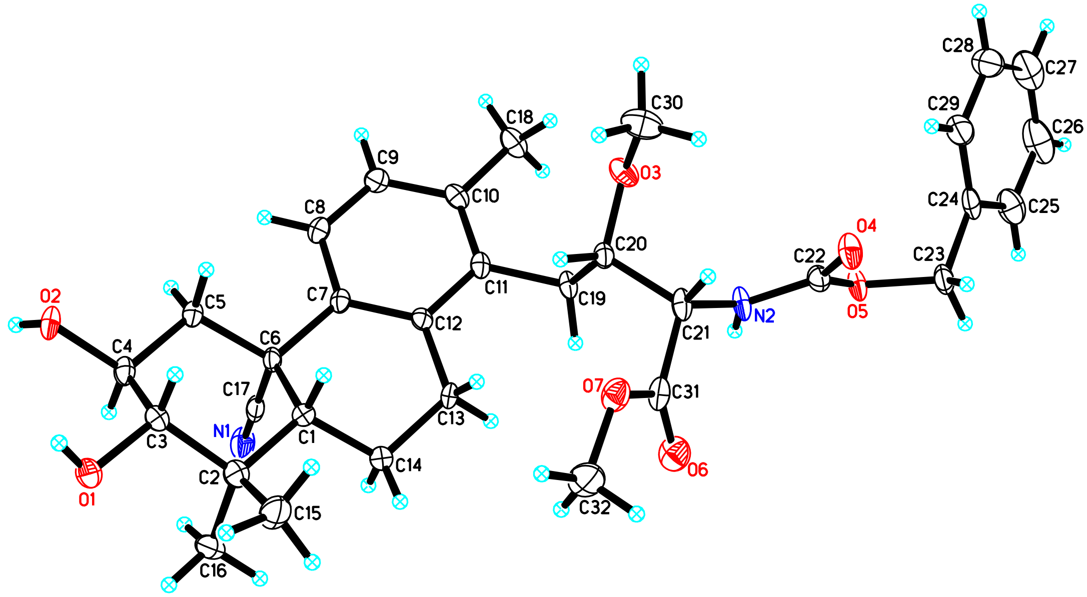
C28
C27
C29
C26
C30
C18
O3
C9
C10
C24
C25
O4
C8
C20
C22
C23
C11
C19
O2
C5
C7
C21
N2
O5
C12
C6
C17
O7
C31
C4
C13
N1
C1
C3
C14
O6
C2
O1
C32
C15
C16

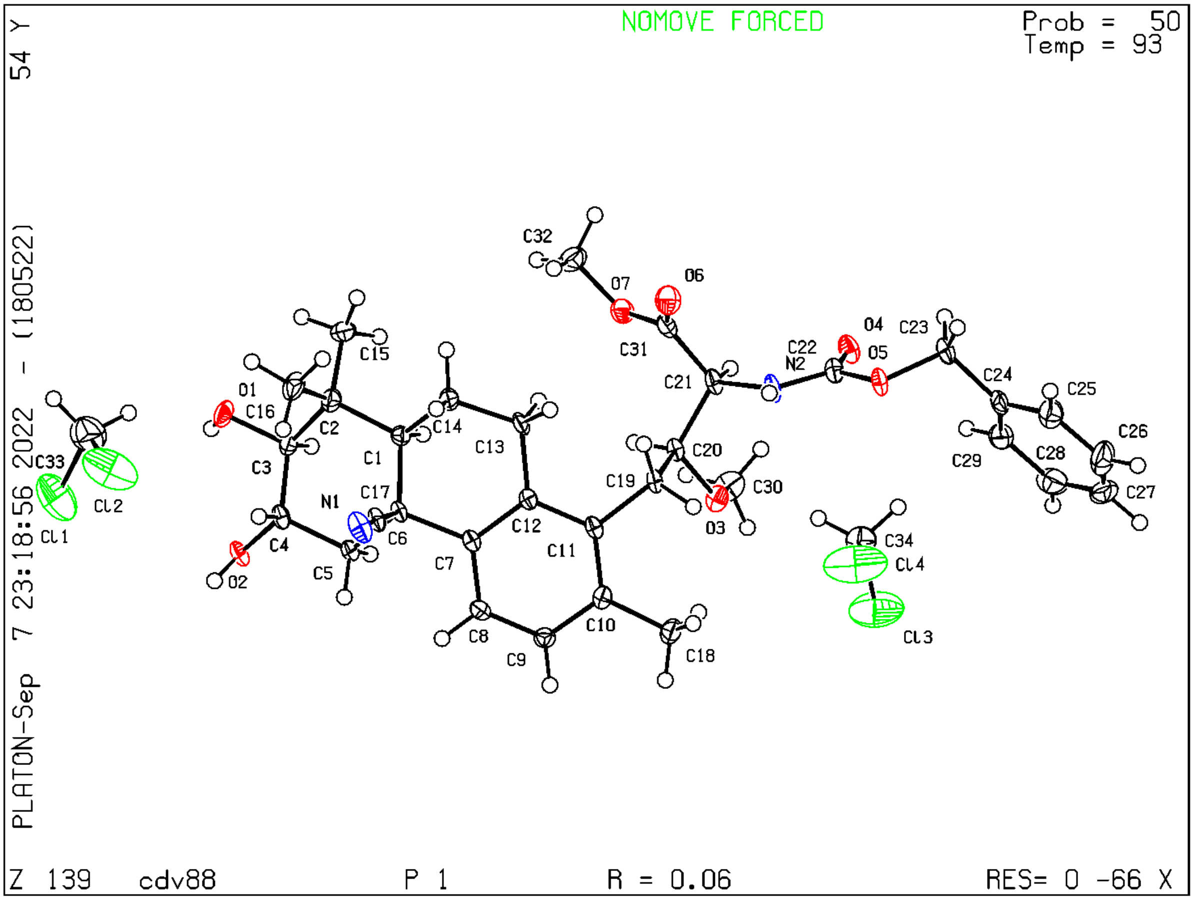
NOMOVE FORCED
Prob = 50
Temp = 93
PLATON-Sep 7 23:18:56 2022 - (180522)
54 Y
Z 139 cdv88
P 1
R = 0.06
RES= 0 -66 X
C32
O7
O6
C31
C21
N2
C22
O4
O5
C23
C24
C25
C26
C27
C28
C29
C20
C30
O3
C19
C34
CL4
CL3
C18
C10
C9
C8
C7
C11
C12
C13
C14
C15
C1
C2
C16
O1
C3
C33
CL2
CL1
N1
C17
C6
C4
C5
O2

Table 1. Crystal data and structure refinement for cdv88.

Identification code	cdv88 (Scott Niman)	
Empirical formula	$C_{32} H_{40} N_2 O_7 \cdot 2(CH_2Cl_2)$	
Formula weight	734.51	
Temperature	93(2) K	
Wavelength	1.54178 Å	
Crystal system	Triclinic	
Space group	*P*1	
Unit cell dimensions	a = 7.5443(3) Å	α = 107.3243(14)°.
	b = 11.5907(5) Å	β = 106.7767(14)°.
	c = 11.7864(5) Å	γ = 99.3389(14)°.
Volume	906.61(7) $Å^3$	
Z	1	
Density (calculated)	1.345 Mg/m^3	
Absorption coefficient	3.366 mm^{-1}	
F(000)	386	
Crystal color	colorless	
Crystal size	0.360 x 0.289 x 0.221 mm^3	
Theta range for data collection	4.151 to 68.935°	
Index ranges	$-9 \leq h \leq 9$, $-14 \leq k \leq 13$, $-14 \leq l \leq 14$	
Reflections collected	24240	
Independent reflections	6426 [R(int) = 0.0390]	
Completeness to theta = 67.679°	99.7 %	
Absorption correction	Semi-empirical from equivalents	
Max. and min. transmission	0.6614 and 0.5667	
Refinement method	Full-matrix least-squares on F^2	
Data / restraints / parameters	6426 / 3 / 441	
Goodness-of-fit on F^2	1.027	
Final R indices [I>2sigma(I) = 6381 data]	R1 = 0.0551, wR2 = 0.1489	
R indices (all data, 0.83 Å)	R1 = 0.0553, wR2 = 0.1491	
Absolute structure parameter	0.001(9)	
Largest diff. peak and hole	1.050 and -0.621 e.$Å^{-3}$	

Table 2. Atomic coordinates (x 10^4) and equivalent isotropic displacement parameters ($Å^2$ x 10^3) for cdv88. U(eq) is defined as one third of the trace of the orthogonalized U^{ij} tensor.

	x	y	z	U(eq)
O(1)	6910(5)	586(3)	8344(3)	25(1)
O(2)	6439(4)	-670(3)	5812(3)	19(1)
O(3)	1026(4)	5542(3)	3523(3)	21(1)
O(4)	-409(4)	8551(3)	5184(3)	24(1)
O(5)	1990(4)	9765(3)	4894(3)	21(1)
O(6)	4645(5)	8122(3)	7754(3)	28(1)
O(7)	2515(5)	6359(3)	7421(3)	24(1)
N(1)	10850(6)	3448(4)	6441(4)	27(1)
N(2)	2483(5)	8124(3)	5382(4)	20(1)
C(1)	6554(6)	3183(4)	7063(4)	17(1)
C(2)	7108(7)	2592(4)	8096(4)	23(1)
C(3)	6381(6)	1151(4)	7418(4)	17(1)
C(4)	7146(6)	666(4)	6360(4)	17(1)
C(5)	6485(6)	1225(4)	5342(4)	15(1)
C(6)	7109(6)	2673(4)	5876(4)	15(1)
C(7)	6182(5)	3217(4)	4873(4)	14(1)
C(8)	5797(6)	2545(4)	3586(4)	16(1)
C(9)	5002(6)	3025(4)	2672(4)	18(1)
C(10)	4547(5)	4176(4)	3000(4)	16(1)
C(11)	4886(5)	4841(4)	4287(4)	14(1)
C(12)	5721(5)	4363(4)	5230(4)	14(1)
C(13)	6037(6)	5088(4)	6600(4)	20(1)
C(14)	7213(7)	4612(4)	7562(4)	21(1)
C(15)	6028(8)	2987(5)	9017(4)	29(1)
C(16)	9265(7)	3007(5)	8883(4)	28(1)
C(17)	9222(6)	3115(4)	6210(4)	18(1)
C(18)	3757(7)	4667(5)	1961(4)	24(1)
C(19)	4345(6)	6067(4)	4697(4)	16(1)
C(20)	2329(6)	5858(4)	4778(4)	16(1)
C(21)	2050(6)	7027(4)	5702(4)	18(1)
C(22)	1211(6)	8786(4)	5168(4)	19(1)

C(23)	1038(6)	10758(4)	4935(4)	22(1)
C(24)	14(6)	10741(4)	3628(4)	22(1)
C(25)	640(7)	11753(5)	3296(5)	30(1)
C(26)	-335(9)	11788(6)	2126(6)	41(1)
C(27)	-1915(9)	10823(7)	1274(5)	42(1)
C(28)	-2543(8)	9799(6)	1593(5)	38(1)
C(29)	-1584(7)	9773(5)	2772(5)	28(1)
C(30)	-931(7)	5027(5)	3336(5)	31(1)
C(31)	3243(6)	7267(4)	7079(4)	20(1)
C(32)	3508(8)	6441(5)	8709(5)	31(1)
C(33)	10773(10)	-242(7)	9314(7)	53(2)
Cl(1)	9737(3)	-1802(2)	8216(2)	67(1)
Cl(2)	12557(3)	544(2)	8923(2)	77(1)
C(34)	1322(9)	7677(6)	2175(5)	38(1)
Cl(3)	-187(3)	6644(2)	668(2)	79(1)
Cl(4)	3595(3)	8314(3)	2188(2)	88(1)

Table 3. Bond lengths [Å] and angles [°] for cdv88.

O(1)-C(3)	1.428(5)
O(2)-C(4)	1.427(5)
O(3)-C(20)	1.417(5)
O(3)-C(30)	1.425(5)
O(4)-C(22)	1.215(6)
O(5)-C(22)	1.359(5)
O(5)-C(23)	1.449(5)
O(6)-C(31)	1.200(6)
O(7)-C(31)	1.329(5)
O(7)-C(32)	1.452(6)
N(1)-C(17)	1.150(6)
N(2)-C(22)	1.337(6)
N(2)-C(21)	1.445(5)
C(1)-C(14)	1.523(6)
C(1)-C(6)	1.552(6)
C(1)-C(2)	1.556(5)
C(2)-C(16)	1.533(7)
C(2)-C(3)	1.544(6)
C(2)-C(15)	1.545(7)
C(3)-C(4)	1.519(6)
C(4)-C(5)	1.527(5)
C(5)-C(6)	1.542(5)
C(6)-C(17)	1.487(6)
C(6)-C(7)	1.546(5)
C(7)-C(8)	1.400(6)
C(7)-C(12)	1.401(6)
C(8)-C(9)	1.388(6)
C(9)-C(10)	1.402(6)
C(10)-C(11)	1.406(6)
C(10)-C(18)	1.511(5)
C(11)-C(12)	1.420(5)
C(11)-C(19)	1.520(6)
C(12)-C(13)	1.508(6)
C(13)-C(14)	1.526(5)

C(19)-C(20)	1.536(5)
C(20)-C(21)	1.554(6)
C(21)-C(31)	1.526(6)
C(23)-C(24)	1.503(6)
C(24)-C(29)	1.384(7)
C(24)-C(25)	1.398(7)
C(25)-C(26)	1.379(8)
C(26)-C(27)	1.374(10)
C(27)-C(28)	1.400(10)
C(28)-C(29)	1.380(7)
C(33)-Cl(2)	1.751(8)
C(33)-Cl(1)	1.766(8)
C(34)-Cl(3)	1.735(6)
C(34)-Cl(4)	1.748(6)

C(20)-O(3)-C(30)	114.4(3)
C(22)-O(5)-C(23)	117.5(3)
C(31)-O(7)-C(32)	116.3(4)
C(22)-N(2)-C(21)	121.7(4)
C(14)-C(1)-C(6)	108.1(3)
C(14)-C(1)-C(2)	114.8(3)
C(6)-C(1)-C(2)	116.7(3)
C(16)-C(2)-C(3)	111.2(4)
C(16)-C(2)-C(15)	108.0(4)
C(3)-C(2)-C(15)	107.9(4)
C(16)-C(2)-C(1)	113.7(4)
C(3)-C(2)-C(1)	107.6(3)
C(15)-C(2)-C(1)	108.3(4)
O(1)-C(3)-C(4)	110.6(3)
O(1)-C(3)-C(2)	108.6(3)
C(4)-C(3)-C(2)	112.8(3)
O(2)-C(4)-C(3)	108.7(3)
O(2)-C(4)-C(5)	109.8(3)
C(3)-C(4)-C(5)	110.1(3)
C(4)-C(5)-C(6)	112.9(3)
C(17)-C(6)-C(5)	109.1(3)

C(17)-C(6)-C(7)	105.9(3)
C(5)-C(6)-C(7)	111.8(3)
C(17)-C(6)-C(1)	110.6(3)
C(5)-C(6)-C(1)	110.0(3)
C(7)-C(6)-C(1)	109.3(3)
C(8)-C(7)-C(12)	119.7(4)
C(8)-C(7)-C(6)	119.1(3)
C(12)-C(7)-C(6)	121.3(3)
C(9)-C(8)-C(7)	120.1(4)
C(8)-C(9)-C(10)	121.5(4)
C(9)-C(10)-C(11)	118.7(4)
C(9)-C(10)-C(18)	118.7(4)
C(11)-C(10)-C(18)	122.6(4)
C(10)-C(11)-C(12)	120.1(4)
C(10)-C(11)-C(19)	121.2(3)
C(12)-C(11)-C(19)	118.7(4)
C(7)-C(12)-C(11)	119.9(4)
C(7)-C(12)-C(13)	121.3(3)
C(11)-C(12)-C(13)	118.8(4)
C(12)-C(13)-C(14)	114.9(3)
C(1)-C(14)-C(13)	109.5(3)
N(1)-C(17)-C(6)	178.6(5)
C(11)-C(19)-C(20)	112.0(3)
O(3)-C(20)-C(19)	105.9(3)
O(3)-C(20)-C(21)	110.2(3)
C(19)-C(20)-C(21)	113.4(3)
N(2)-C(21)-C(31)	111.4(4)
N(2)-C(21)-C(20)	111.7(3)
C(31)-C(21)-C(20)	110.7(3)
O(4)-C(22)-N(2)	126.2(4)
O(4)-C(22)-O(5)	124.4(4)
N(2)-C(22)-O(5)	109.4(4)
O(5)-C(23)-C(24)	112.2(4)
C(29)-C(24)-C(25)	119.5(4)
C(29)-C(24)-C(23)	121.4(4)
C(25)-C(24)-C(23)	119.1(4)

C(26)-C(25)-C(24)	120.1(5)
C(27)-C(26)-C(25)	120.3(5)
C(26)-C(27)-C(28)	120.0(5)
C(29)-C(28)-C(27)	119.7(5)
C(28)-C(29)-C(24)	120.4(5)
O(6)-C(31)-O(7)	124.4(4)
O(6)-C(31)-C(21)	126.1(4)
O(7)-C(31)-C(21)	109.4(4)
Cl(2)-C(33)-Cl(1)	110.6(4)
Cl(3)-C(34)-Cl(4)	111.5(3)

Table 4. Anisotropic displacement parameters ($Å^2 \times 10^3$) for cdv88. The anisotropic displacement factor exponent takes the form: $-2\pi^2[h^2 a^{*2}U^{11} + ... + 2 h k a^* b^* U^{12}]$

	U^{11}	U^{22}	U^{33}	U^{23}	U^{13}	U^{12}
O(1)	36(2)	17(2)	21(2)	12(1)	4(1)	5(2)
O(2)	19(2)	11(1)	30(2)	9(1)	13(1)	7(1)
O(3)	12(1)	28(2)	25(2)	14(1)	7(1)	6(1)
O(4)	20(2)	23(2)	36(2)	14(1)	14(1)	12(1)
O(5)	19(2)	18(1)	32(2)	15(1)	11(1)	11(1)
O(6)	31(2)	22(2)	33(2)	13(1)	12(1)	4(1)
O(7)	32(2)	17(2)	26(2)	9(1)	13(1)	6(1)
N(1)	19(2)	18(2)	41(2)	9(2)	8(2)	6(2)
N(2)	16(2)	17(2)	35(2)	16(2)	12(2)	10(2)
C(1)	18(2)	14(2)	14(2)	4(2)	3(2)	5(2)
C(2)	31(2)	18(2)	19(2)	8(2)	6(2)	8(2)
C(3)	20(2)	16(2)	16(2)	8(2)	5(2)	6(2)
C(4)	15(2)	13(2)	25(2)	9(2)	8(2)	4(2)
C(5)	15(2)	12(2)	20(2)	6(2)	7(2)	4(2)
C(6)	14(2)	12(2)	19(2)	6(2)	6(2)	7(2)
C(7)	11(2)	10(2)	19(2)	6(2)	6(1)	2(2)
C(8)	18(2)	14(2)	19(2)	5(2)	10(2)	5(2)
C(9)	18(2)	19(2)	17(2)	6(2)	8(2)	6(2)
C(10)	11(2)	21(2)	20(2)	12(2)	7(2)	5(2)
C(11)	9(2)	13(2)	23(2)	8(2)	8(2)	4(1)
C(12)	8(2)	14(2)	18(2)	5(2)	3(1)	3(1)
C(13)	24(2)	12(2)	20(2)	3(2)	4(2)	10(2)
C(14)	25(2)	16(2)	17(2)	4(2)	2(2)	8(2)
C(15)	49(3)	24(2)	19(2)	8(2)	16(2)	13(2)
C(16)	31(2)	23(2)	20(2)	8(2)	-5(2)	4(2)
C(17)	20(2)	11(2)	24(2)	6(2)	6(2)	6(2)
C(18)	24(2)	33(2)	24(2)	17(2)	10(2)	14(2)
C(19)	13(2)	13(2)	25(2)	11(2)	8(2)	6(2)
C(20)	14(2)	12(2)	21(2)	7(2)	6(2)	5(2)
C(21)	17(2)	15(2)	30(2)	13(2)	12(2)	7(2)
C(22)	18(2)	15(2)	23(2)	8(2)	4(2)	5(2)

C(23)	19(2)	20(2)	29(2)	10(2)	8(2)	12(2)
C(24)	23(2)	25(2)	28(2)	14(2)	14(2)	18(2)
C(25)	27(2)	38(3)	37(3)	22(2)	18(2)	16(2)
C(26)	46(3)	59(4)	52(3)	43(3)	32(3)	32(3)
C(27)	54(4)	68(4)	26(2)	25(3)	25(3)	41(3)
C(28)	30(3)	45(3)	27(2)	2(2)	4(2)	17(2)
C(29)	27(2)	33(3)	26(2)	10(2)	8(2)	15(2)
C(30)	14(2)	43(3)	31(2)	14(2)	5(2)	-1(2)
C(31)	24(2)	16(2)	27(2)	10(2)	15(2)	10(2)
C(32)	45(3)	29(2)	23(2)	12(2)	14(2)	9(2)
C(33)	47(4)	63(4)	55(4)	19(3)	21(3)	25(3)
Cl(1)	54(1)	40(1)	105(2)	20(1)	34(1)	13(1)
Cl(2)	56(1)	59(1)	100(2)	1(1)	47(1)	-3(1)
C(34)	48(3)	35(3)	31(3)	10(2)	13(2)	13(2)
Cl(3)	64(1)	82(1)	43(1)	-11(1)	2(1)	-8(1)
Cl(4)	61(1)	104(2)	63(1)	-3(1)	30(1)	-21(1)

Table 5. Hydrogen coordinates (x 10^4) and isotropic displacement parameters (Å^2 x 10^3) for cdv88.

	x	y	z	U(eq)
H(1)	6250(90)	-40(70)	8040(60)	24(16)
H(2)	7300(130)	-980(80)	5620(80)	60(20)
H(3)	3660(100)	8520(60)	5520(60)	31(15)
H(1A)	5116	2962	6734	20
H(3A)	4945	914	7036	21
H(4A)	8584	908	6722	20
H(5A)	5065	931	4944	18
H(5B)	7021	915	4671	18
H(8A)	6081	1759	3339	20
H(9A)	4761	2564	1803	21
H(13A)	4767	5061	6689	24
H(13B)	6696	5977	6813	24
H(14A)	8596	4893	7701	25
H(14B)	7041	4960	8386	25
H(15A)	5933	2389	9449	44
H(15B)	4734	2994	8536	44
H(15C)	6733	3829	9650	44
H(16A)	9504	2638	9542	42
H(16B)	9695	3924	9284	42
H(16C)	9976	2722	8325	42
H(18A)	3646	4067	1140	36
H(18B)	4626	5477	2138	36
H(18C)	2485	4776	1932	36
H(19A)	4386	6484	4080	19
H(19B)	5299	6632	5539	19
H(20A)	2082	5134	5059	19
H(21A)	664	6841	5613	22
H(23A)	2004	11579	5445	26
H(23B)	98	10664	5361	26
H(25A)	1741	12418	3877	36

H(26A)	88	12482	1908	49
H(27A)	-2582	10851	469	50
H(28A)	-3624	9125	1001	45
H(29A)	-2024	9087	2996	34
H(30A)	-1682	4636	2422	47
H(30B)	-1449	5698	3729	47
H(30C)	-1006	4393	3728	47
H(32A)	3055	5642	8797	47
H(32B)	3244	7116	9314	47
H(32C)	4896	6620	8885	47
H(33A)	9762	207	9311	64
H(33B)	11342	-247	10181	64
H(34A)	1456	7229	2778	46
H(34B)	741	8364	2464	46

Table 6. Torsion angles [°] for cdv88.

C(14)-C(1)-C(2)-C(16)	-54.8(5)
C(6)-C(1)-C(2)-C(16)	73.2(5)
C(14)-C(1)-C(2)-C(3)	-178.4(4)
C(6)-C(1)-C(2)-C(3)	-50.4(5)
C(14)-C(1)-C(2)-C(15)	65.2(5)
C(6)-C(1)-C(2)-C(15)	-166.8(4)
C(16)-C(2)-C(3)-O(1)	53.7(5)
C(15)-C(2)-C(3)-O(1)	-64.5(4)
C(1)-C(2)-C(3)-O(1)	178.8(3)
C(16)-C(2)-C(3)-C(4)	-69.3(4)
C(15)-C(2)-C(3)-C(4)	172.5(3)
C(1)-C(2)-C(3)-C(4)	55.8(5)
O(1)-C(3)-C(4)-O(2)	56.7(4)
C(2)-C(3)-C(4)-O(2)	178.6(3)
O(1)-C(3)-C(4)-C(5)	177.1(3)
C(2)-C(3)-C(4)-C(5)	-61.0(4)
O(2)-C(4)-C(5)-C(6)	177.1(3)
C(3)-C(4)-C(5)-C(6)	57.4(4)
C(4)-C(5)-C(6)-C(17)	71.3(4)
C(4)-C(5)-C(6)-C(7)	-171.9(3)
C(4)-C(5)-C(6)-C(1)	-50.2(4)
C(14)-C(1)-C(6)-C(17)	58.9(4)
C(2)-C(1)-C(6)-C(17)	-72.3(4)
C(14)-C(1)-C(6)-C(5)	179.5(3)
C(2)-C(1)-C(6)-C(5)	48.3(5)
C(14)-C(1)-C(6)-C(7)	-57.3(4)
C(2)-C(1)-C(6)-C(7)	171.5(3)
C(17)-C(6)-C(7)-C(8)	86.8(4)
C(5)-C(6)-C(7)-C(8)	-31.9(5)
C(1)-C(6)-C(7)-C(8)	-154.1(3)
C(17)-C(6)-C(7)-C(12)	-93.3(4)
C(5)-C(6)-C(7)-C(12)	148.0(3)
C(1)-C(6)-C(7)-C(12)	25.8(5)
C(12)-C(7)-C(8)-C(9)	1.4(6)

C(6)-C(7)-C(8)-C(9)	-178.7(3)
C(7)-C(8)-C(9)-C(10)	-0.6(6)
C(8)-C(9)-C(10)-C(11)	-0.9(6)
C(8)-C(9)-C(10)-C(18)	177.7(4)
C(9)-C(10)-C(11)-C(12)	1.7(6)
C(18)-C(10)-C(11)-C(12)	-176.8(4)
C(9)-C(10)-C(11)-C(19)	-177.4(3)
C(18)-C(10)-C(11)-C(19)	4.1(6)
C(8)-C(7)-C(12)-C(11)	-0.6(5)
C(6)-C(7)-C(12)-C(11)	179.5(3)
C(8)-C(7)-C(12)-C(13)	177.4(4)
C(6)-C(7)-C(12)-C(13)	-2.5(5)
C(10)-C(11)-C(12)-C(7)	-0.9(5)
C(19)-C(11)-C(12)-C(7)	178.2(3)
C(10)-C(11)-C(12)-C(13)	-179.0(4)
C(19)-C(11)-C(12)-C(13)	0.1(5)
C(7)-C(12)-C(13)-C(14)	10.8(6)
C(11)-C(12)-C(13)-C(14)	-171.1(4)
C(6)-C(1)-C(14)-C(13)	67.2(4)
C(2)-C(1)-C(14)-C(13)	-160.5(4)
C(12)-C(13)-C(14)-C(1)	-43.1(5)
C(10)-C(11)-C(19)-C(20)	93.3(4)
C(12)-C(11)-C(19)-C(20)	-85.8(4)
C(30)-O(3)-C(20)-C(19)	166.0(4)
C(30)-O(3)-C(20)-C(21)	-71.0(4)
C(11)-C(19)-C(20)-O(3)	-83.7(4)
C(11)-C(19)-C(20)-C(21)	155.3(3)
C(22)-N(2)-C(21)-C(31)	-110.7(4)
C(22)-N(2)-C(21)-C(20)	125.0(4)
O(3)-C(20)-C(21)-N(2)	-63.2(4)
C(19)-C(20)-C(21)-N(2)	55.3(4)
O(3)-C(20)-C(21)-C(31)	172.1(3)
C(19)-C(20)-C(21)-C(31)	-69.4(4)
C(21)-N(2)-C(22)-O(4)	-1.8(7)
C(21)-N(2)-C(22)-O(5)	179.7(4)
C(23)-O(5)-C(22)-O(4)	16.0(6)

C(23)-O(5)-C(22)-N(2)	-165.5(4)
C(22)-O(5)-C(23)-C(24)	-107.9(4)
O(5)-C(23)-C(24)-C(29)	67.6(5)
O(5)-C(23)-C(24)-C(25)	-115.3(5)
C(29)-C(24)-C(25)-C(26)	0.4(7)
C(23)-C(24)-C(25)-C(26)	-176.8(5)
C(24)-C(25)-C(26)-C(27)	-0.7(8)
C(25)-C(26)-C(27)-C(28)	0.1(8)
C(26)-C(27)-C(28)-C(29)	0.9(8)
C(27)-C(28)-C(29)-C(24)	-1.3(8)
C(25)-C(24)-C(29)-C(28)	0.6(7)
C(23)-C(24)-C(29)-C(28)	177.7(4)
C(32)-O(7)-C(31)-O(6)	0.6(6)
C(32)-O(7)-C(31)-C(21)	179.2(4)
N(2)-C(21)-C(31)-O(6)	-15.2(6)
C(20)-C(21)-C(31)-O(6)	109.7(5)
N(2)-C(21)-C(31)-O(7)	166.2(3)
C(20)-C(21)-C(31)-O(7)	-69.0(4)

Table 7. Hydrogen bonds for cdv88 [Å and °].

D-H...A	d(D-H)	d(H...A)	d(D...A)	<(DHA)
O(1)-H(1)...O(6)#1	0.73(7)	2.14(7)	2.847(5)	162(6)
O(2)-H(2)...O(4)#2	0.84(9)	2.05(9)	2.887(4)	171(8)
N(2)-H(3)...O(2)#3	0.87(7)	2.04(7)	2.910(5)	176(6)

Symmetry transformations used to generate equivalent atoms:

#1 x,y-1,z #2 x+1,y-1,z #3 x,y+1,z

1D and 2D NMR Spectra

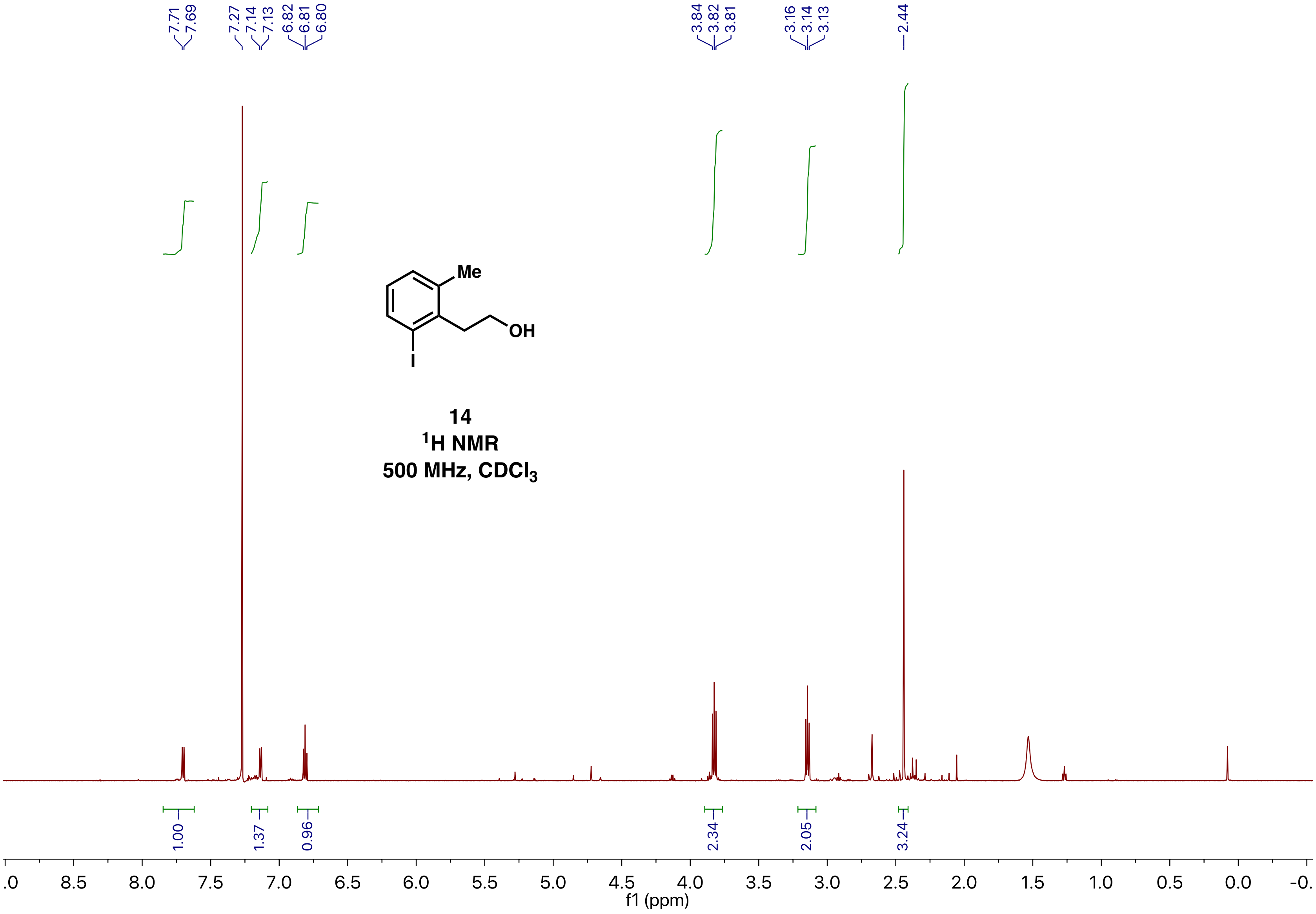

14
^{1}H NMR
500 MHz, $CDCl_3$

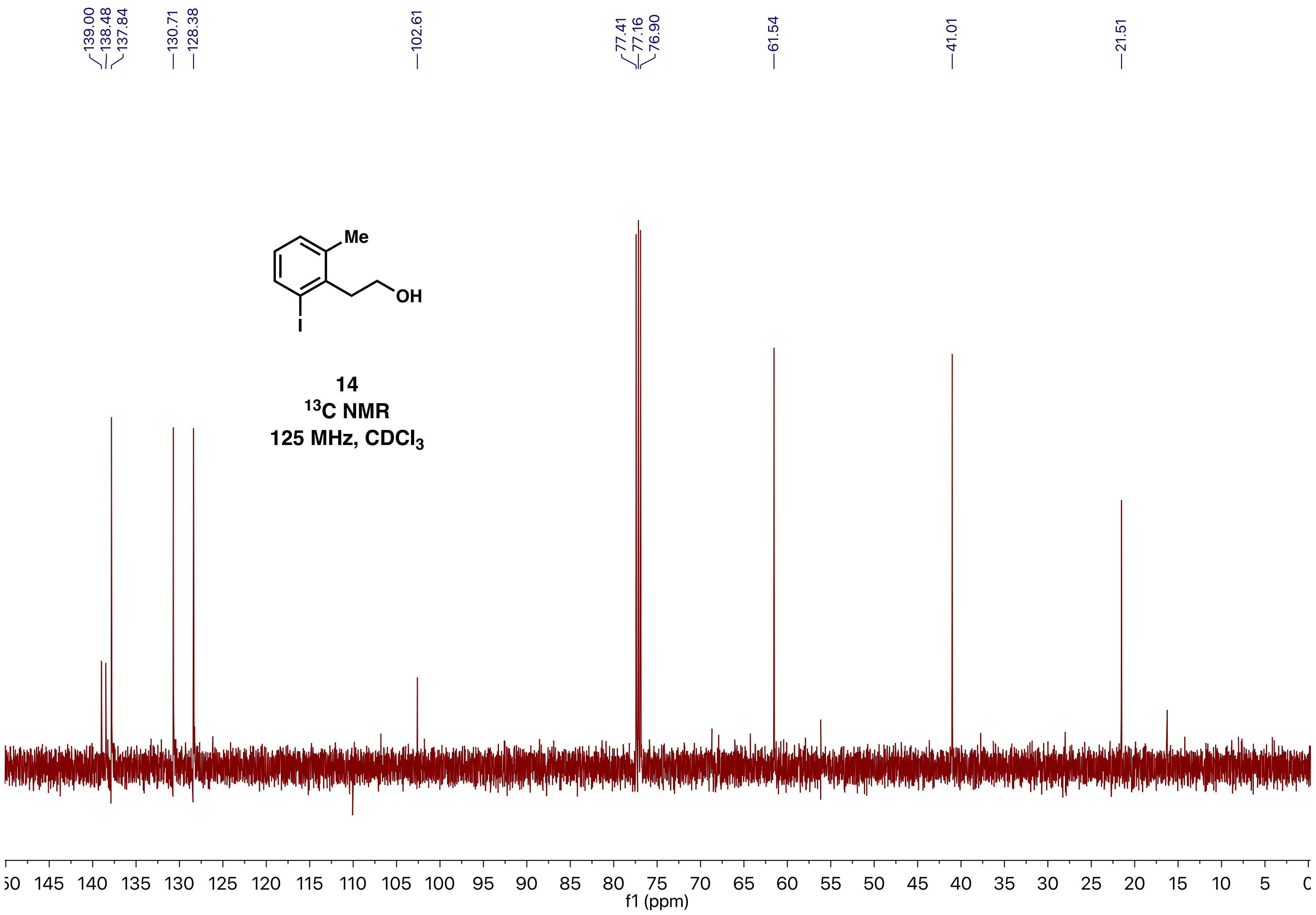
139.00
138.48
137.84
130.71
128.38
102.61
77.41
77.16
76.90
61.54
41.01
21.51
Me
OH
I
14
^{13}C NMR
125 MHz, $CDCl_3$
f1 (ppm)

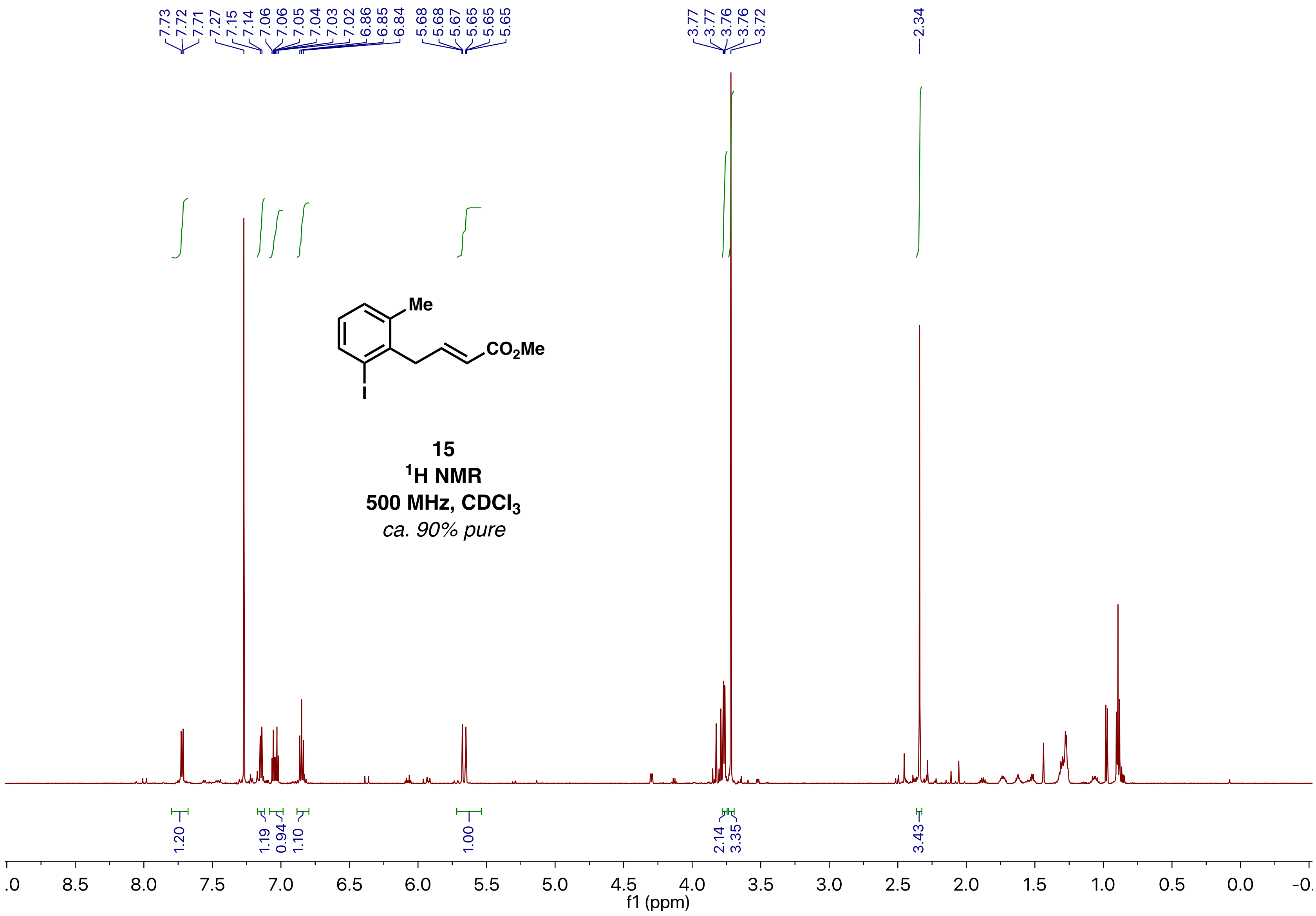

7.73
7.72
7.71
7.27
7.15
7.14
7.06
7.06
7.05
7.04
7.03
7.02
6.86
6.85
6.84
5.68
5.68
5.67
5.65
5.65
5.65
3.77
3.77
3.76
3.76
3.72
2.34
Me
CO_2Me
I
15
^1H NMR
500 MHz, $CDCl_3$
ca. 90% pure
1.20
1.19
0.94
1.10
1.00
2.14
3.35
3.43
f1 (ppm)

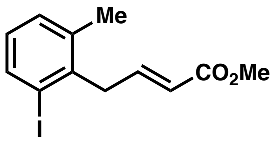

15

^{13}C NMR

125 MHz, $CDCl_3$

ca. 90% pure

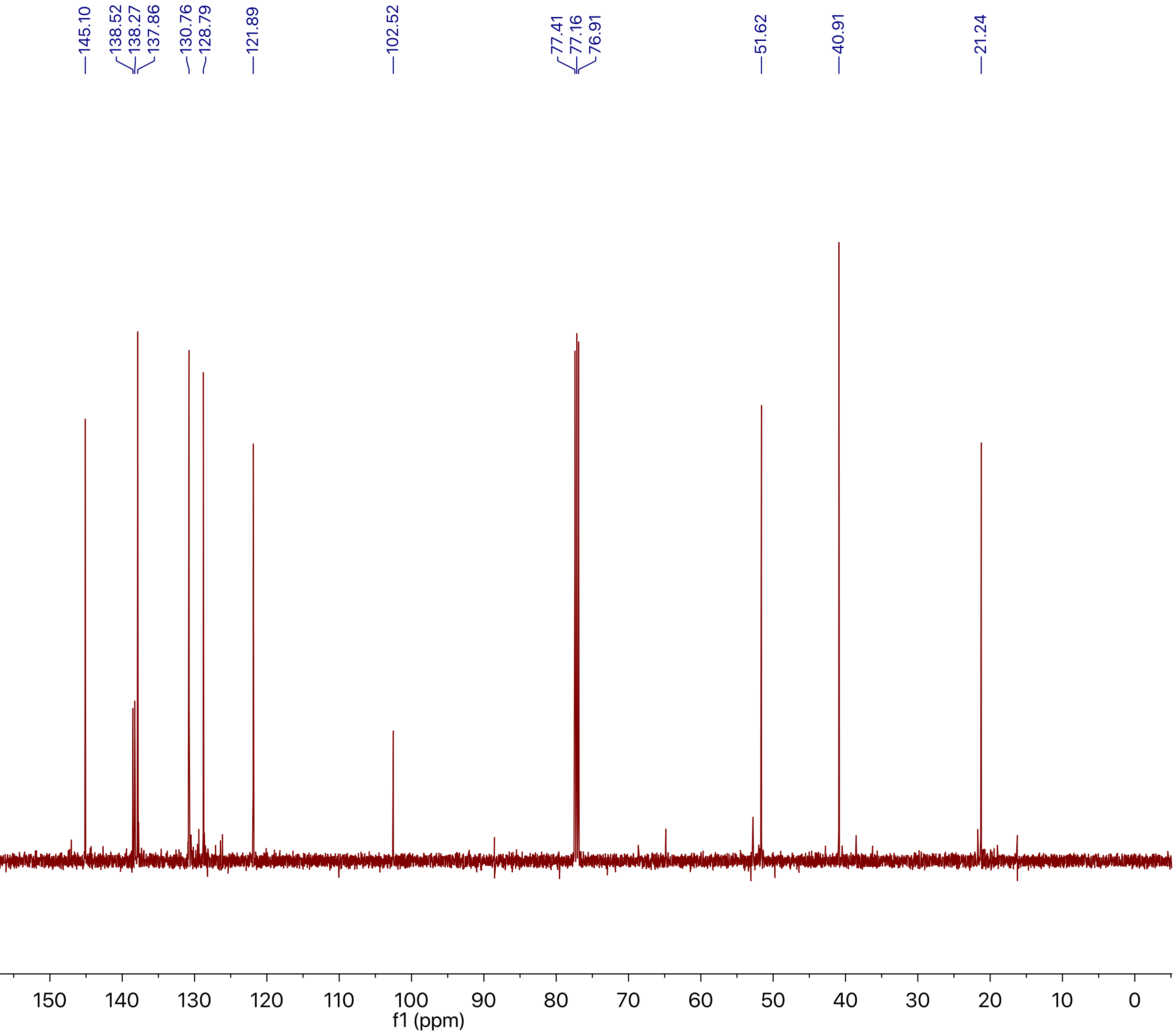

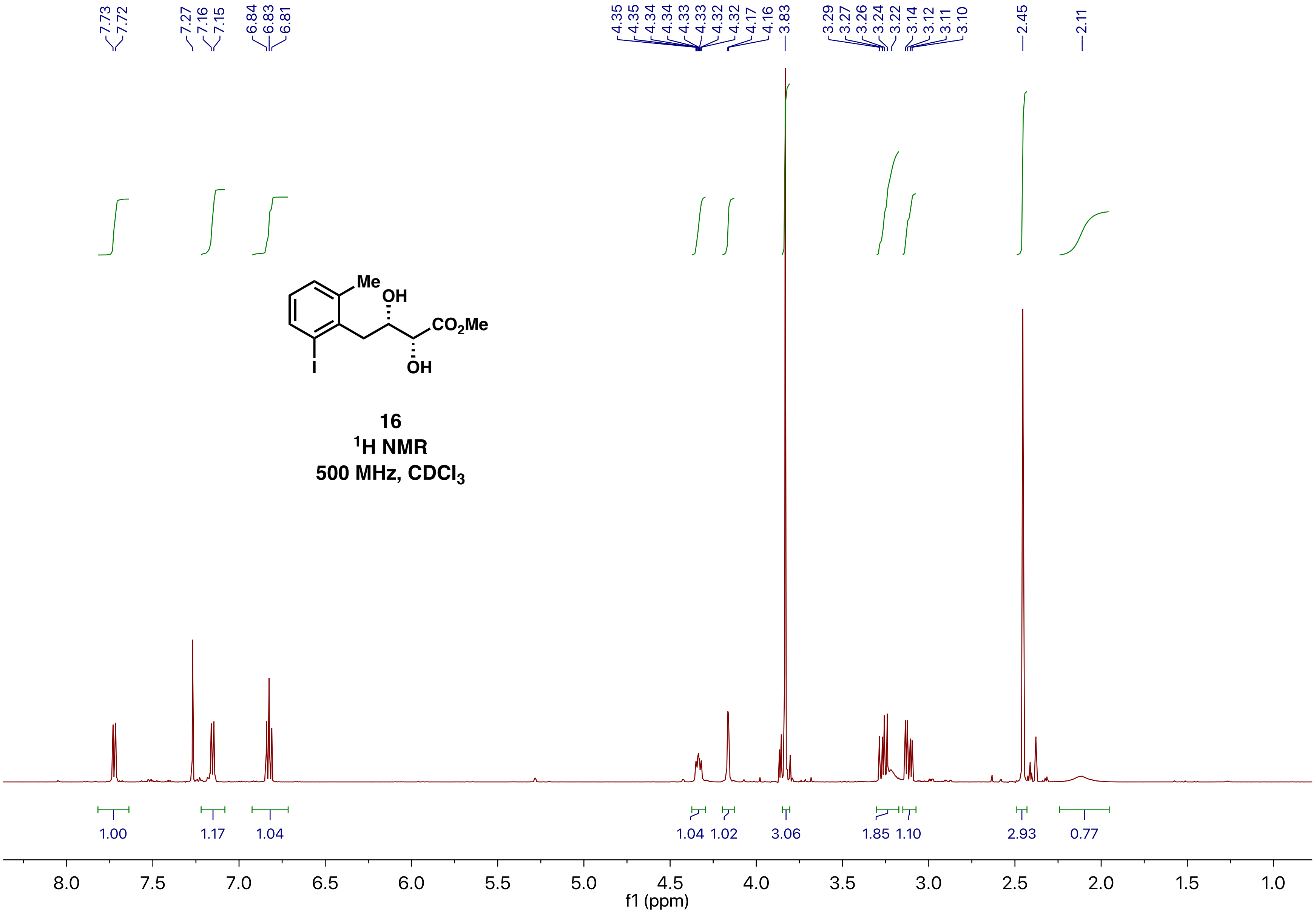
Me
OH
CO2Me
OH
I
16
1H NMR
500 MHz, CDCl3
7.73
7.72
7.27
7.16
7.15
6.84
6.83
6.81
4.35
4.35
4.34
4.34
4.33
4.33
4.32
4.32
4.17
4.16
3.83
3.29
3.27
3.26
3.24
3.22
3.14
3.12
3.11
3.10
2.45
2.11
1.00
1.17
1.04
1.04
1.02
3.06
1.85
1.10
2.93
0.77
8.0
7.5
7.0
6.5
6.0
5.5
5.0
4.5
4.0
3.5
3.0
2.5
2.0
1.5
1.0
f1 (ppm)

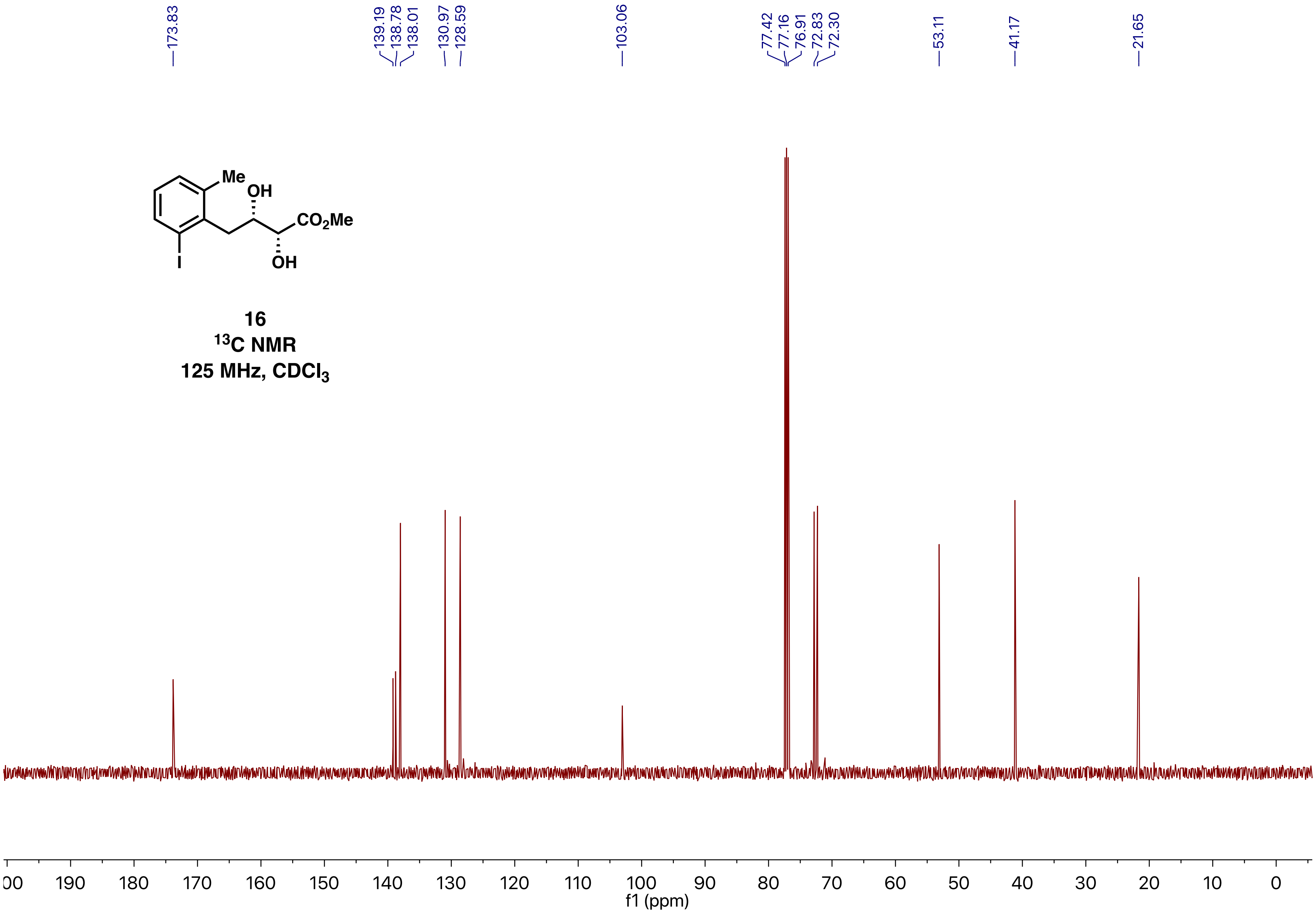

16
^{13}C NMR
125 MHz, $CDCl_3$

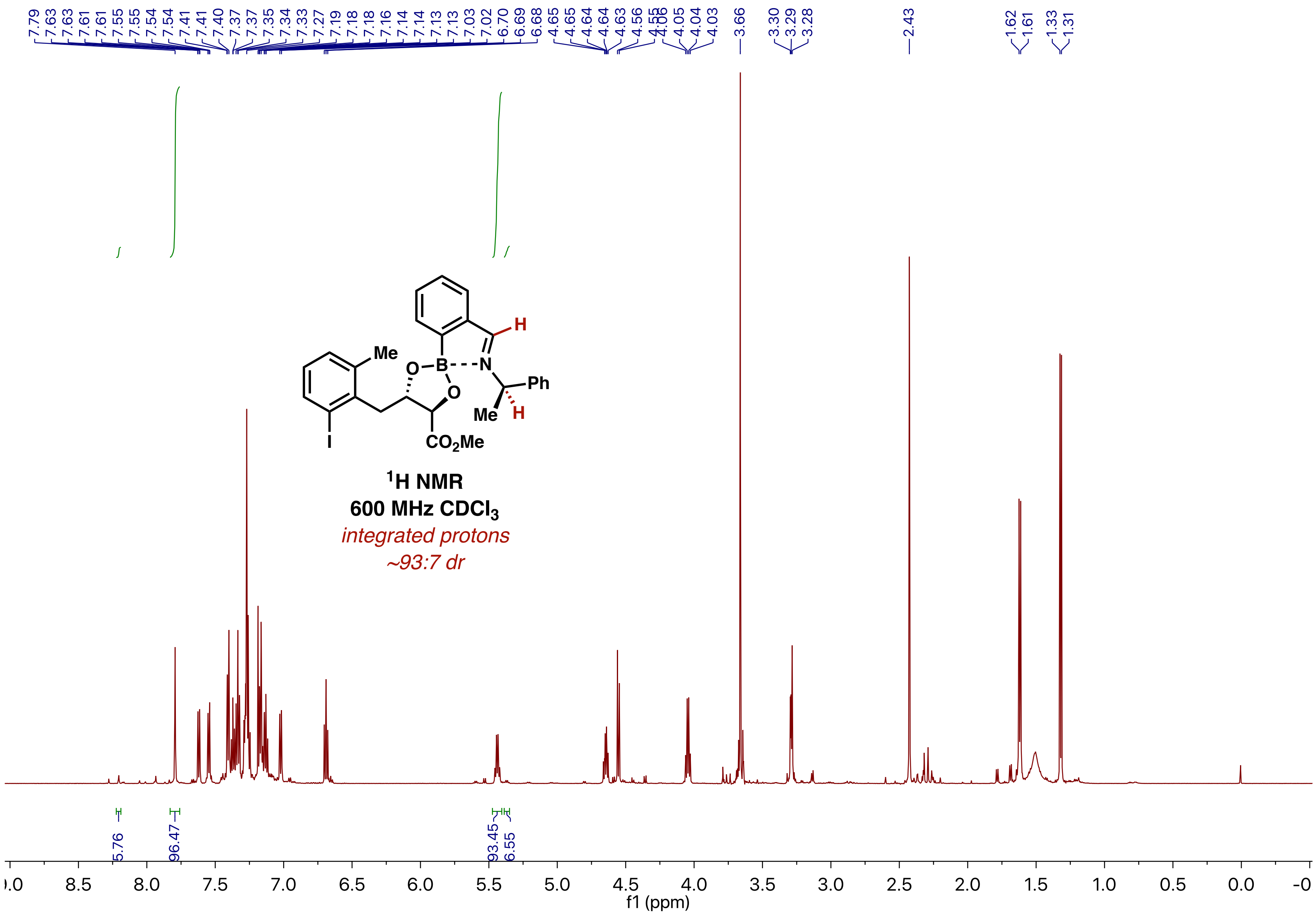

Me
O
B
N
H
O
Ph
Me
H
I
CO2Me
1H NMR
600 MHz CDCl3
integrated protons
~93:7 dr
f1 (ppm)

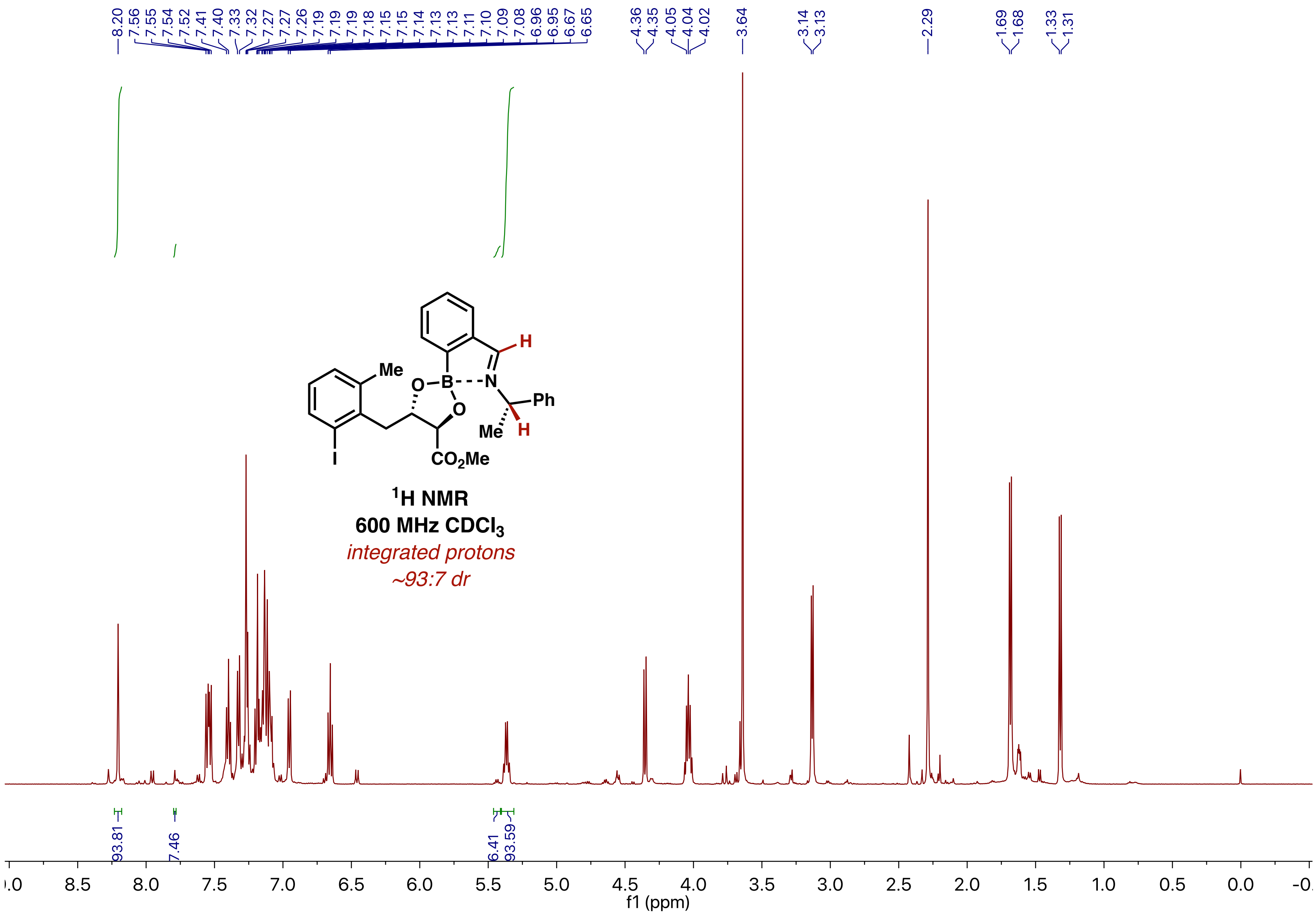

Me
O
B
N
O
H
Ph
Me
H
I
CO2Me
1H NMR
600 MHz CDCl3
integrated protons
~93:7 dr
93.81
7.46
6.41
93.59
f1 (ppm)

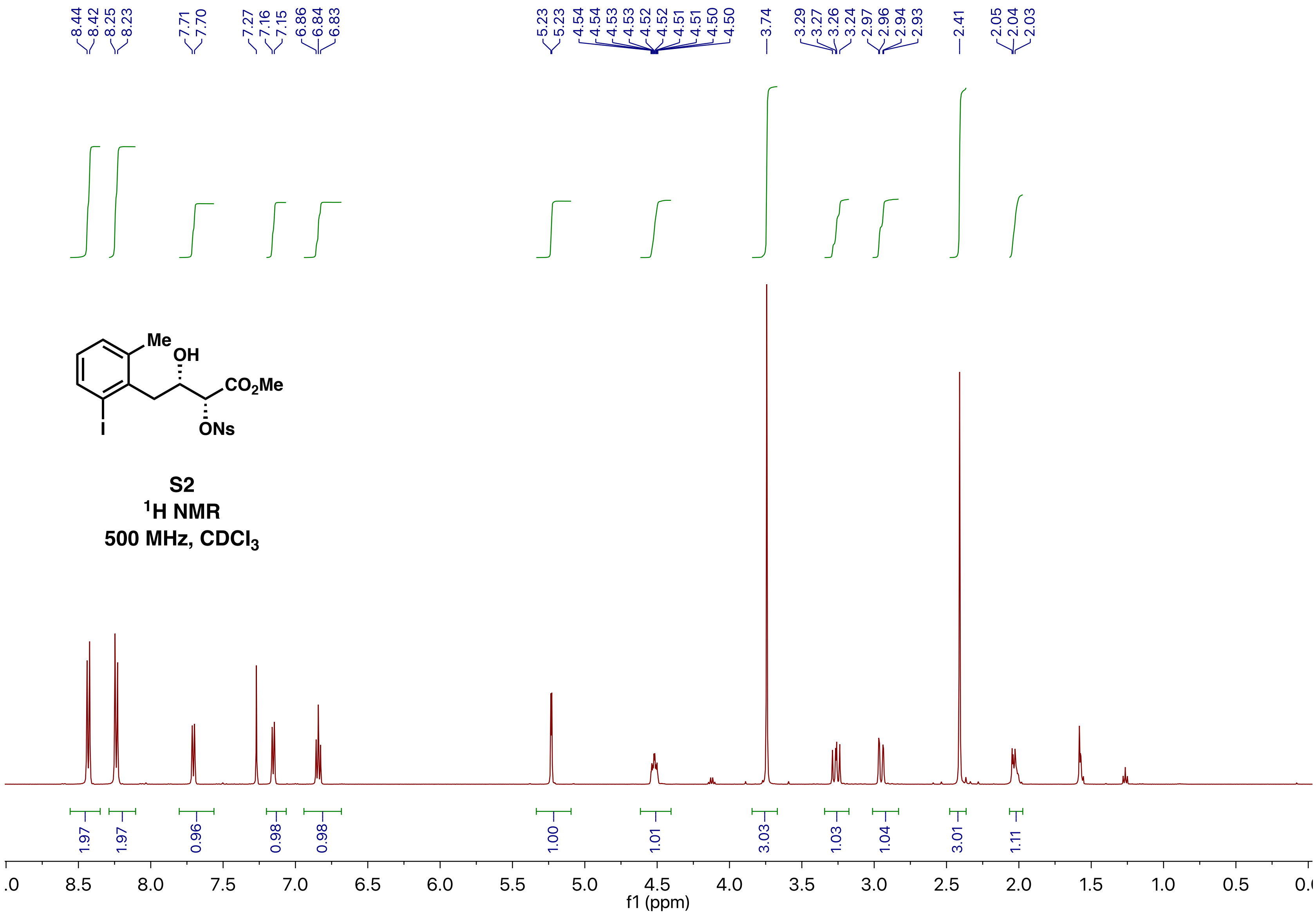

8.44 8.42 8.25 8.23 7.71 7.70 7.27 7.16 7.15 6.86 6.84 6.83 5.23 5.23 4.54 4.54 4.53 4.53 4.52 4.52 4.51 4.51 4.50 4.50 3.74 3.29 3.27 3.26 3.24 2.97 2.96 2.94 2.93 2.41 2.05 2.04 2.03
Me
OH
CO2Me
I
ONs
S2
1H NMR
500 MHz, CDCl3
1.97 1.97 0.96 0.98 0.98 1.00 1.01 3.03 1.03 1.04 3.01 1.11
f1 (ppm)

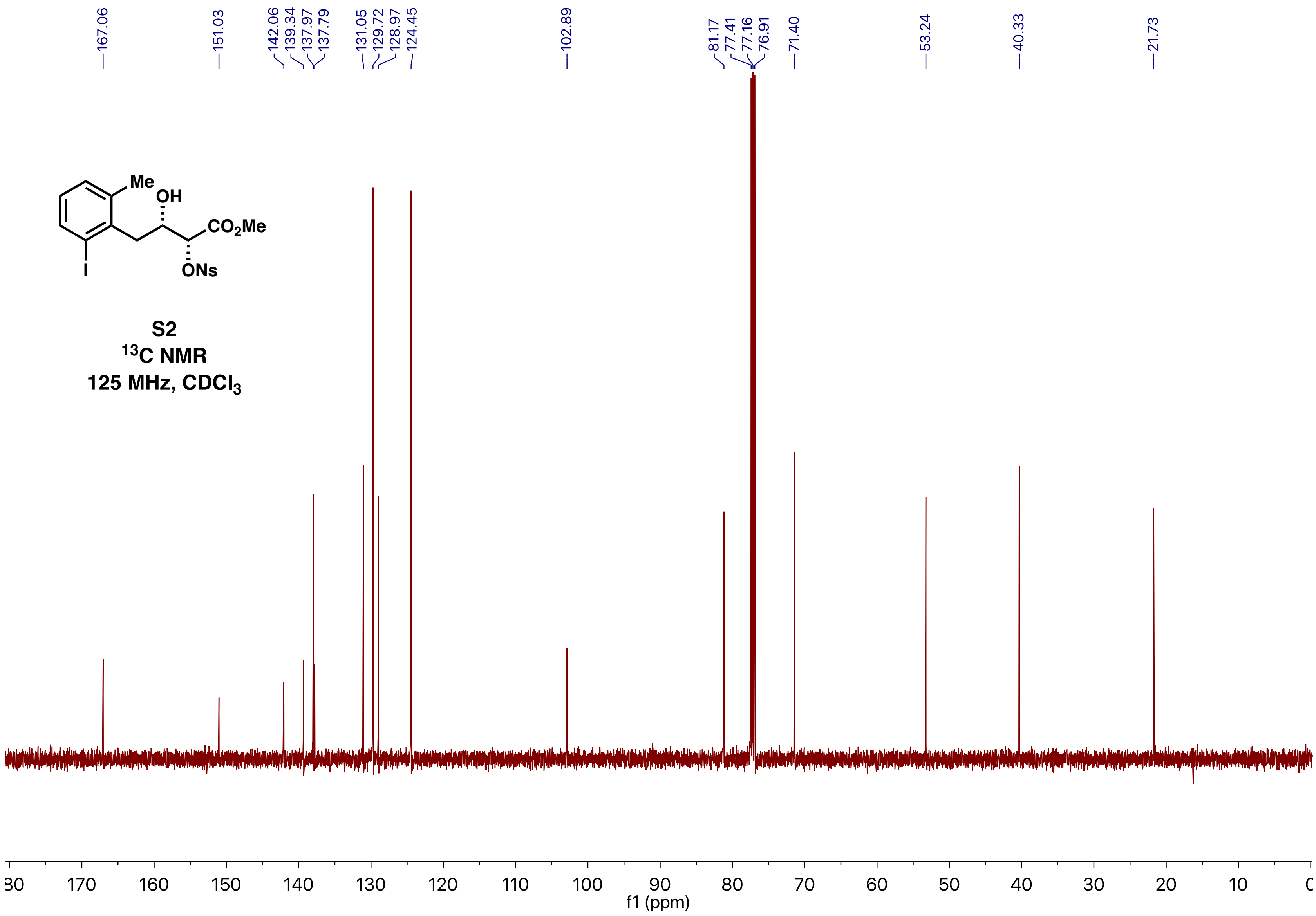
167.06
151.03
142.06
139.34
137.97
137.79
131.05
129.72
128.97
124.45
102.89
81.17
77.41
77.16
76.91
71.40
53.24
40.33
21.73
Me
OH
CO2Me
I
ONs
S2
13C NMR
125 MHz, CDCl3
f1 (ppm)

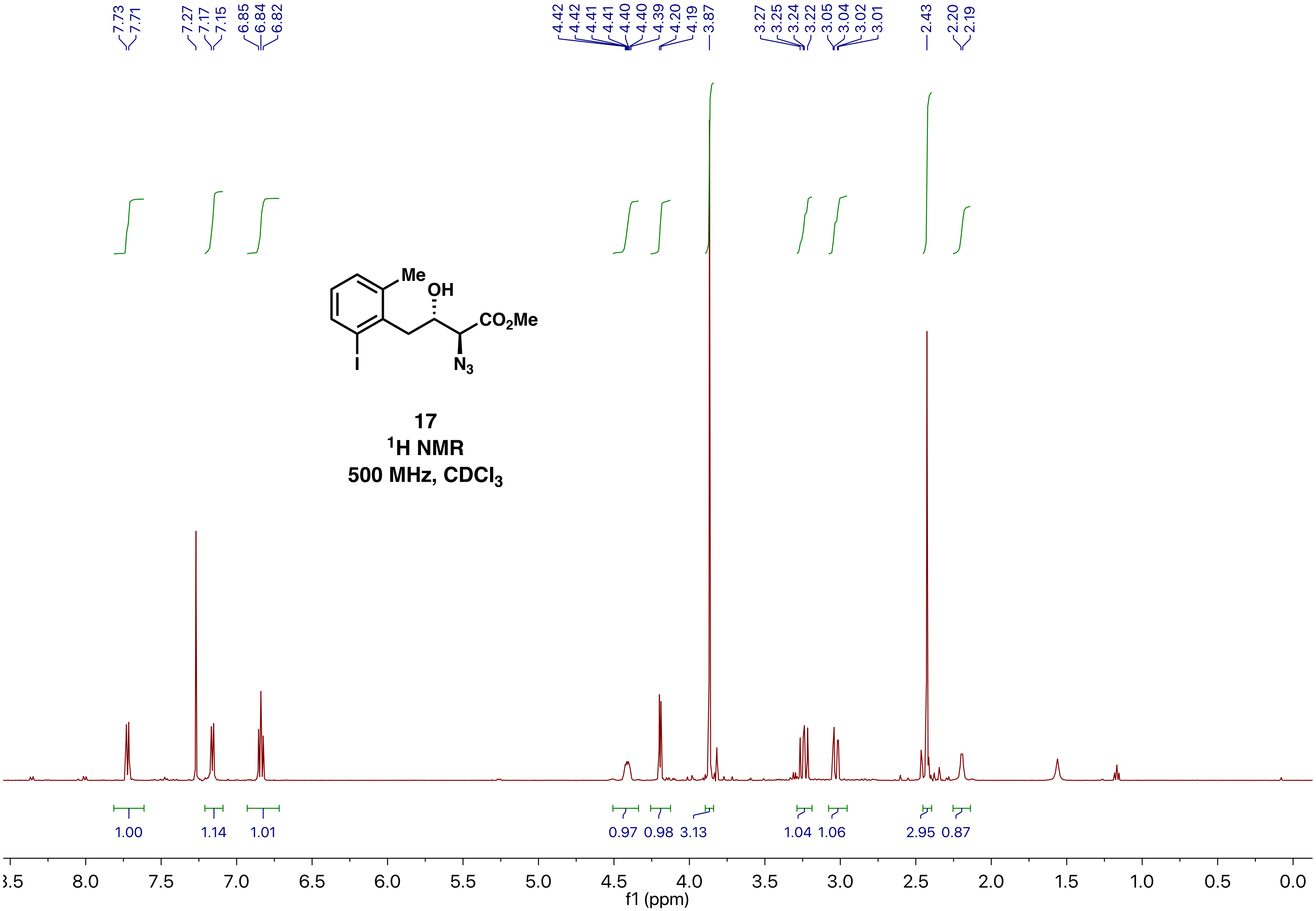
7.73
7.71
7.27
7.17
7.15
6.85
6.84
6.82
4.42
4.42
4.41
4.40
4.40
4.39
4.20
4.19
3.87
3.27
3.25
3.24
3.22
3.05
3.04
3.02
3.01
2.43
2.20
2.19
Me
OH
CO2Me
I
N3
17
1H NMR
500 MHz, CDCl3
1.00
1.14
1.01
0.97
0.98
3.13
1.04
1.06
2.95
0.87
8.0
7.5
7.0
6.5
6.0
5.5
5.0
4.5
4.0
3.5
3.0
2.5
2.0
1.5
1.0
0.5
0.0
f1 (ppm)

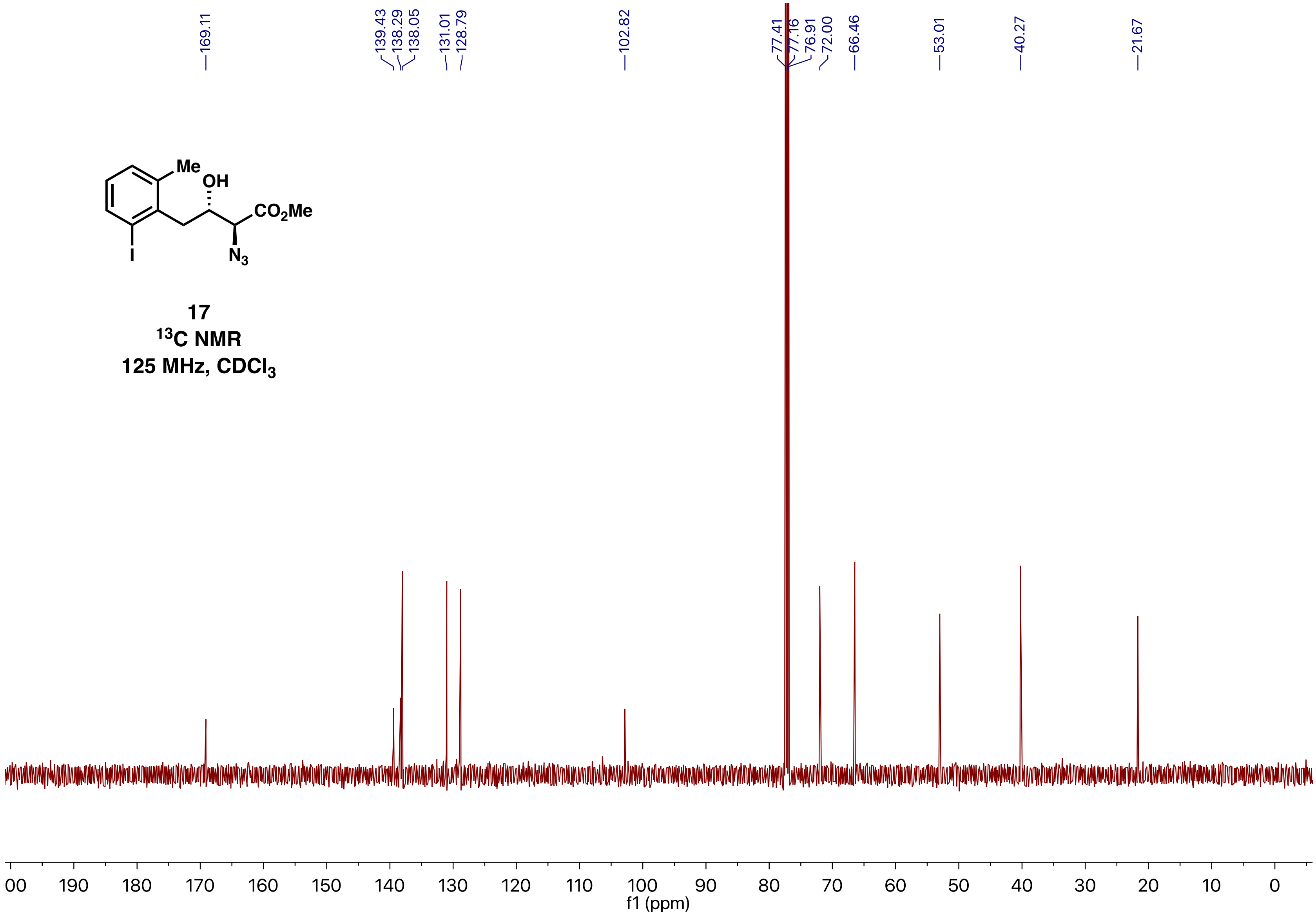

17
^{13}C NMR
125 MHz, $CDCl_3$

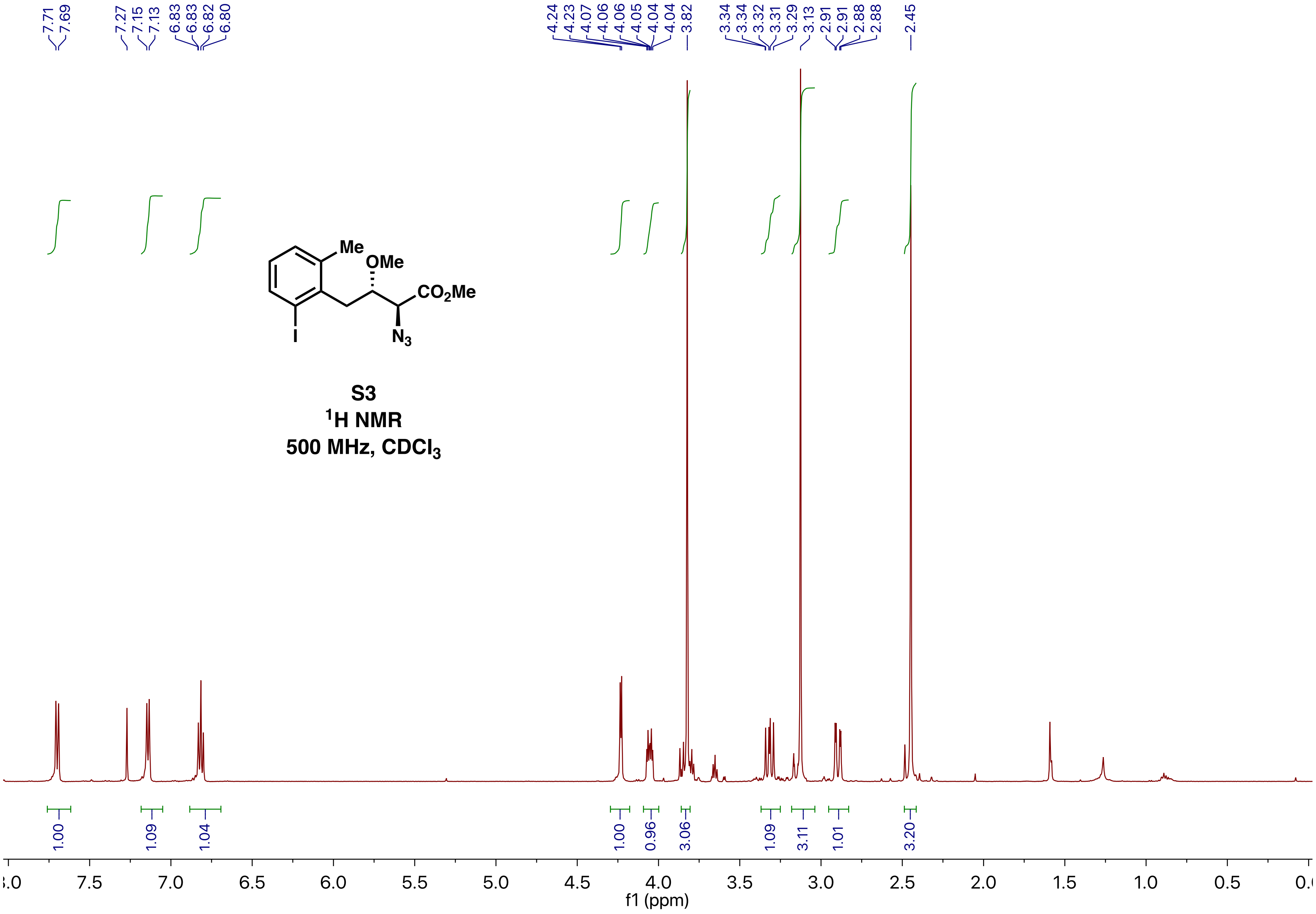
7.71
7.69
7.27
7.15
7.13
6.83
6.83
6.82
6.80
4.24
4.23
4.07
4.06
4.06
4.05
4.04
4.04
3.82
3.34
3.34
3.32
3.31
3.29
3.13
2.91
2.91
2.88
2.88
2.45
Me
OMe
CO_2Me
I
N_3
S3
^1H NMR
500 MHz, $CDCl_3$
1.00
1.09
1.04
1.00
0.96
3.06
1.09
3.11
1.01
3.20
f1 (ppm)

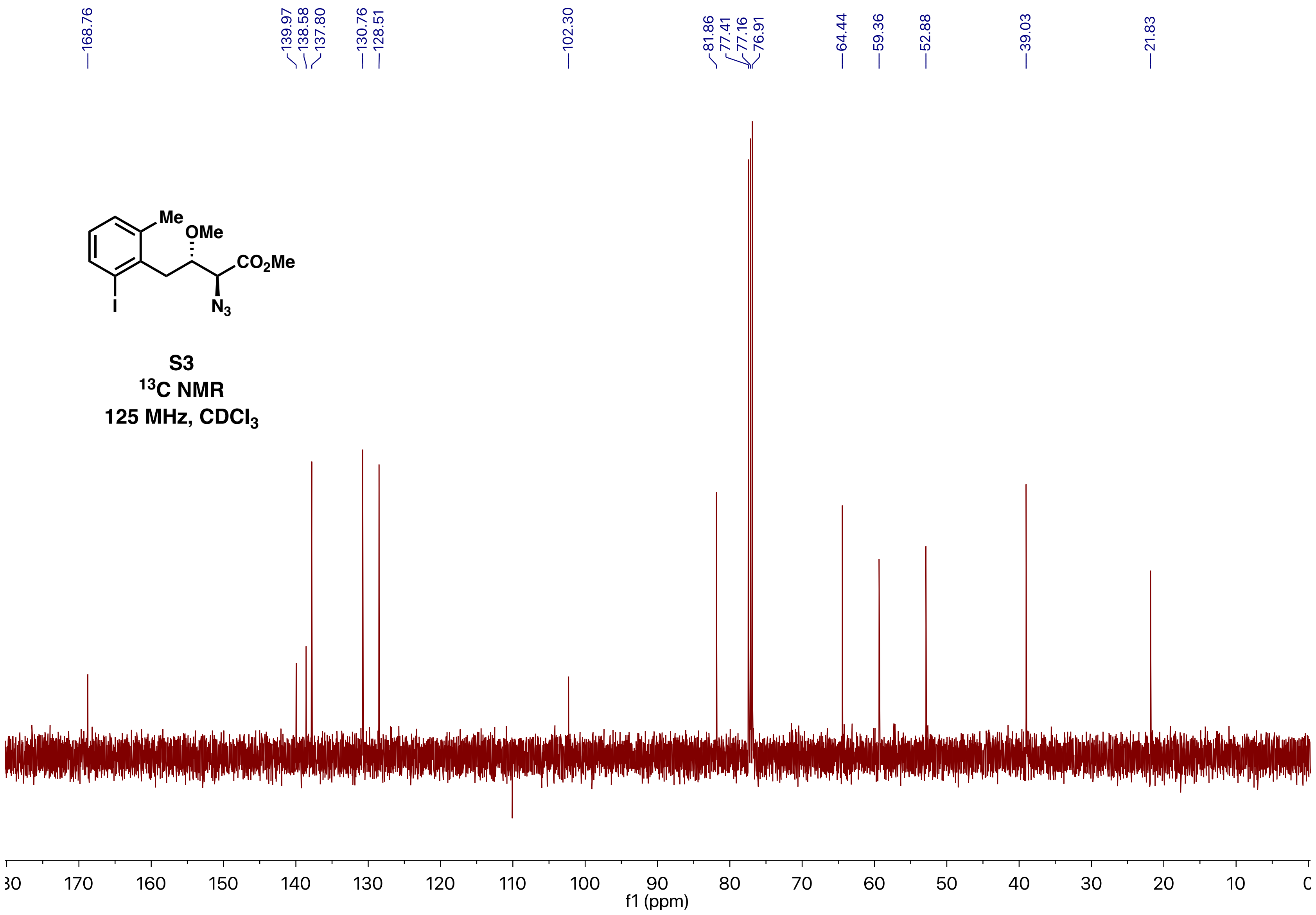
168.76
139.97
138.58
137.80
130.76
128.51
102.30
81.86
77.41
77.16
76.91
64.44
59.36
52.88
39.03
21.83
Me
OMe
CO2Me
I
N3
S3
13C NMR
125 MHz, CDCl3
f1 (ppm)

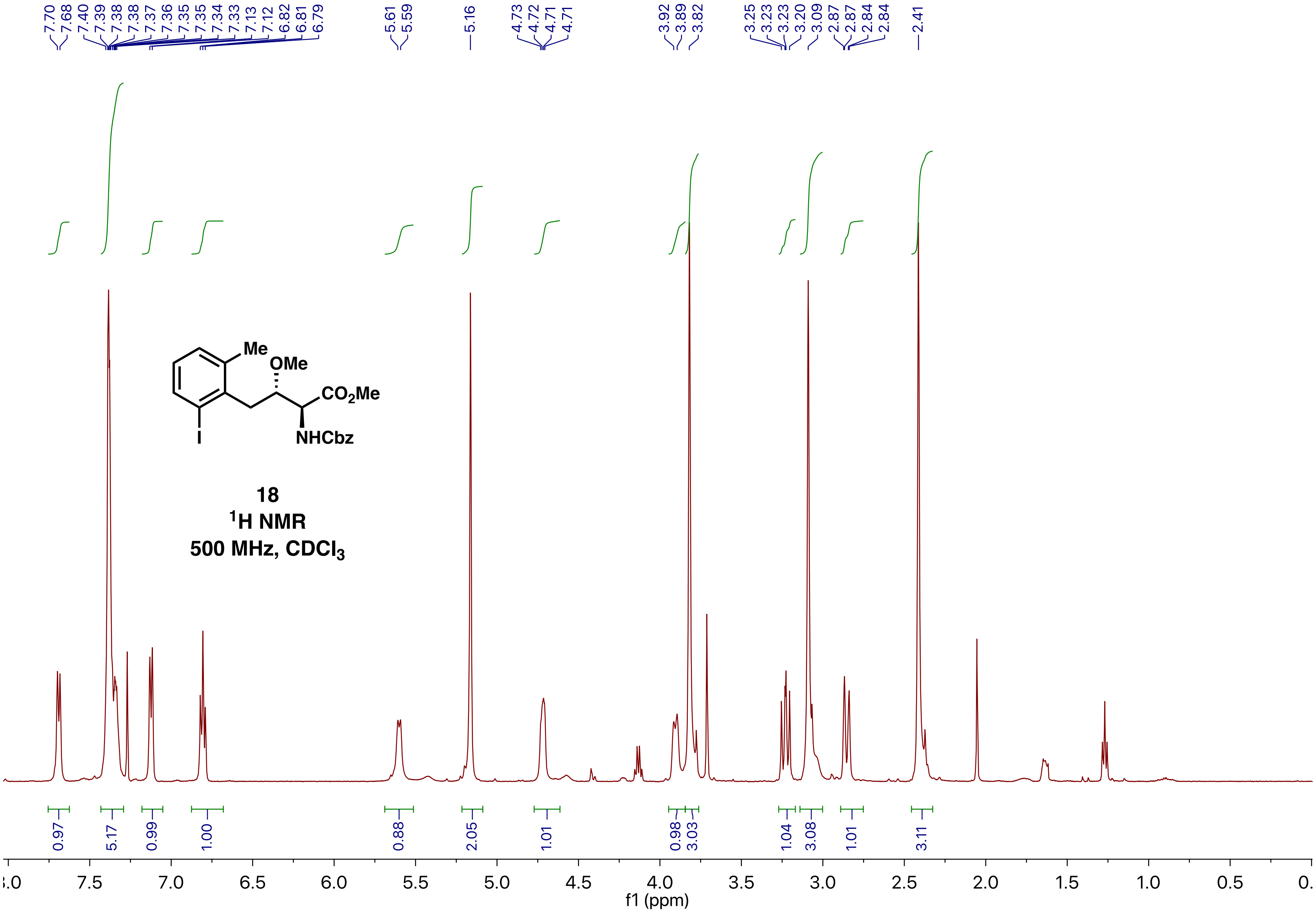

Me
OMe
CO2Me
NHCbz
I
18
1H NMR
500 MHz, CDCl3
7.70 7.68 7.40 7.39 7.38 7.38 7.37 7.36 7.35 7.35 7.34 7.33 7.13 7.12 6.82 6.81 6.79
5.61 5.59
5.16
4.73 4.72 4.71 4.71
3.92 3.89 3.82
3.25 3.23 3.23 3.20 3.09 2.87 2.87 2.84 2.84
2.41
0.97 5.17 0.99 1.00 0.88 2.05 1.01 0.98 3.03 1.04 3.08 1.01 3.11
f1 (ppm)

18
^{13}C NMR
125 MHz, $CDCl_3$

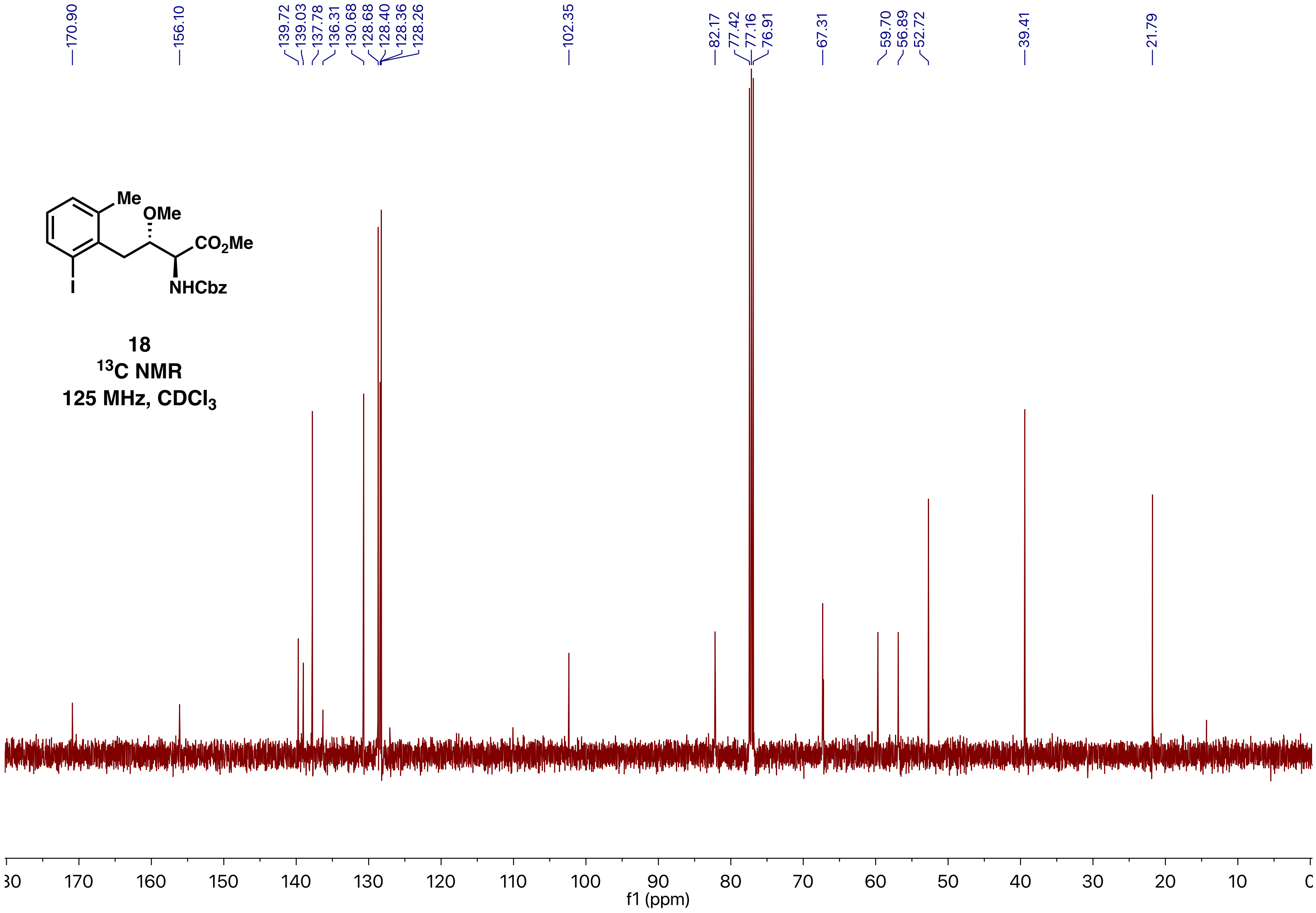

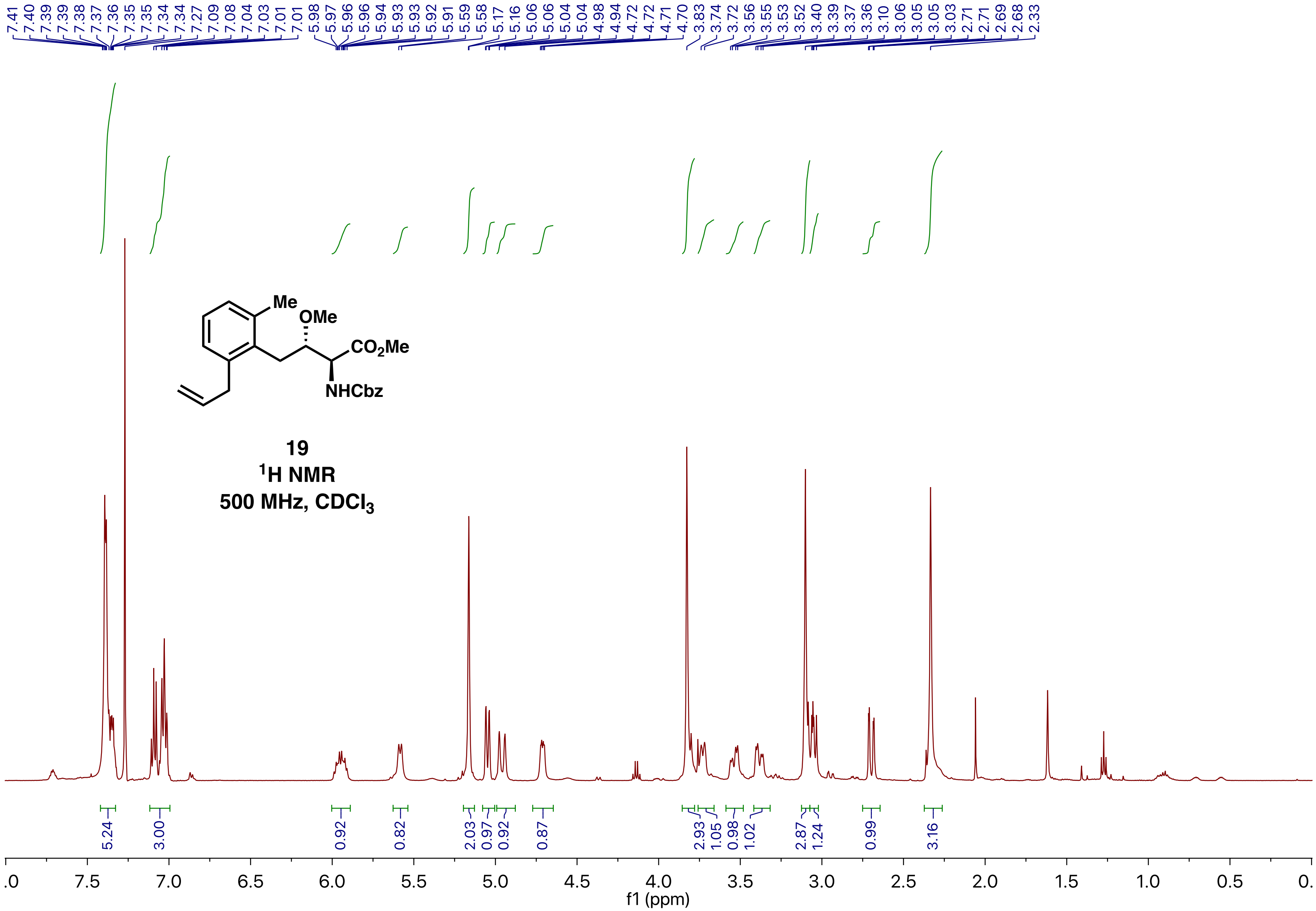
7.41 7.40 7.39 7.39 7.38 7.37 7.36 7.35 7.35 7.34 7.34 7.27 7.09 7.08 7.04 7.04 7.03 7.01 7.01 5.98 5.97 5.96 5.96 5.94 5.93 5.93 5.92 5.91 5.59 5.58 5.17 5.16 5.06 5.06 5.04 5.04 4.98 4.94 4.72 4.72 4.71 4.70 3.83 3.74 3.72 3.56 3.55 3.53 3.52 3.40 3.39 3.37 3.36 3.10 3.06 3.05 3.05 3.03 2.71 2.71 2.69 2.68 2.33
Me
OMe
CO_2Me
NHCbz
19
^1H NMR
500 MHz, $CDCl_3$
5.24 3.00 0.92 0.82 2.03 0.97 0.92 0.87 2.93 1.05 0.98 1.02 2.87 1.24 0.99 3.16
.0 7.5 7.0 6.5 6.0 5.5 5.0 4.5 4.0 3.5 3.0 2.5 2.0 1.5 1.0 0.5 0.
f1 (ppm)

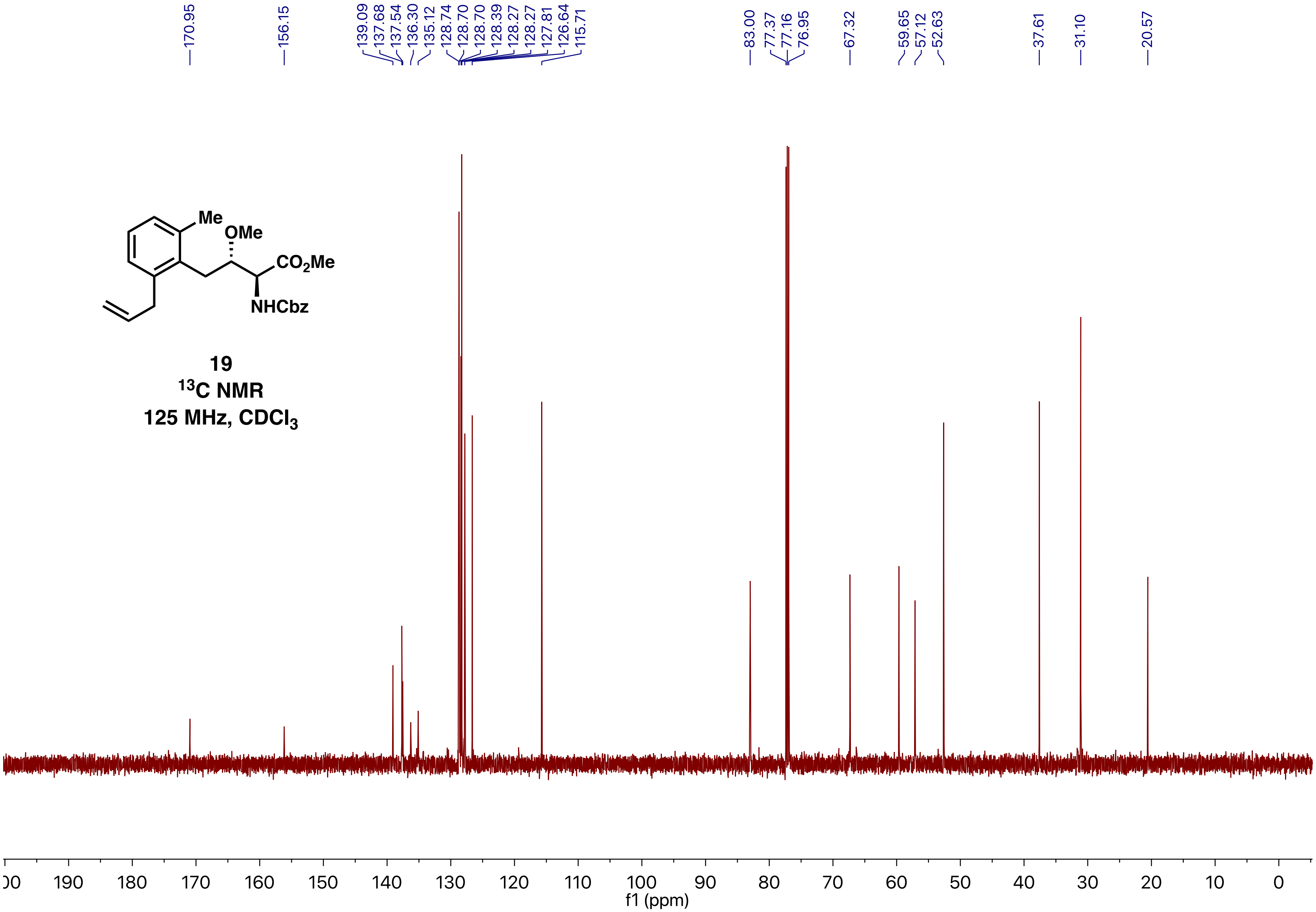

19
^{13}C NMR
125 MHz, $CDCl_3$

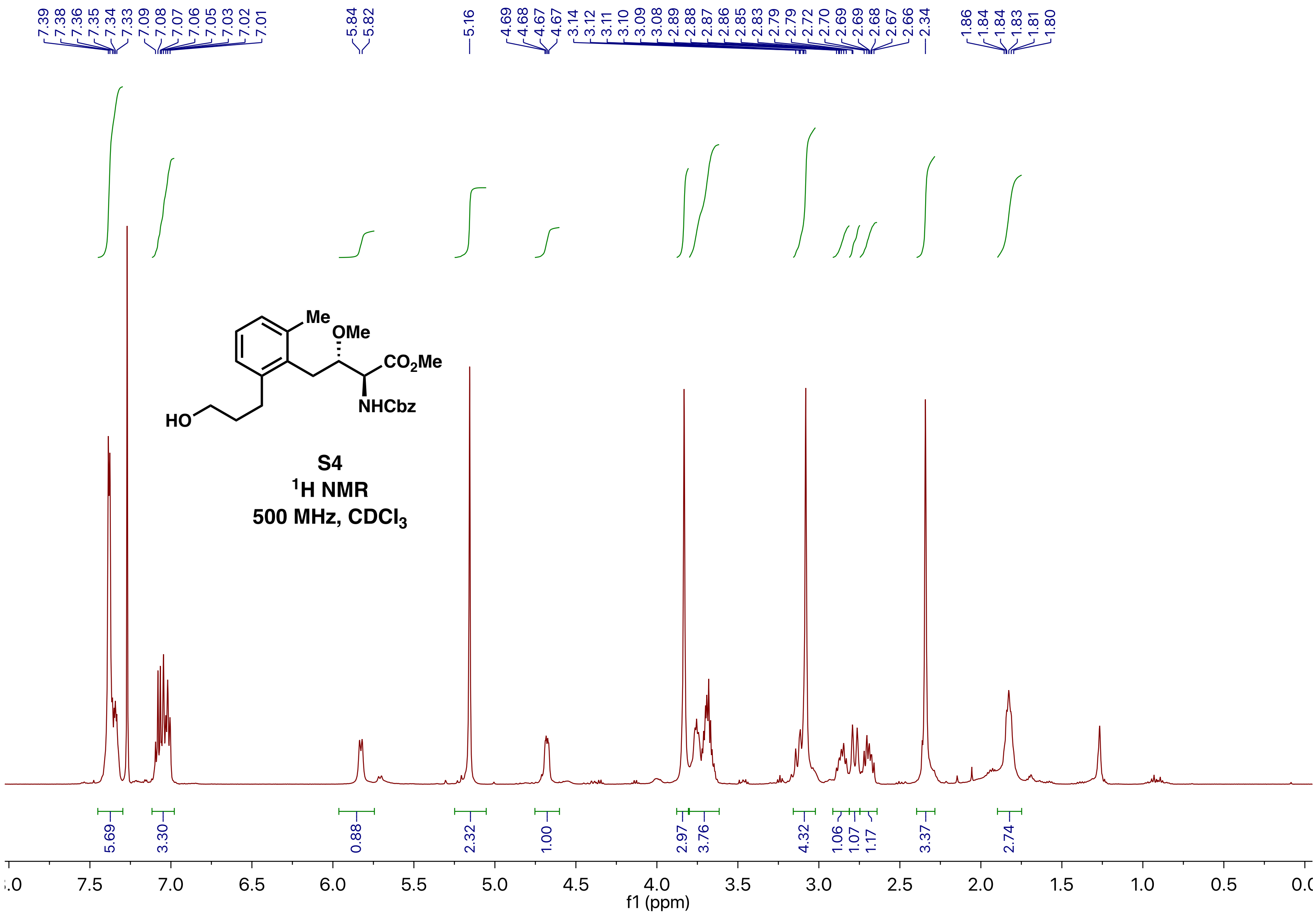
Me
OMe
CO2Me
NHCbz
HO
S4
1H NMR
500 MHz, CDCl3
f1 (ppm)

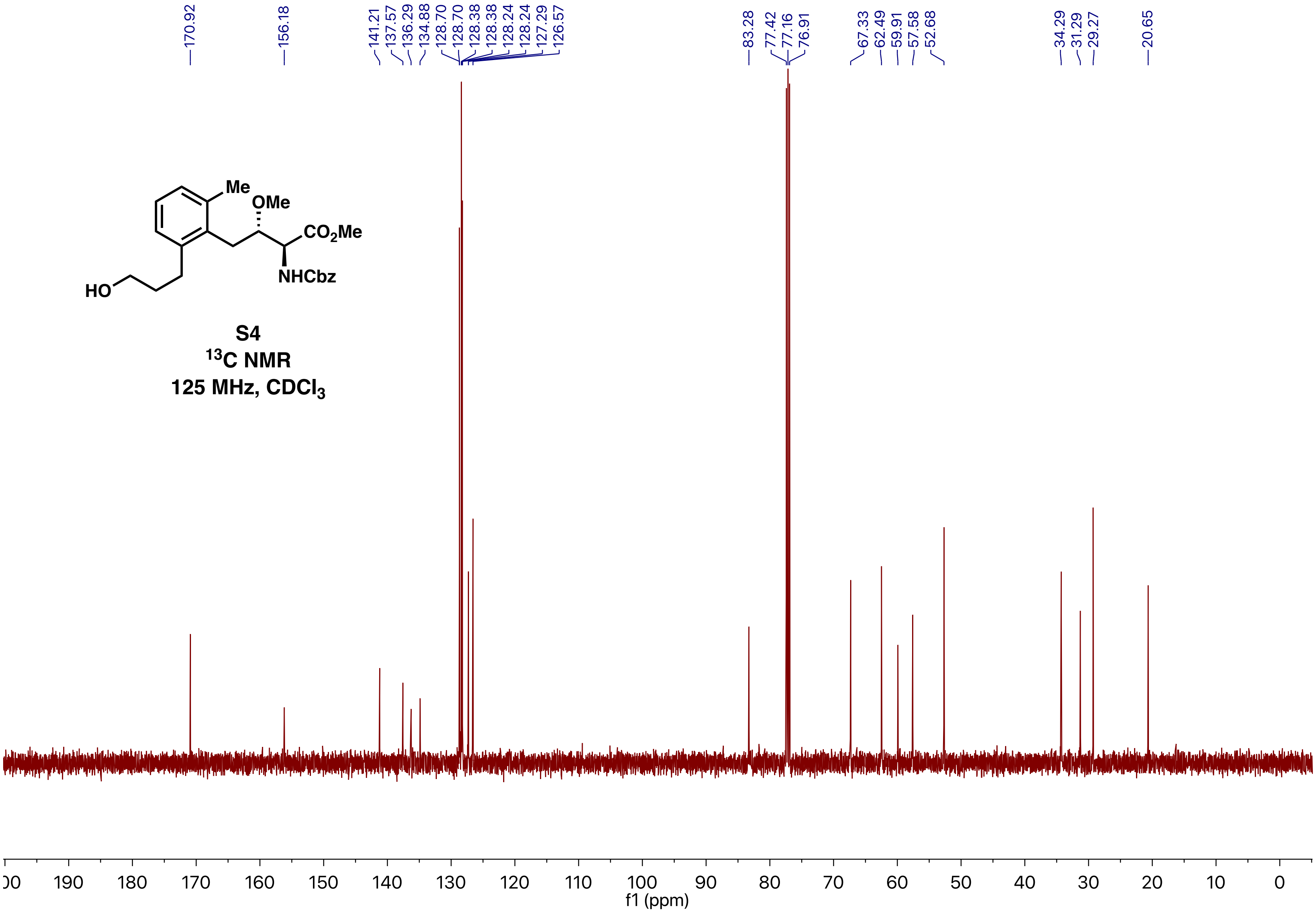

Me
OMe
CO_2Me
NHCbz
HO
S4
^{13}C NMR
125 MHz, $CDCl_3$
170.92
156.18
141.21
137.57
136.29
134.88
128.70
128.70
128.38
128.38
128.24
128.24
127.29
126.57
83.28
77.42
77.16
76.91
67.33
62.49
59.91
57.58
52.68
34.29
31.29
29.27
20.65
f1 (ppm)

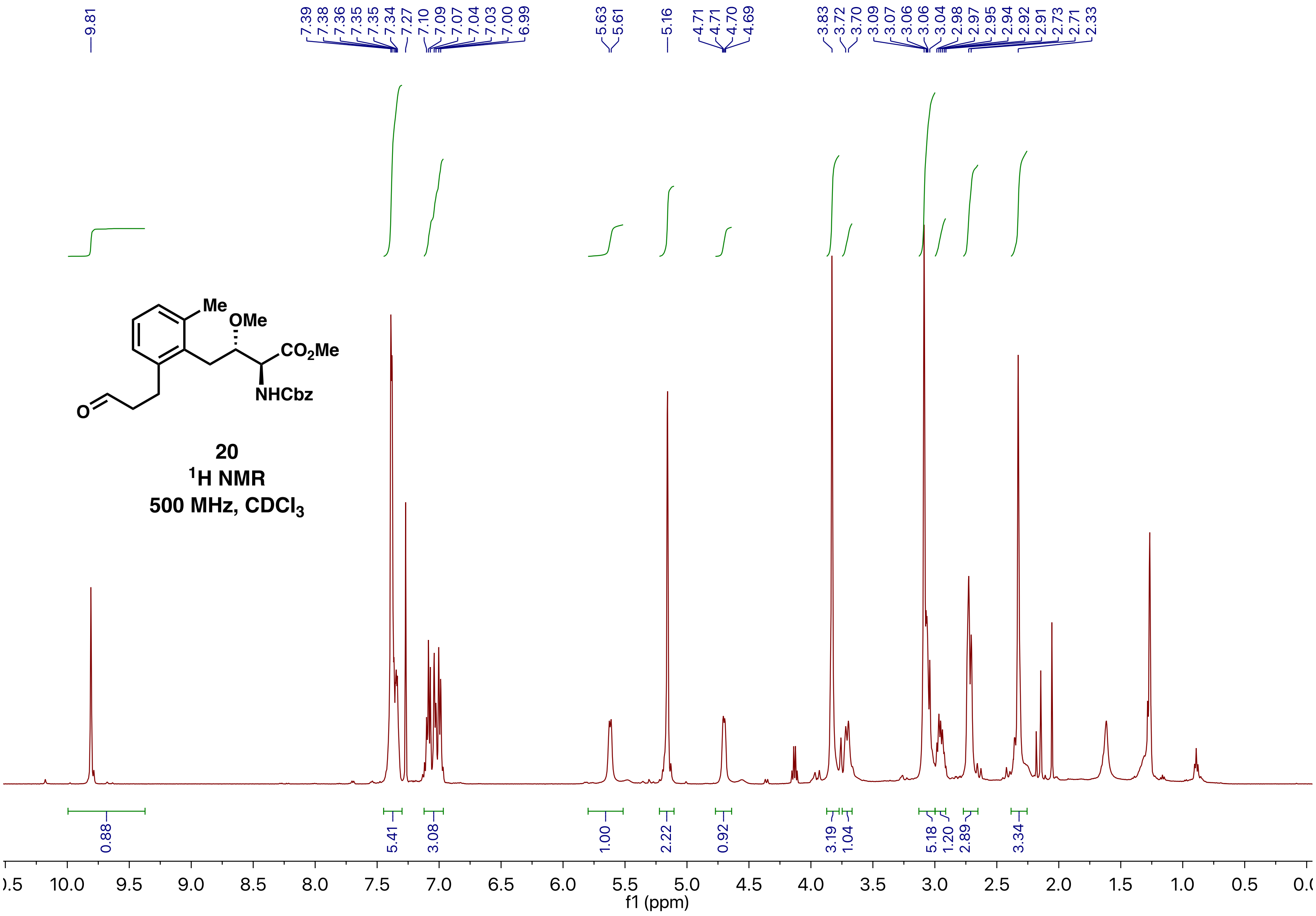

Me
OMe
CO_2Me
NHCbz
O
20
^1H NMR
500 MHz, $CDCl_3$
f1 (ppm)

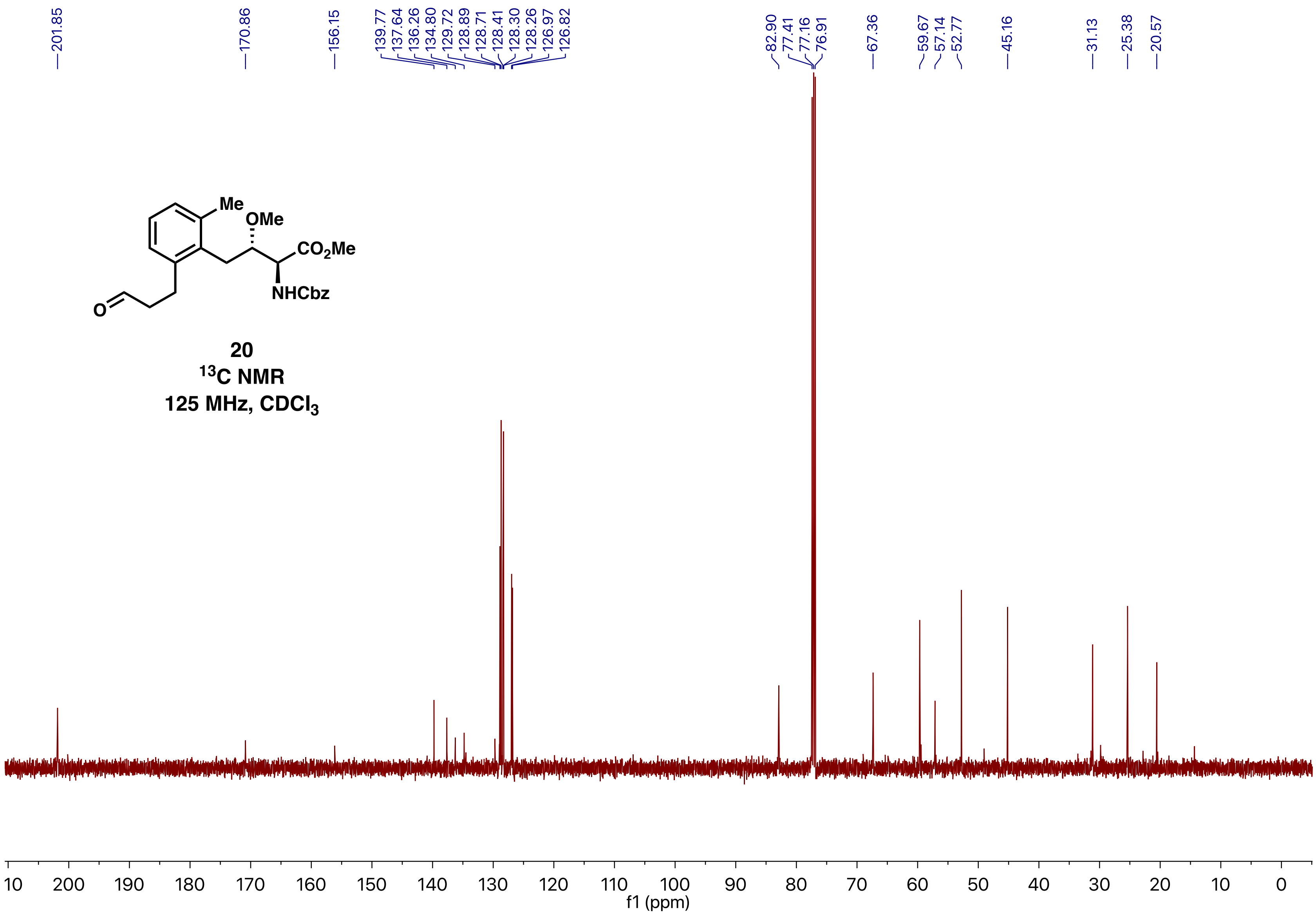

201.85
170.86
156.15
139.77
137.64
136.26
134.80
129.72
128.89
128.71
128.41
128.30
128.26
126.97
126.82
82.90
77.41
77.16
76.91
67.36
59.67
57.14
52.77
45.16
31.13
25.38
20.57
Me
OMe
CO2Me
NHCbz
O
20
13C NMR
125 MHz, CDCl3
f1 (ppm)

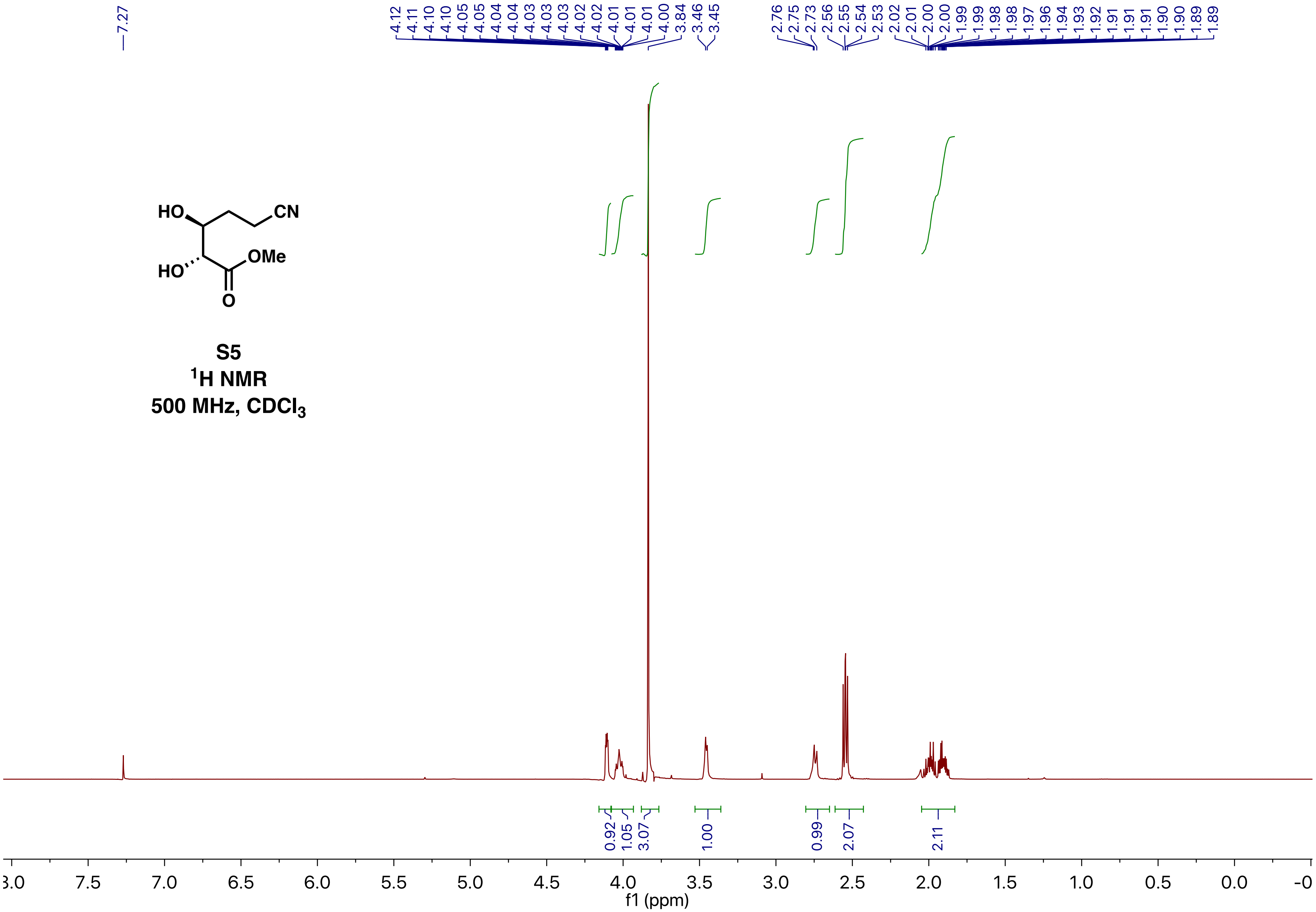
7.27
4.12 4.11 4.10 4.10 4.05 4.05 4.04 4.04 4.03 4.03 4.03 4.02 4.02 4.01 4.01 4.01 4.00 3.84 3.46 3.45
2.76 2.75 2.73 2.56 2.55 2.54 2.53 2.02 2.01 2.00 2.00 1.99 1.99 1.98 1.98 1.97 1.96 1.94 1.93 1.92 1.91 1.91 1.91 1.90 1.90 1.89 1.89
HO
CN
OMe
HO
O
S5
1H NMR
500 MHz, CDCl3
0.92
1.05
3.07
1.00
0.99
2.07
2.11
f1 (ppm)

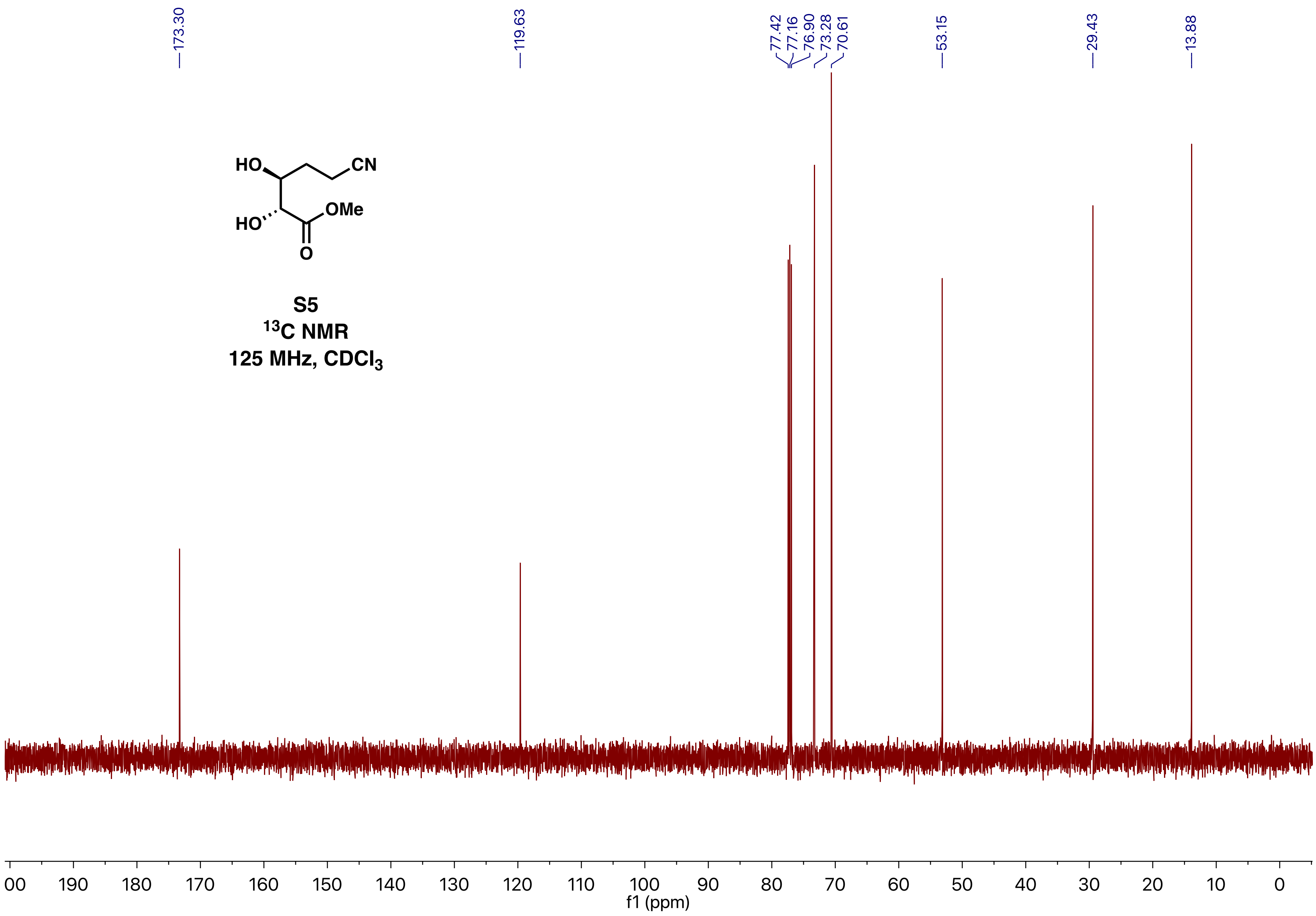

173.30
119.63
77.42
77.16
76.90
73.28
70.61
53.15
29.43
13.88
HO
CN
OMe
HO
O
S5
^{13}C NMR
125 MHz, CDCl$_3$
00
190
180
170
160
150
140
130
120
110
100
90
80
70
60
50
40
30
20
10
0
f1 (ppm)

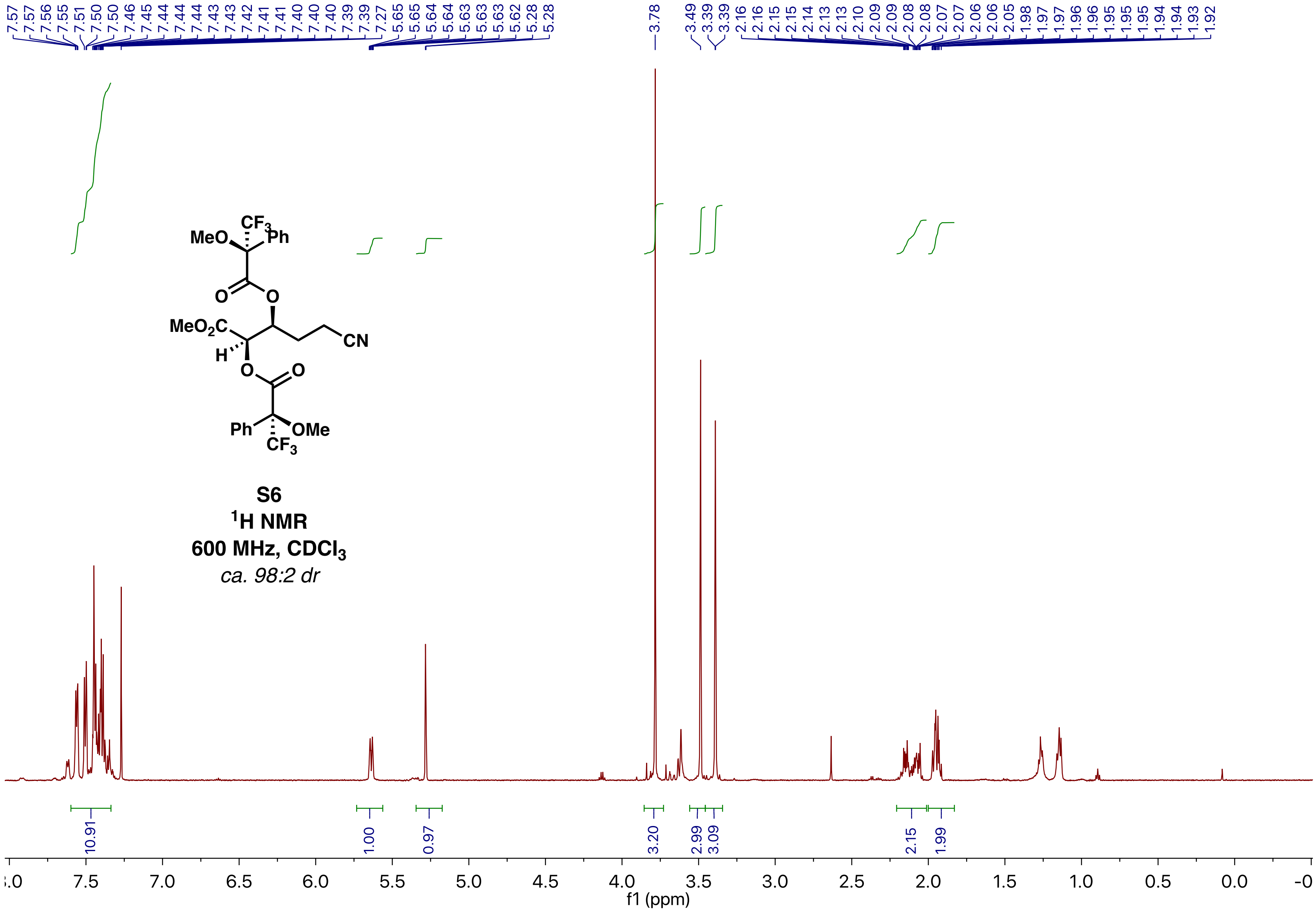
S6
^{1}H NMR
600 MHz, $CDCl_3$
ca. 98:2 dr
f1 (ppm)

S6
^{13}C NMR
150 MHz, $CDCl_3$
ca. 98:2 dr

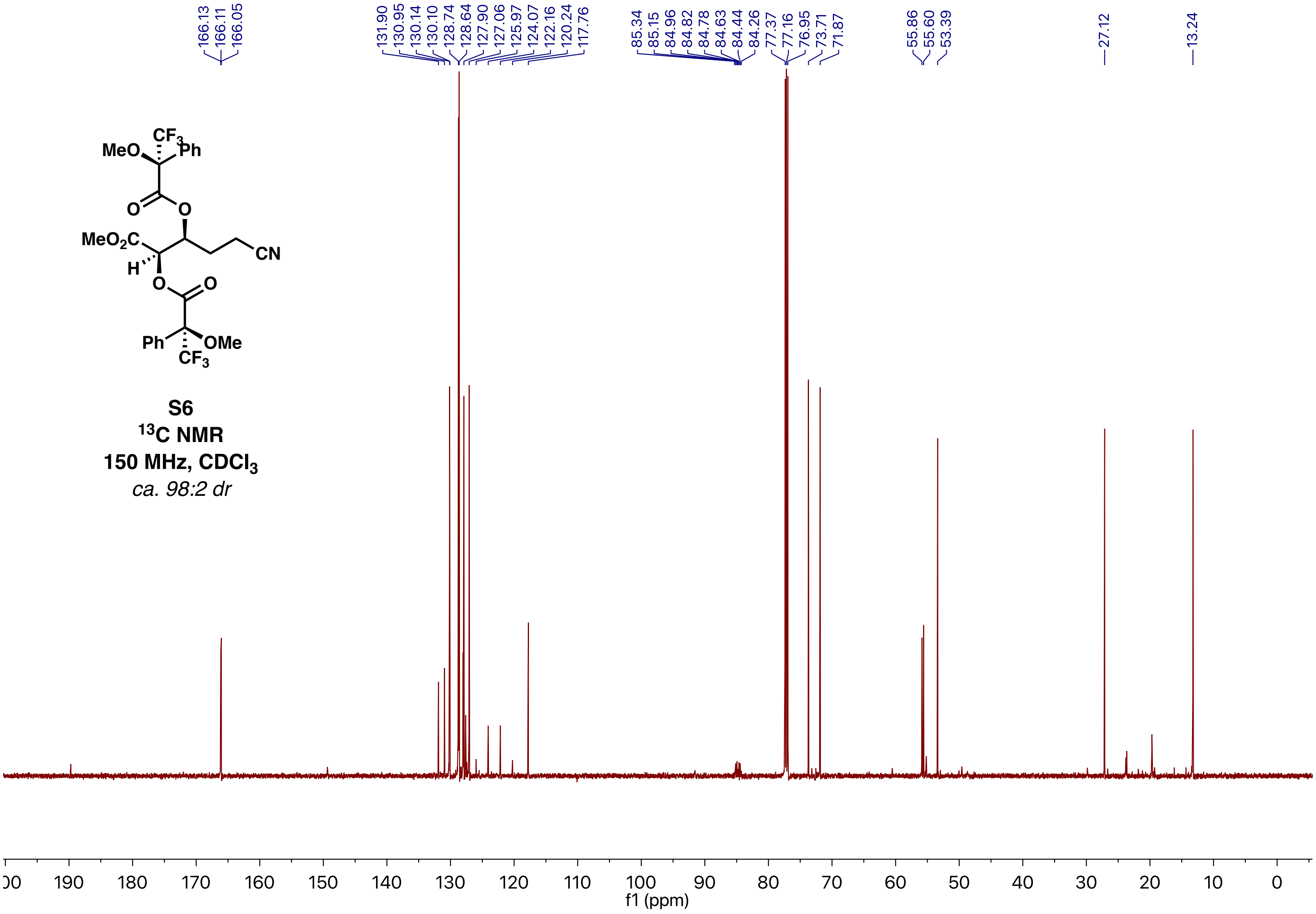

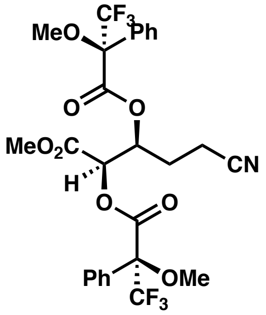

S6

^{19}F NMR

376 MHz, $CDCl_3$

ca. 98:2 dr

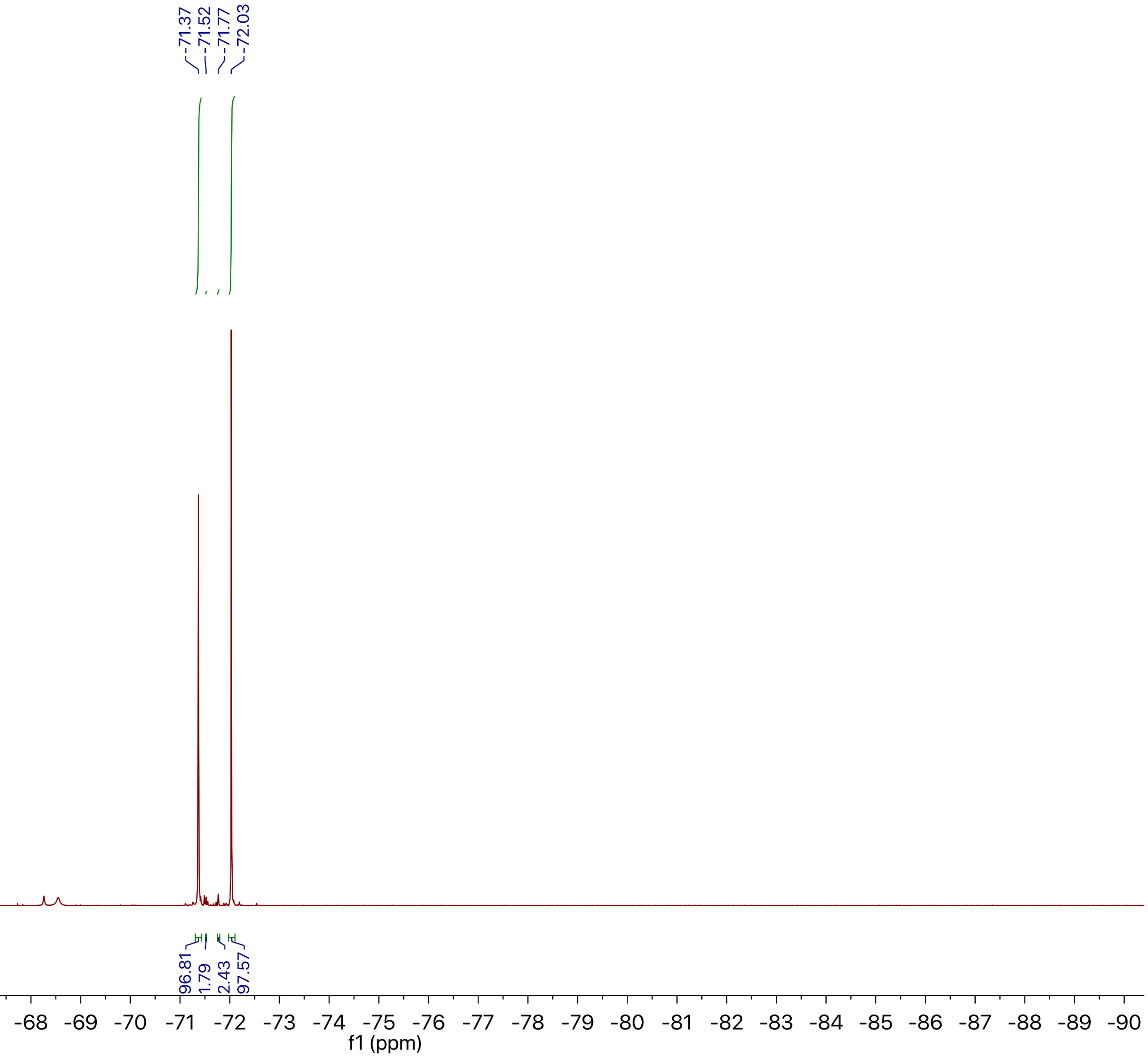

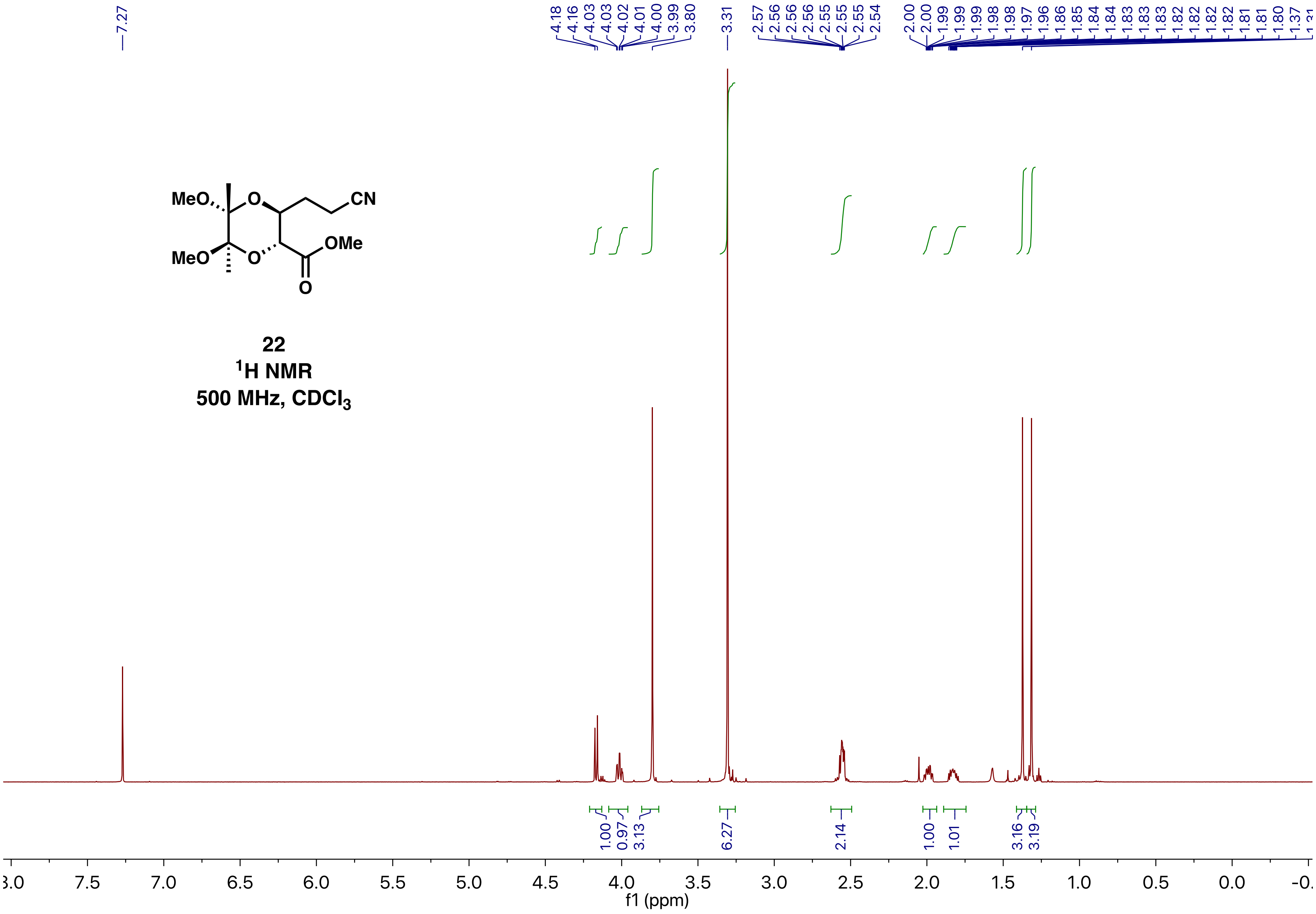

22

^{1}H NMR

500 MHz, $CDCl_3$

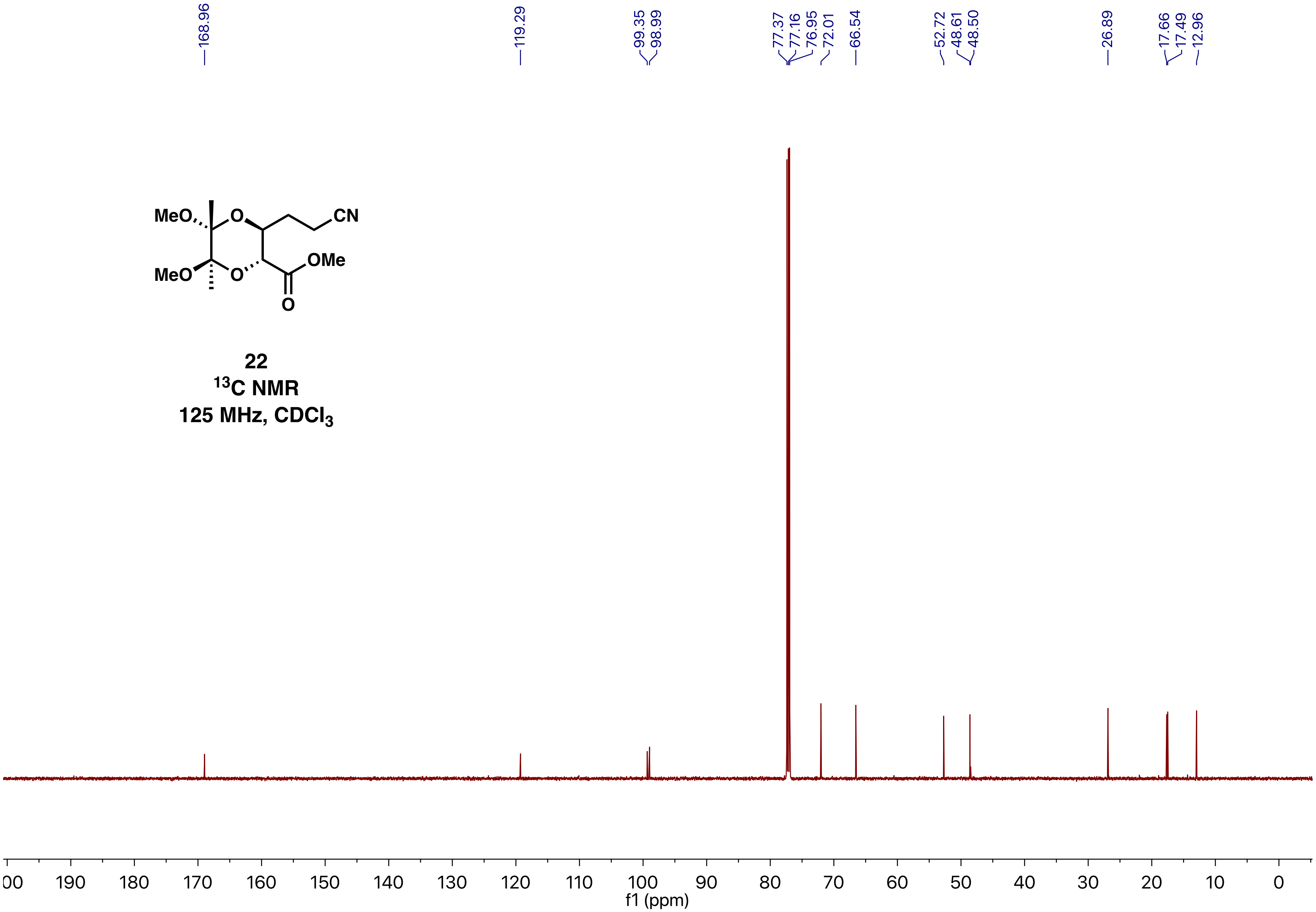
168.96
119.29
99.35
98.99
77.37
77.16
76.95
72.01
66.54
52.72
48.61
48.50
26.89
17.66
17.49
12.96
MeO
O
CN
OMe
MeO
O
O
22
13C NMR
125 MHz, CDCl3
00
190
180
170
160
150
140
130
120
110
100
90
80
70
60
50
40
30
20
10
0
f1 (ppm)

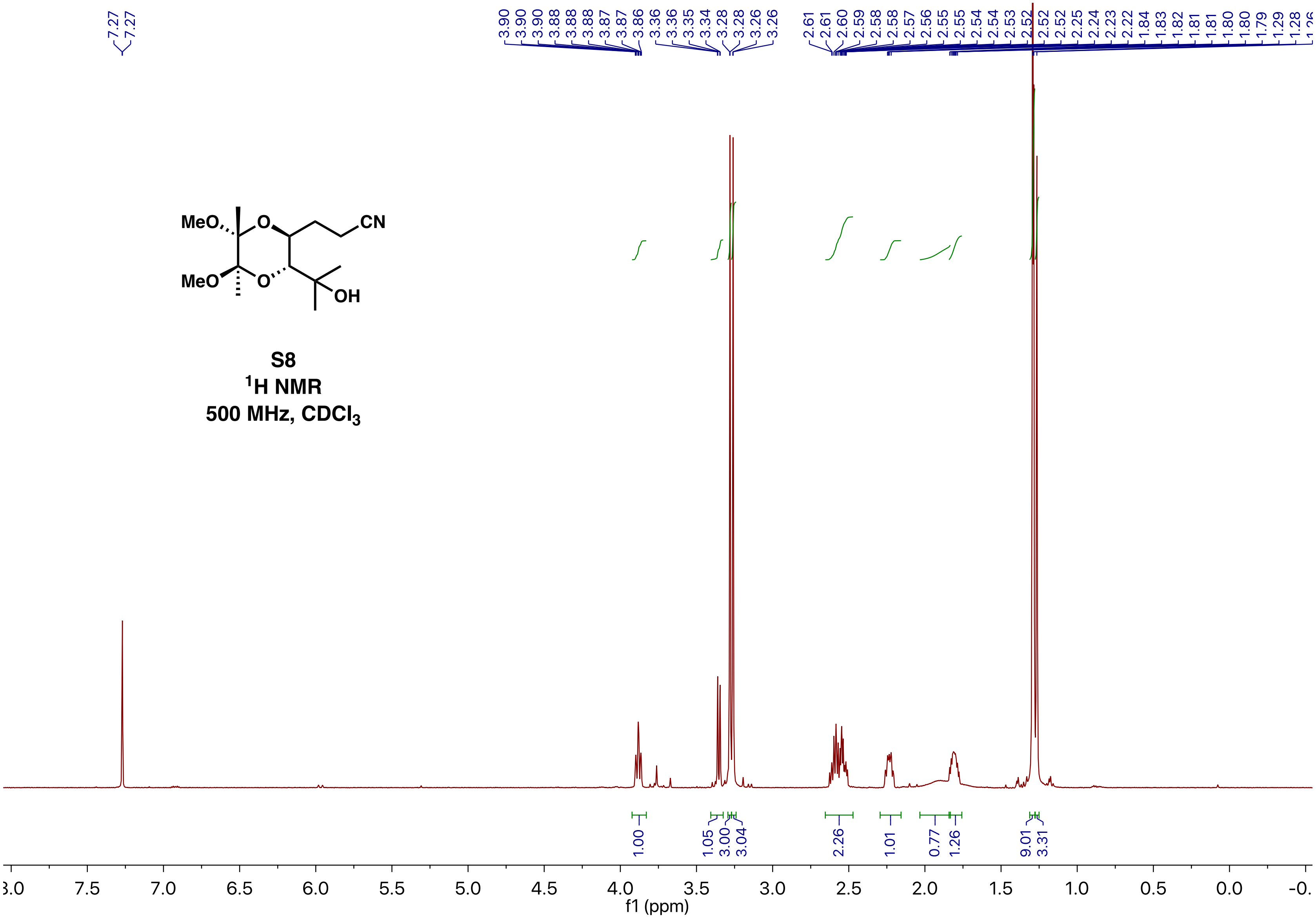

S8
^{1}H NMR
500 MHz, $CDCl_3$

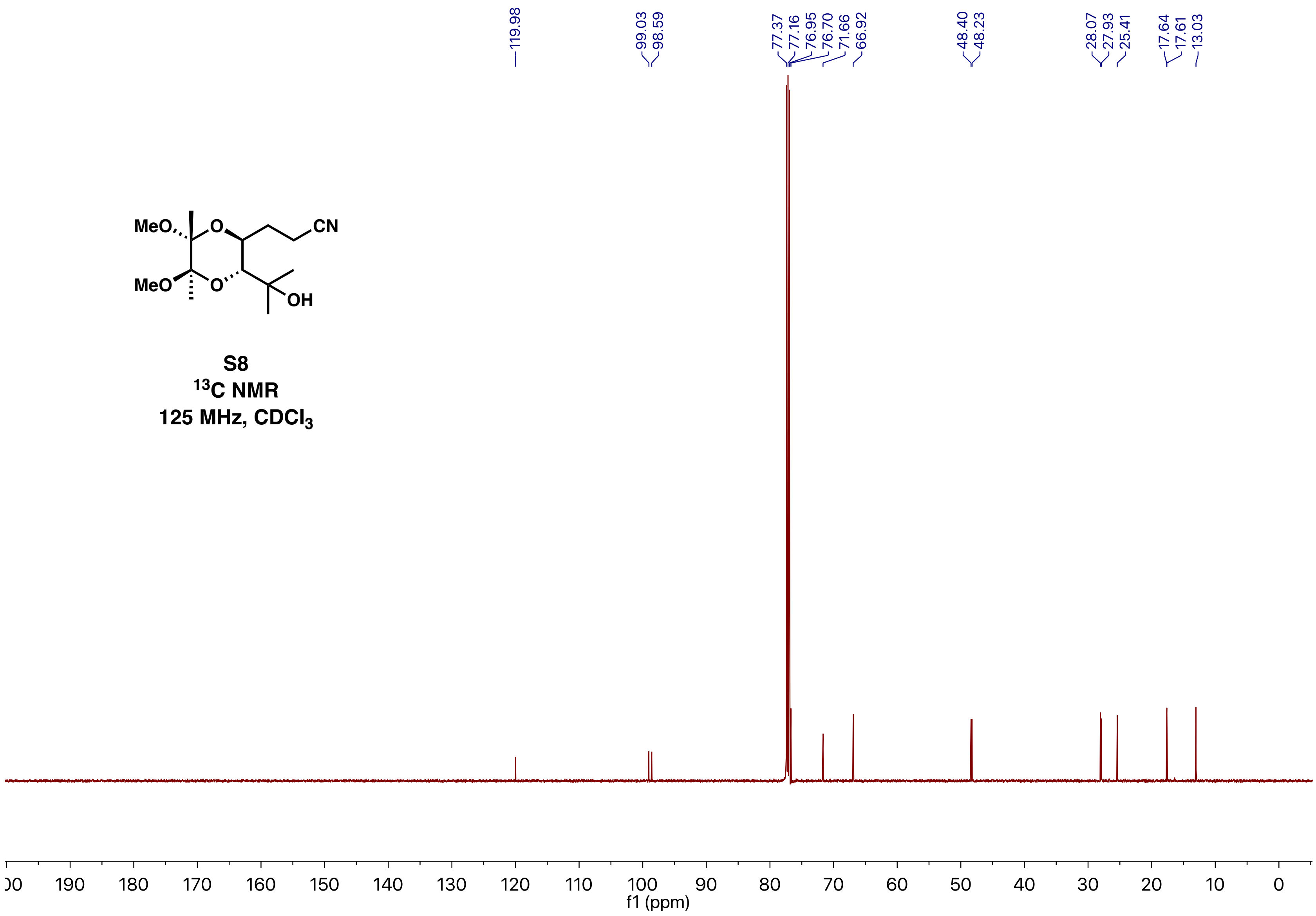

MeO
O
CN
MeO
O
OH
S8
^{13}C NMR
125 MHz, CDCl$_3$
119.98
99.03
98.59
77.37
77.16
76.95
76.70
71.66
66.92
48.40
48.23
28.07
27.93
25.41
17.64
17.61
13.03
f1 (ppm)

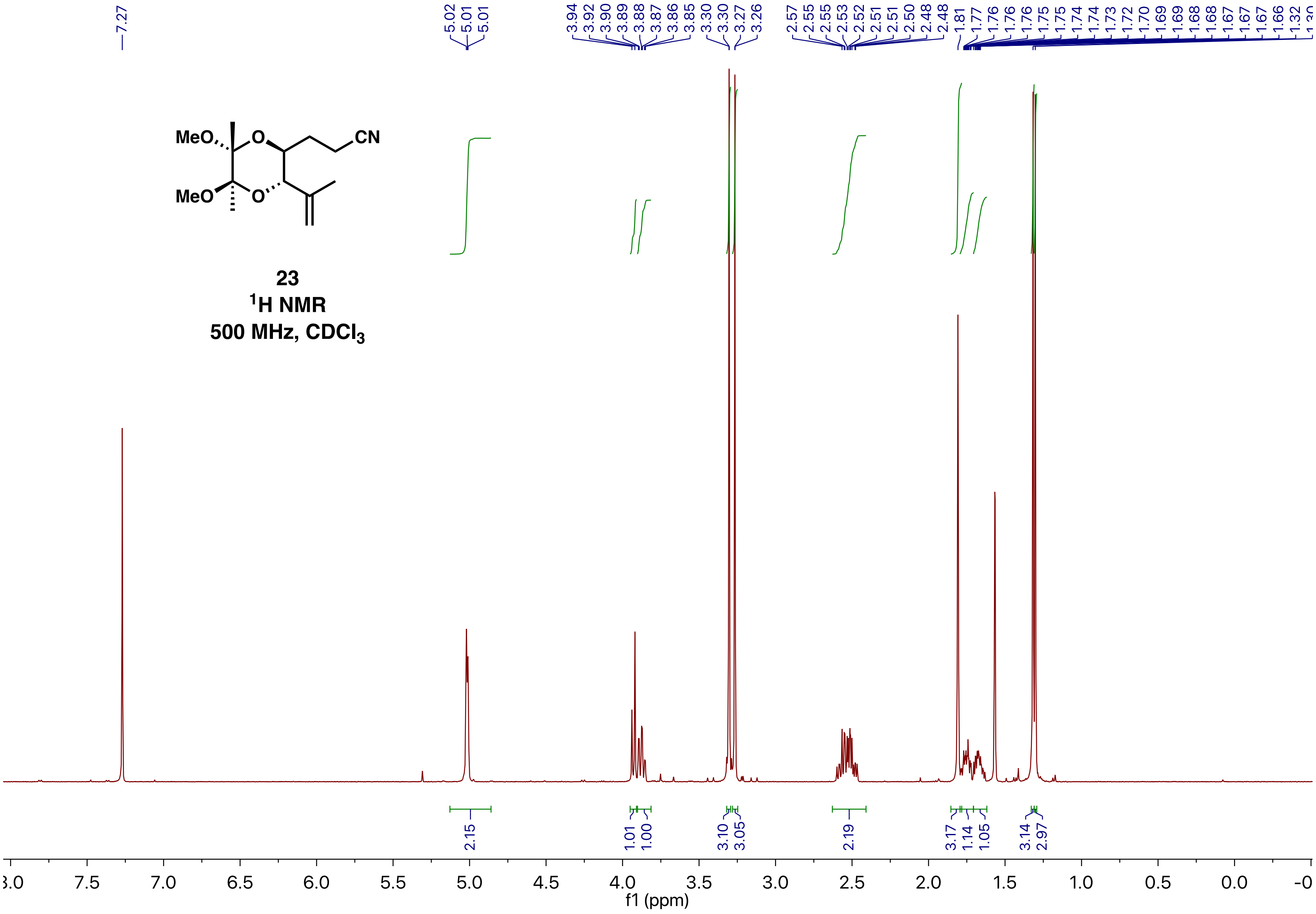

23

^{1}H NMR

500 MHz, $CDCl_3$

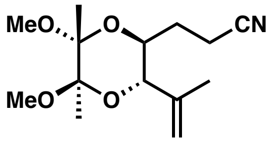

23

^{13}C NMR

125 MHz, $CDCl_3$

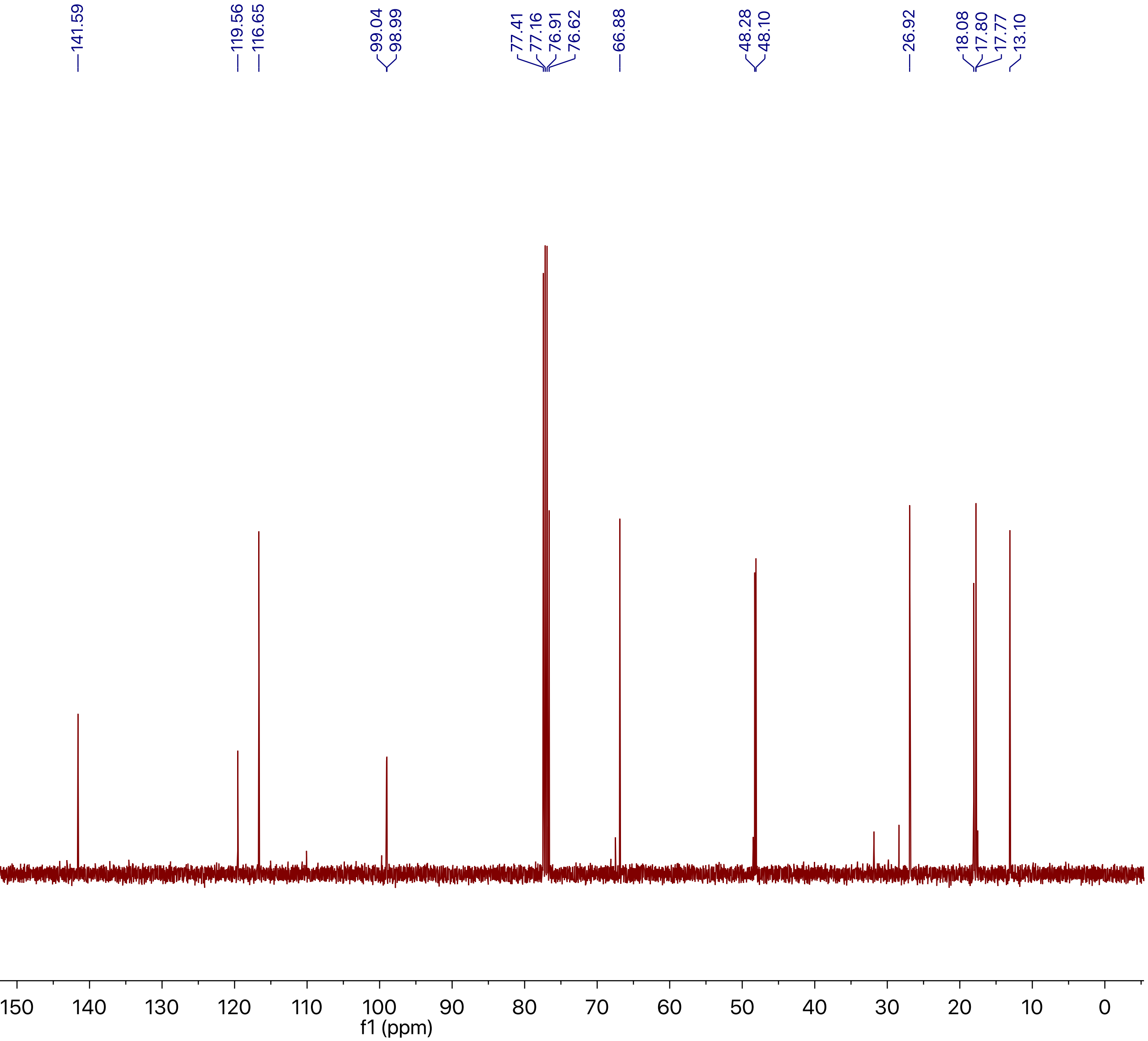

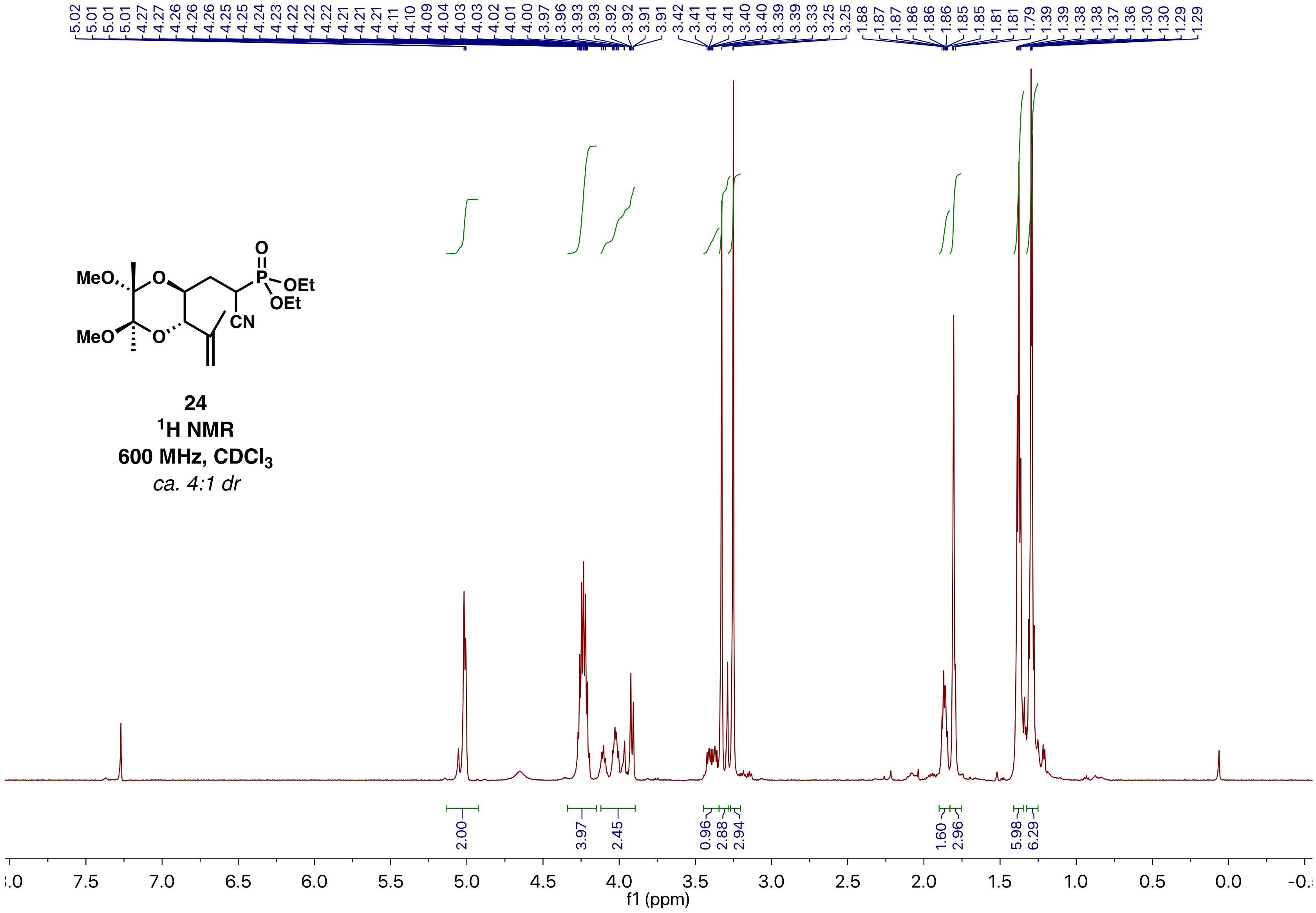
MeO
MeO
O
O
P
OEt
OEt
CN
24
1H NMR
600 MHz, CDCl3
ca. 4:1 dr
f1 (ppm)

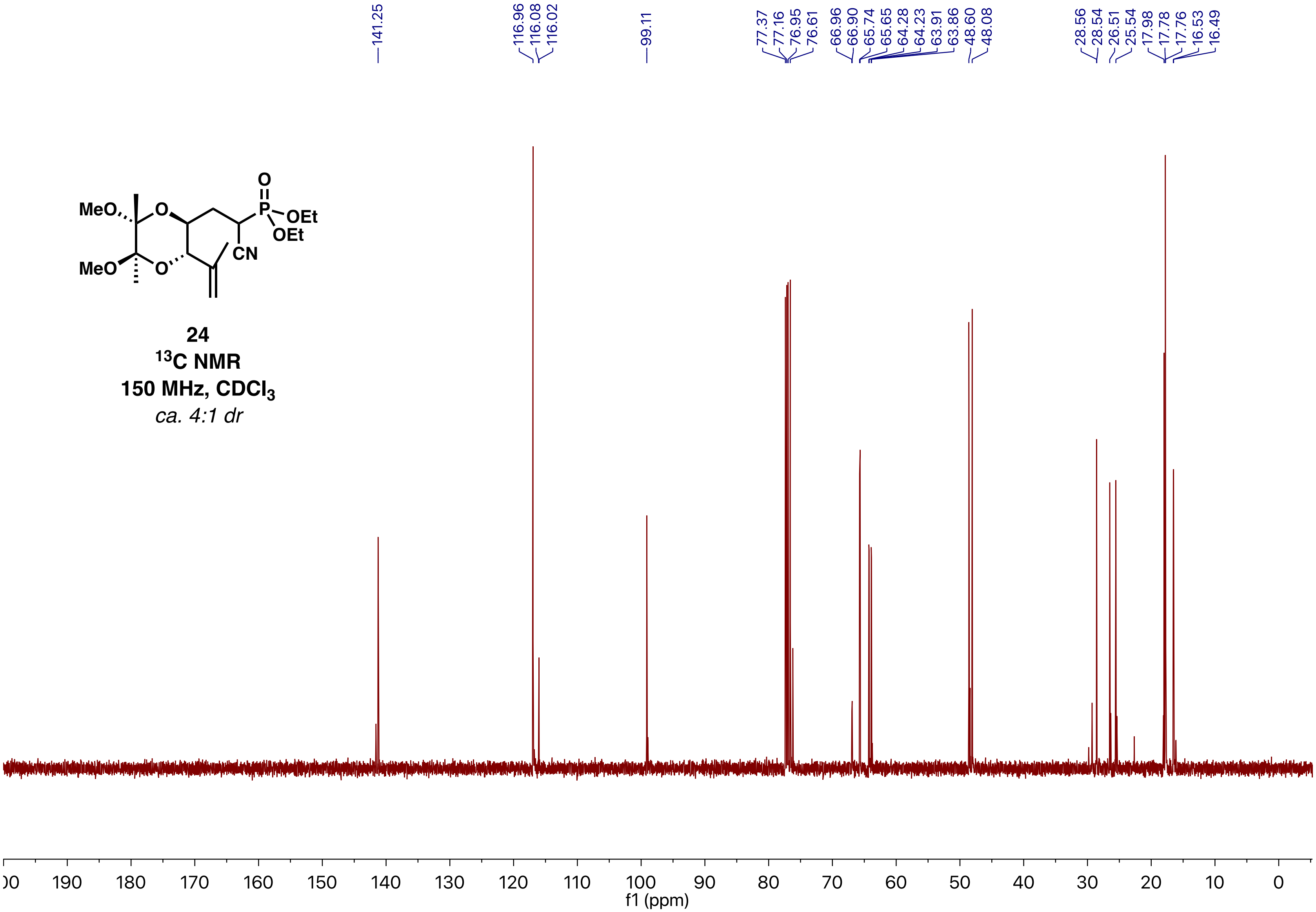

24
^{13}C NMR
150 MHz, $CDCl_3$
ca. 4:1 dr

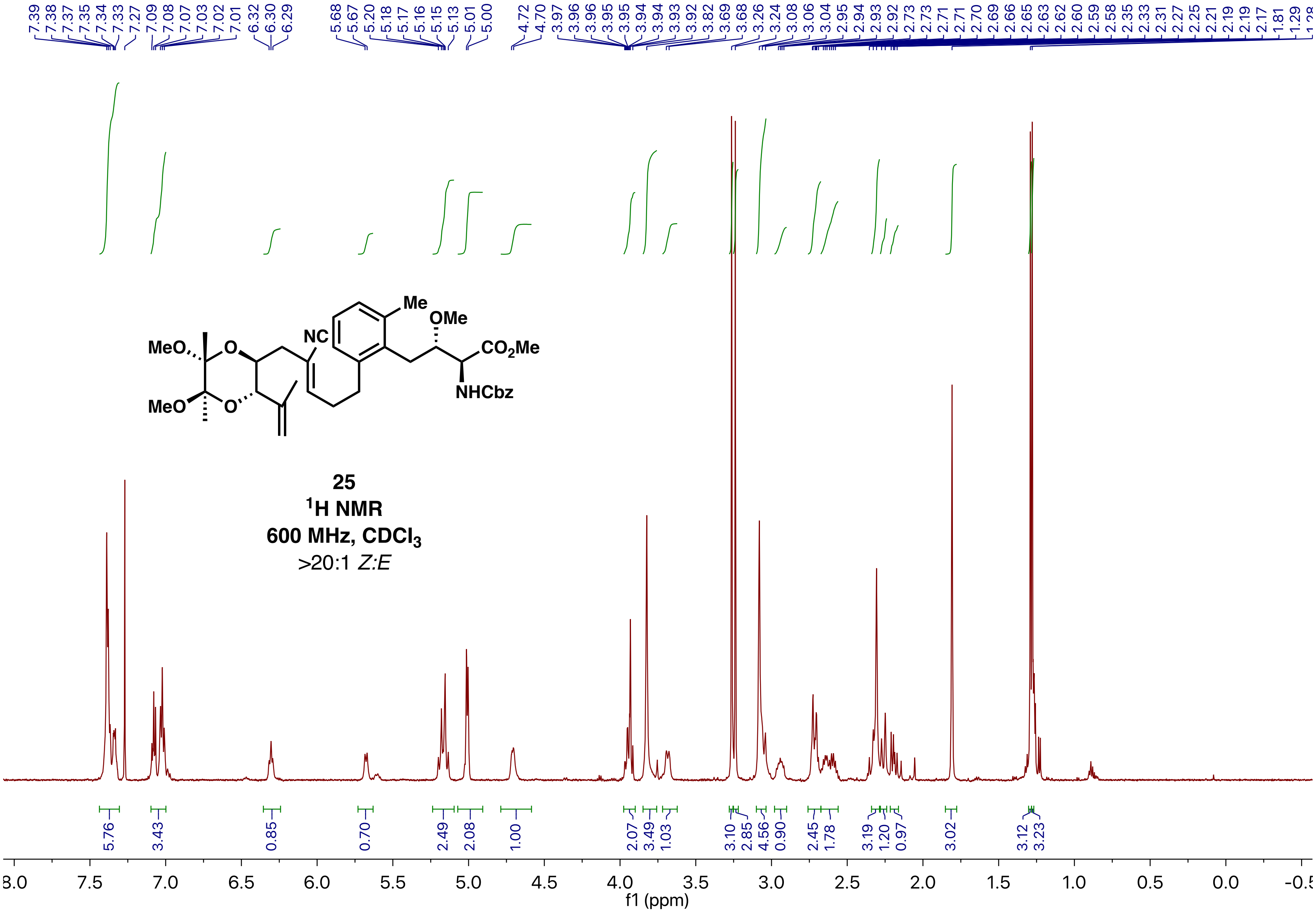

Me
OMe
MeO
O
NC
CO2Me
NHCbz
25
1H NMR
600 MHz, CDCl3
>20:1 Z:E
f1 (ppm)

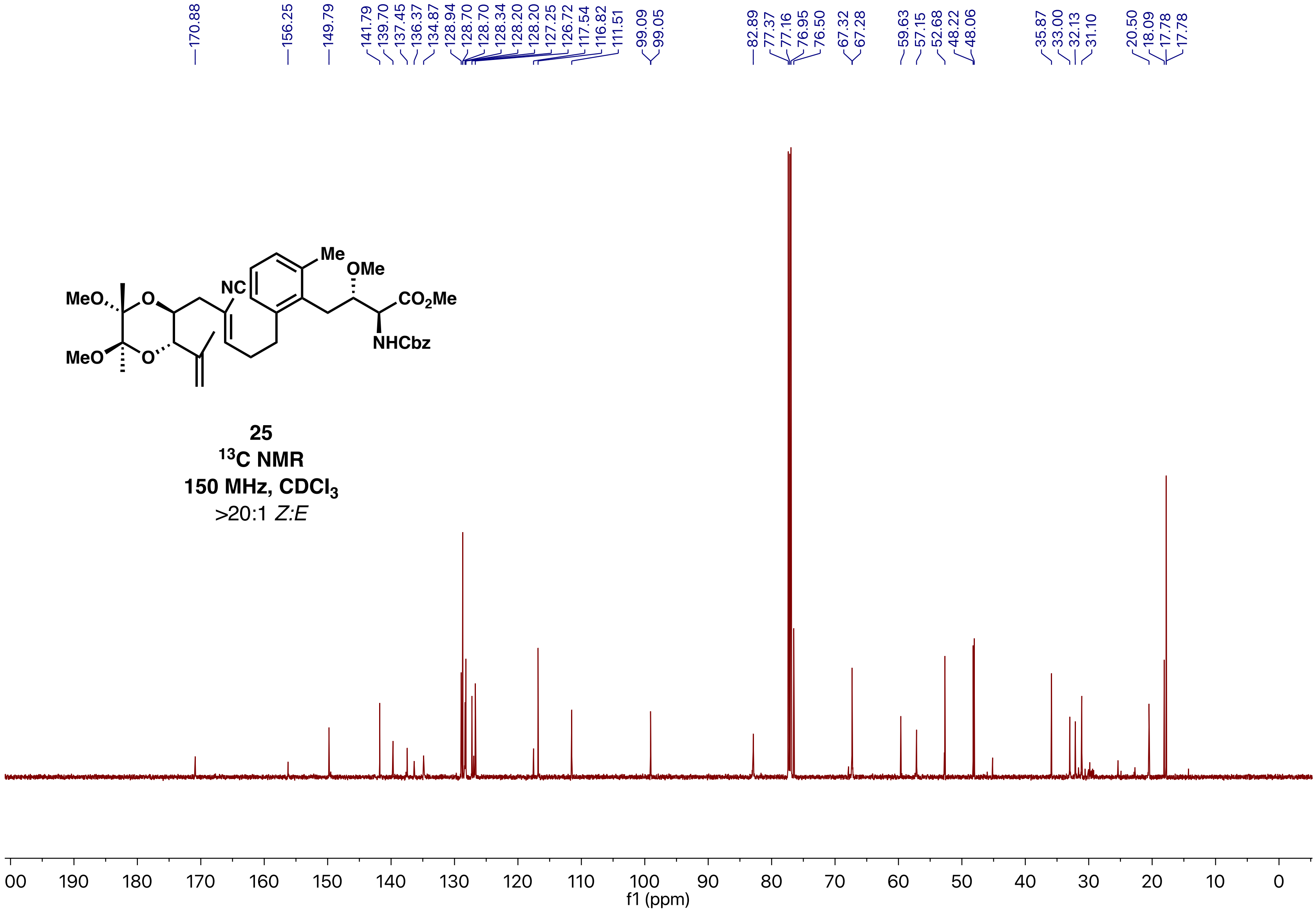

170.88
156.25
149.79
141.79
139.70
137.45
136.37
134.87
128.94
128.70
128.70
128.34
128.20
128.20
127.25
126.72
117.54
116.82
111.51
99.09
99.05
82.89
77.37
77.16
76.95
76.50
67.32
67.28
59.63
57.15
52.68
48.22
48.06
35.87
33.00
32.13
31.10
20.50
18.09
17.78
17.78
MeO
O
MeO
O
NC
Me
OMe
CO2Me
NHCbz
25
13C NMR
150 MHz, CDCl3
>20:1 Z:E
00
190
180
170
160
150
140
130
120
110
100
90
80
70
60
50
40
30
20
10
0
f1 (ppm)

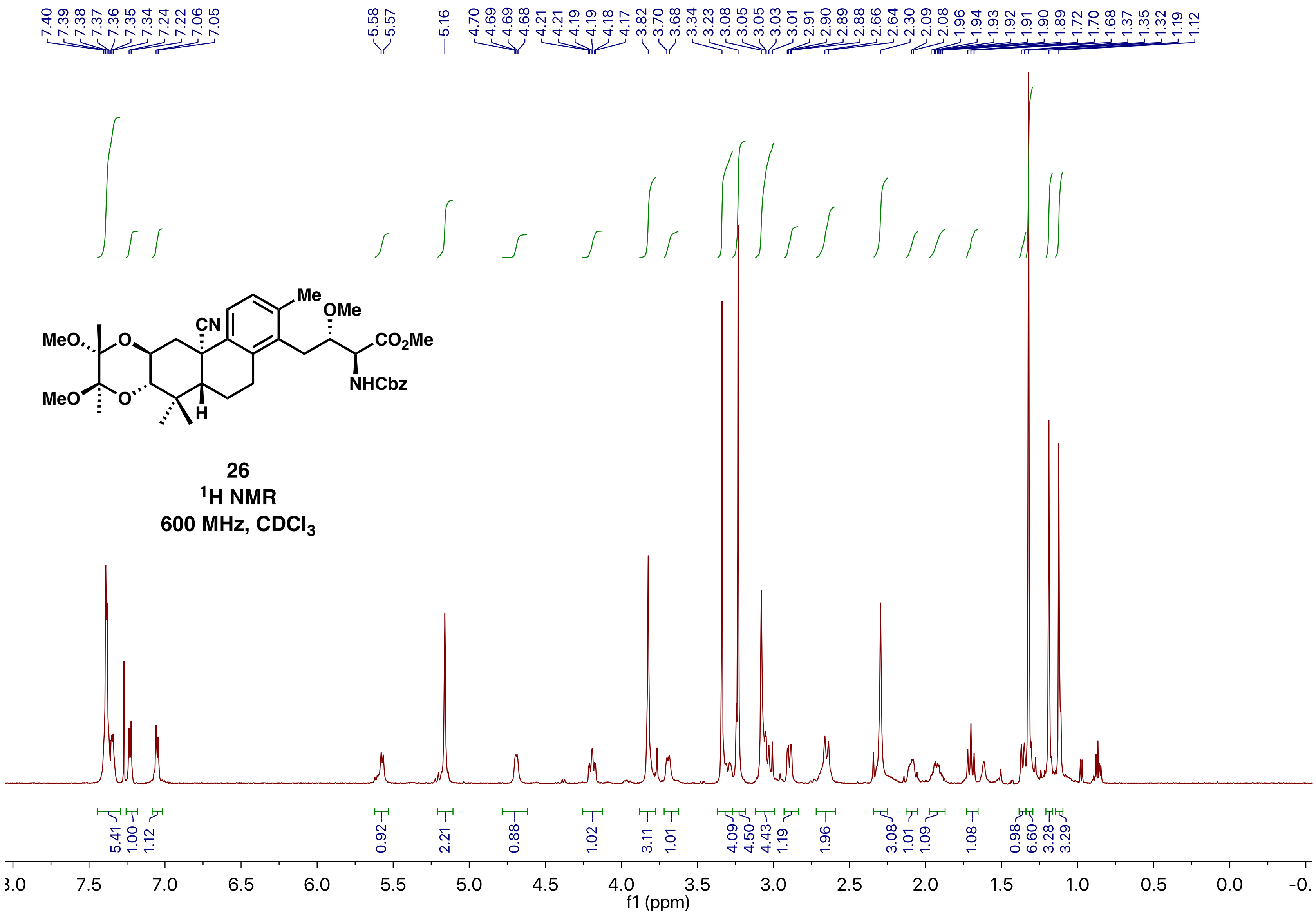

26
^{1}H NMR
600 MHz, $CDCl_3$

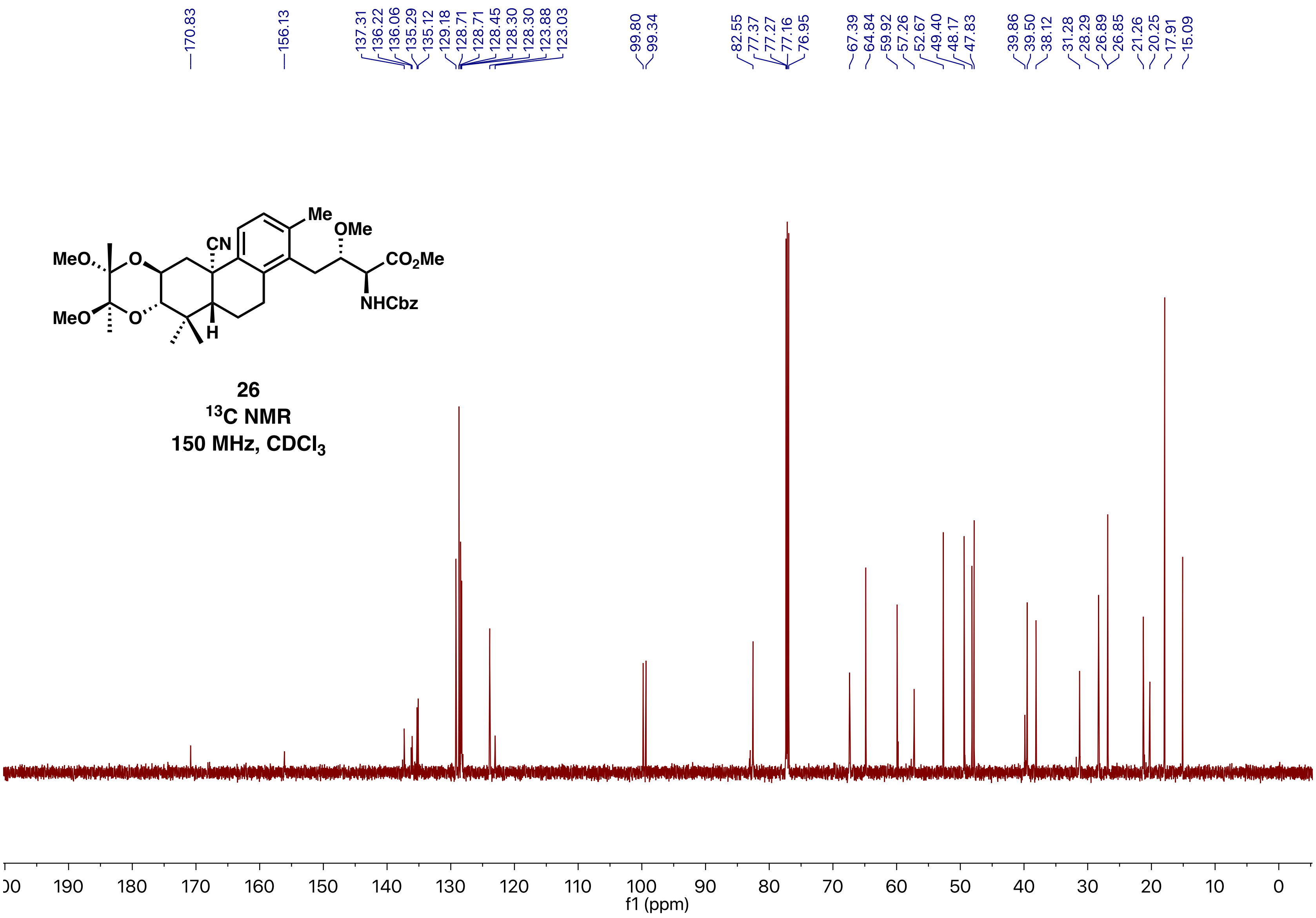

26
^{13}C NMR
150 MHz, $CDCl_3$

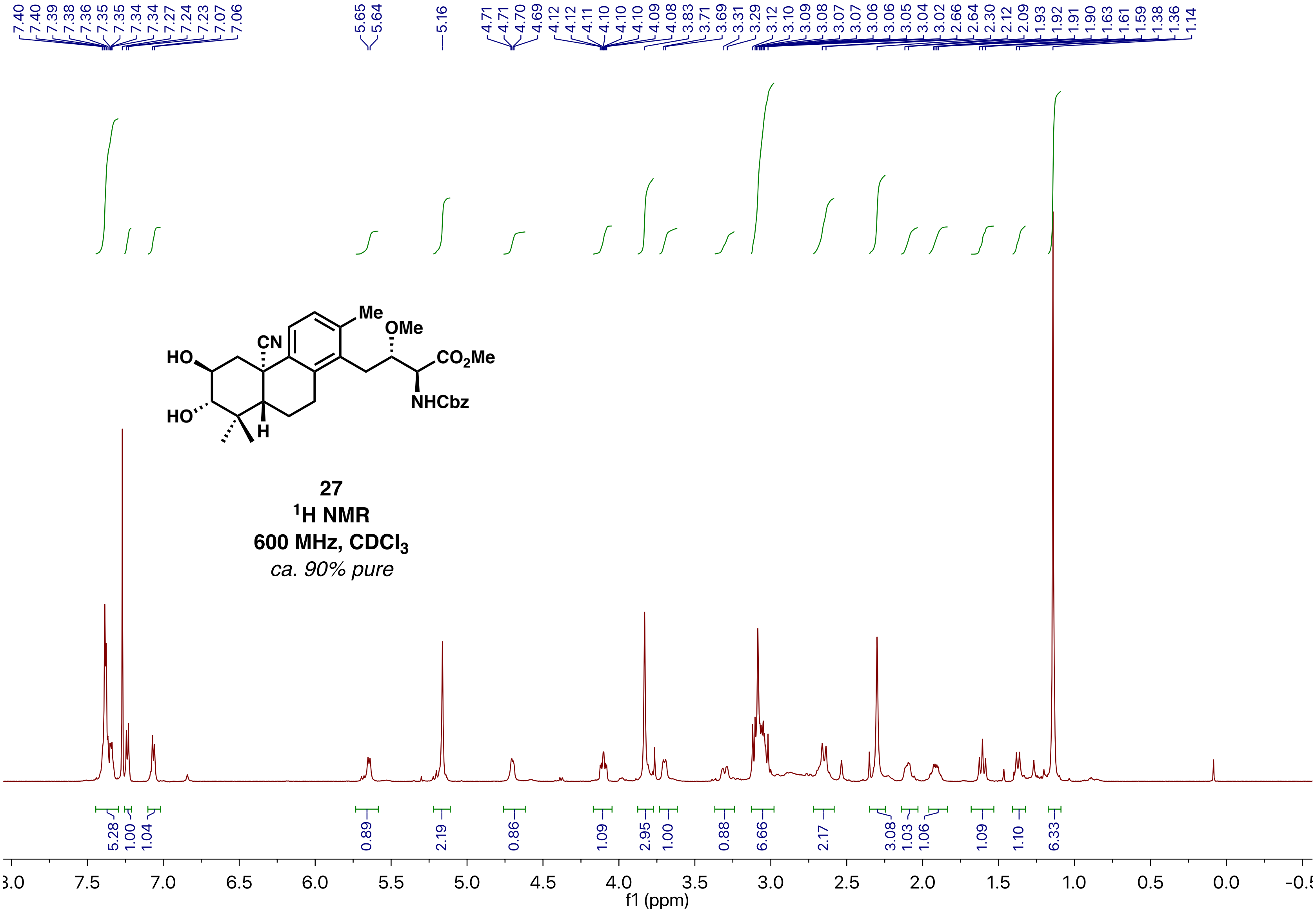

27

^{1}H NMR

600 MHz, $CDCl_3$

ca. 90% pure

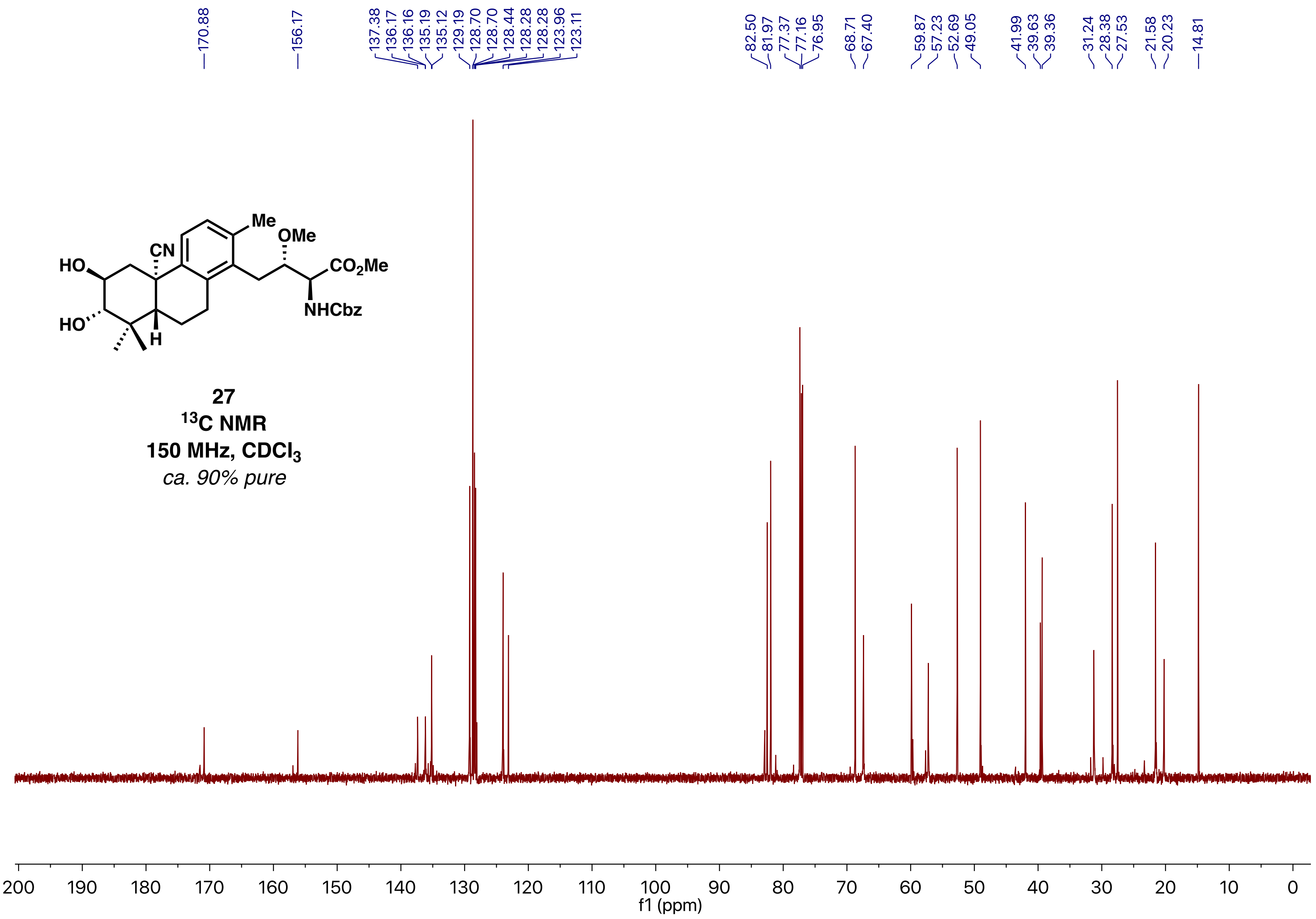
170.88
156.17
137.38
136.17
136.16
135.19
135.12
129.19
128.70
128.70
128.70
128.44
128.28
128.28
123.96
123.11
82.50
81.97
77.37
77.16
76.95
68.71
67.40
59.87
57.23
52.69
49.05
41.99
39.63
39.36
31.24
28.38
27.53
21.58
20.23
14.81
Me
OMe
CN
HO
CO2Me
HO
NHCbz
H
27
13C NMR
150 MHz, CDCl3
ca. 90% pure
200 190 180 170 160 150 140 130 120 110 100 90 80 70 60 50 40 30 20 10 0
f1 (ppm)

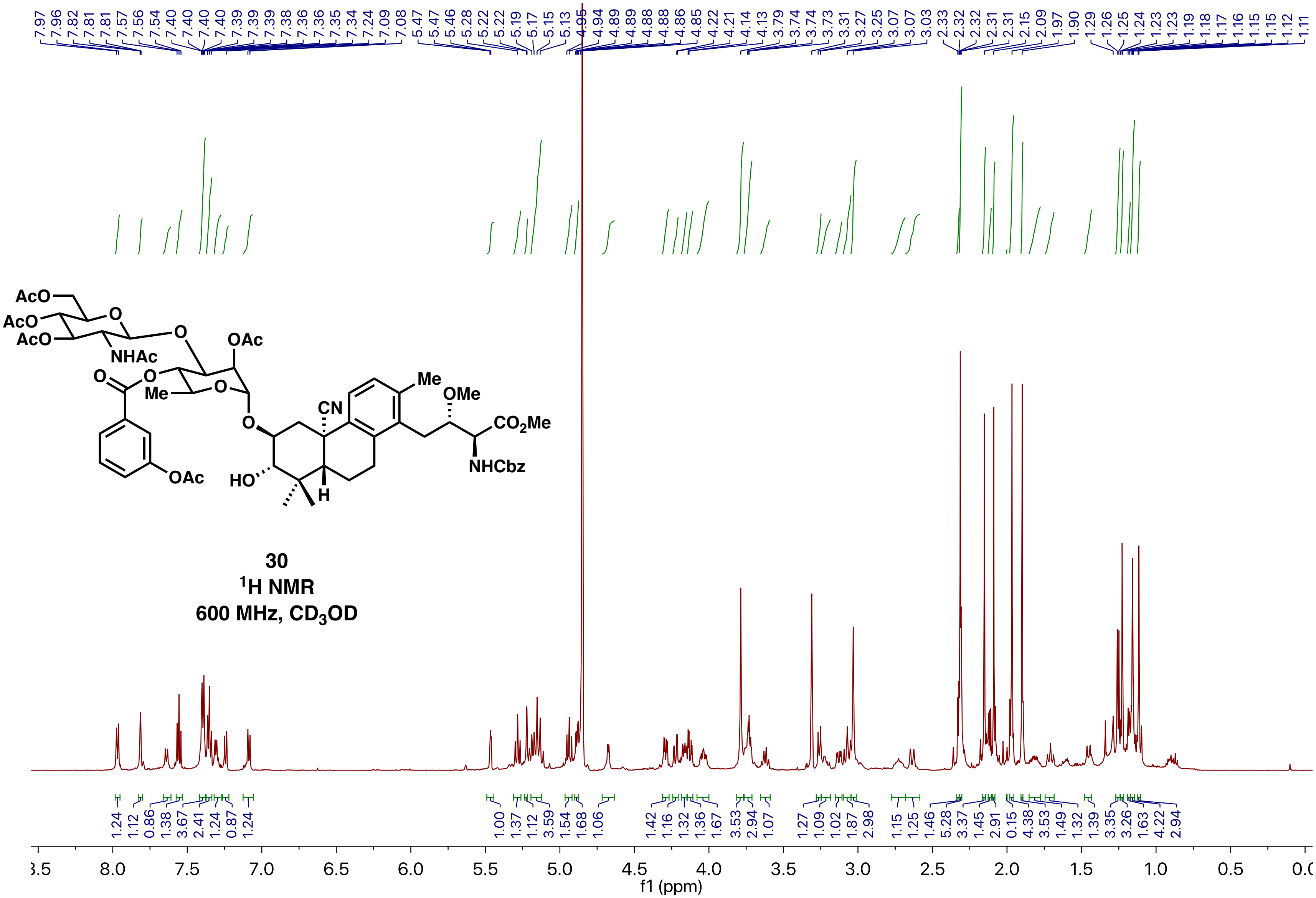

30
^{1}H NMR
600 MHz, CD_3OD

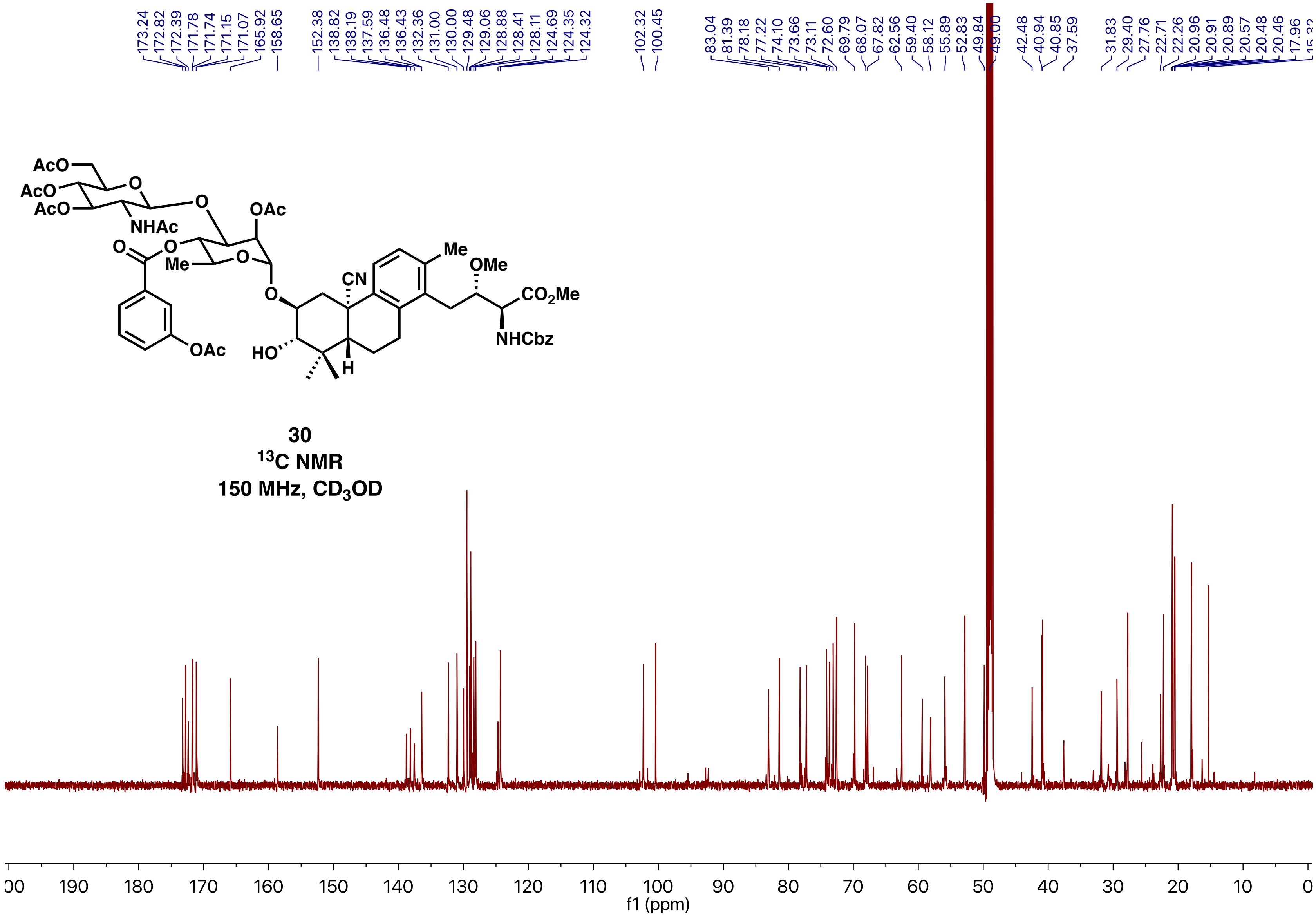

173.24
172.82
172.39
171.78
171.74
171.15
171.07
165.92
158.65
152.38
138.82
138.19
137.59
136.48
136.43
132.36
131.00
130.00
129.48
129.06
128.88
128.41
128.11
124.69
124.35
124.32
102.32
100.45
83.04
81.39
78.18
77.22
74.10
73.66
73.11
72.60
69.79
68.07
67.82
62.56
59.40
58.12
55.89
52.83
49.84
49.00
42.48
40.94
40.85
37.59
31.83
29.40
27.76
22.71
22.26
20.96
20.91
20.89
20.57
20.48
20.46
17.96
AcO
AcO
AcO
O
O
NHAc
OAc
O
O
Me
O
OAc
CN
Me
OMe
CO2Me
NHCbz
HO
H
OAc
30
13C NMR
150 MHz, CD3OD
f1 (ppm)

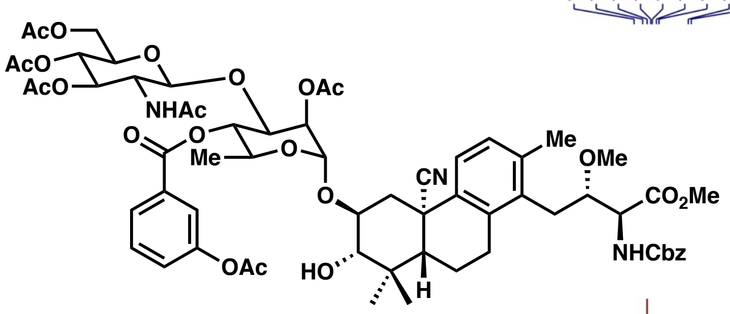

30
DEPT90
150 MHz, CD_3OD

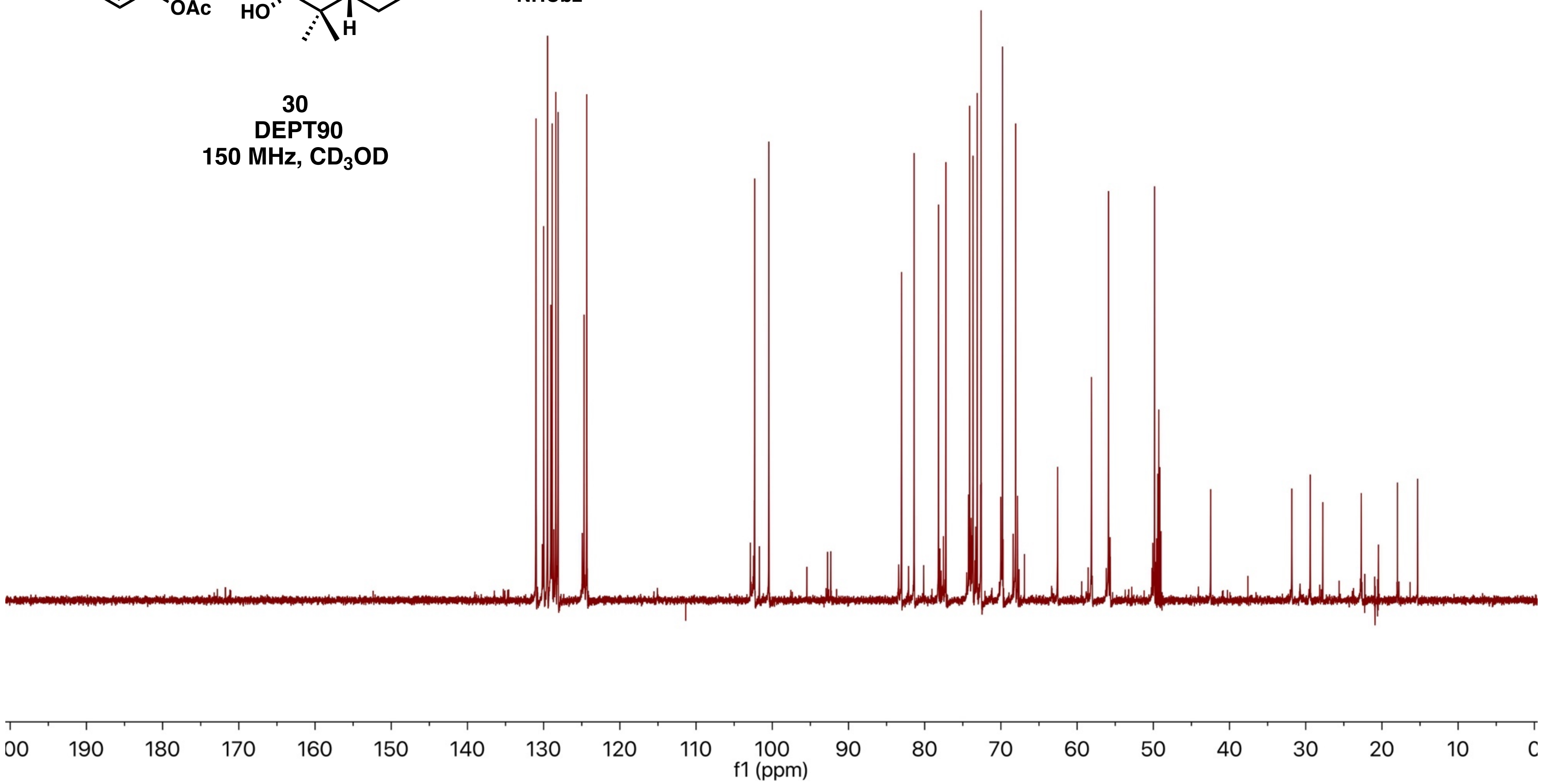

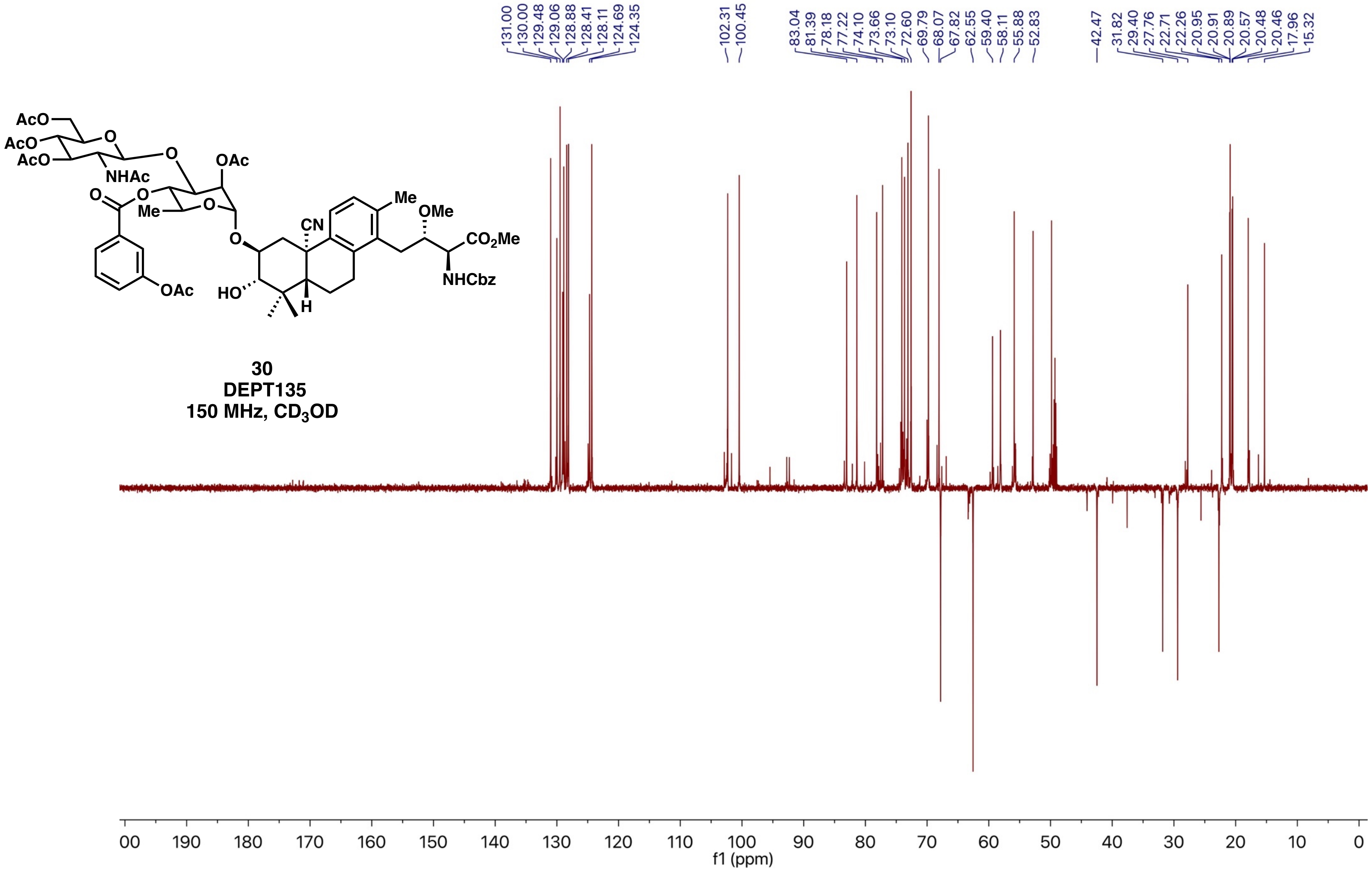

30
DEPT135
150 MHz, CD_3OD

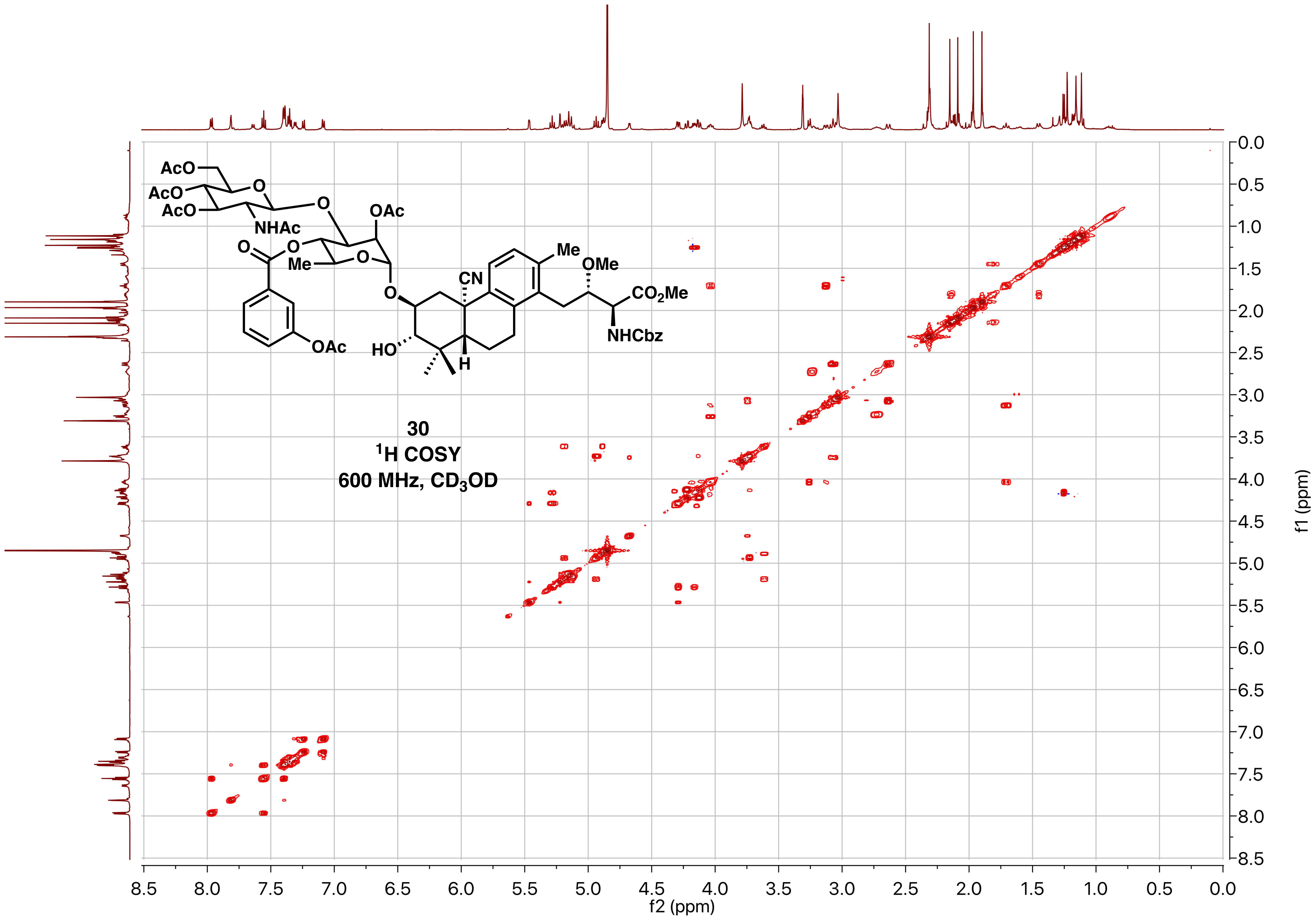
30
1H COSY
600 MHz, CD3OD
f2 (ppm)
f1 (ppm)

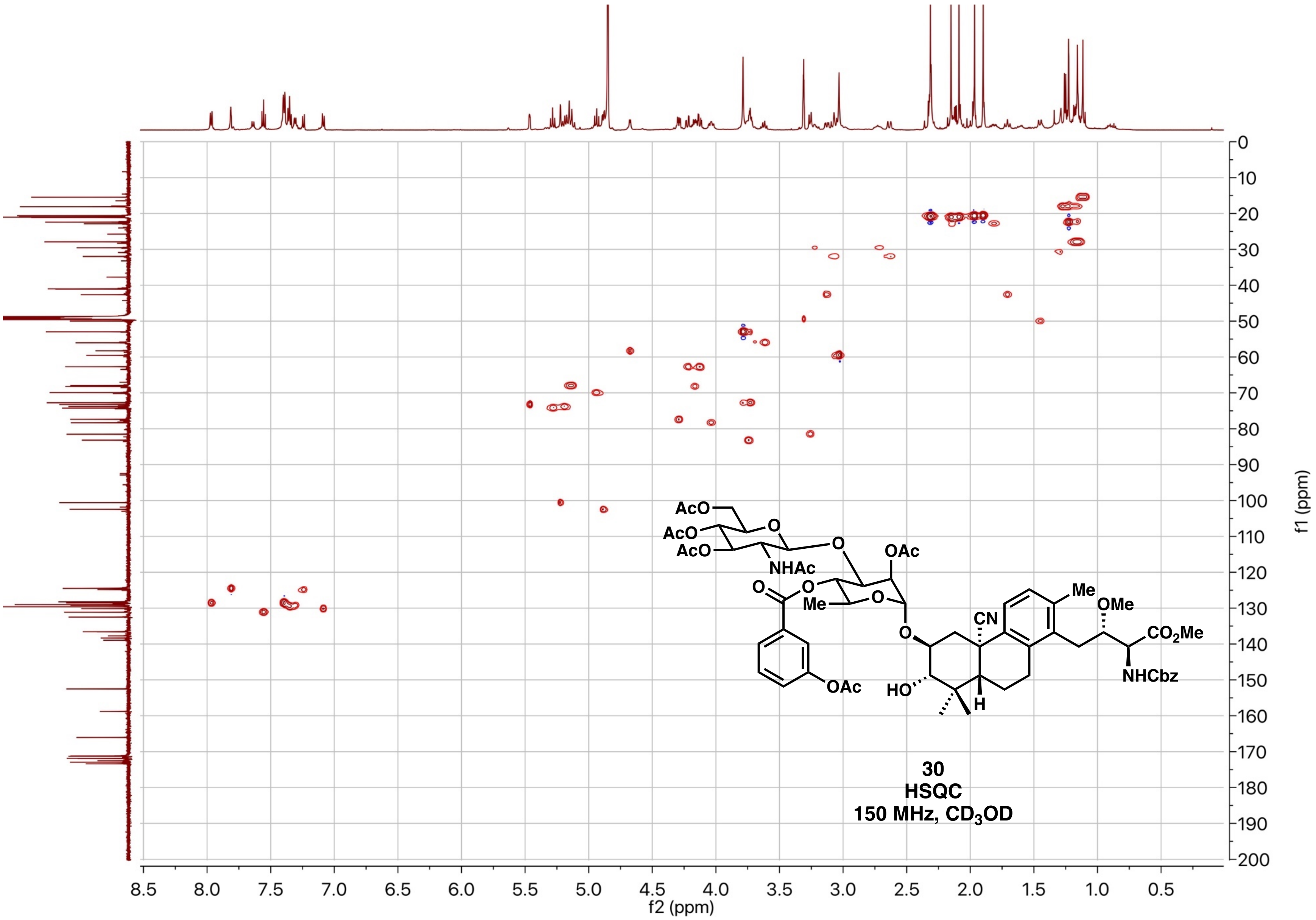
AcO
AcO
AcO
O
O
NHAc
OAc
O
O
Me
O
O
OAc
CN
Me
OMe
CO2Me
NHCbz
HO
H
30
HSQC
150 MHz, CD3OD
f2 (ppm)
f1 (ppm)

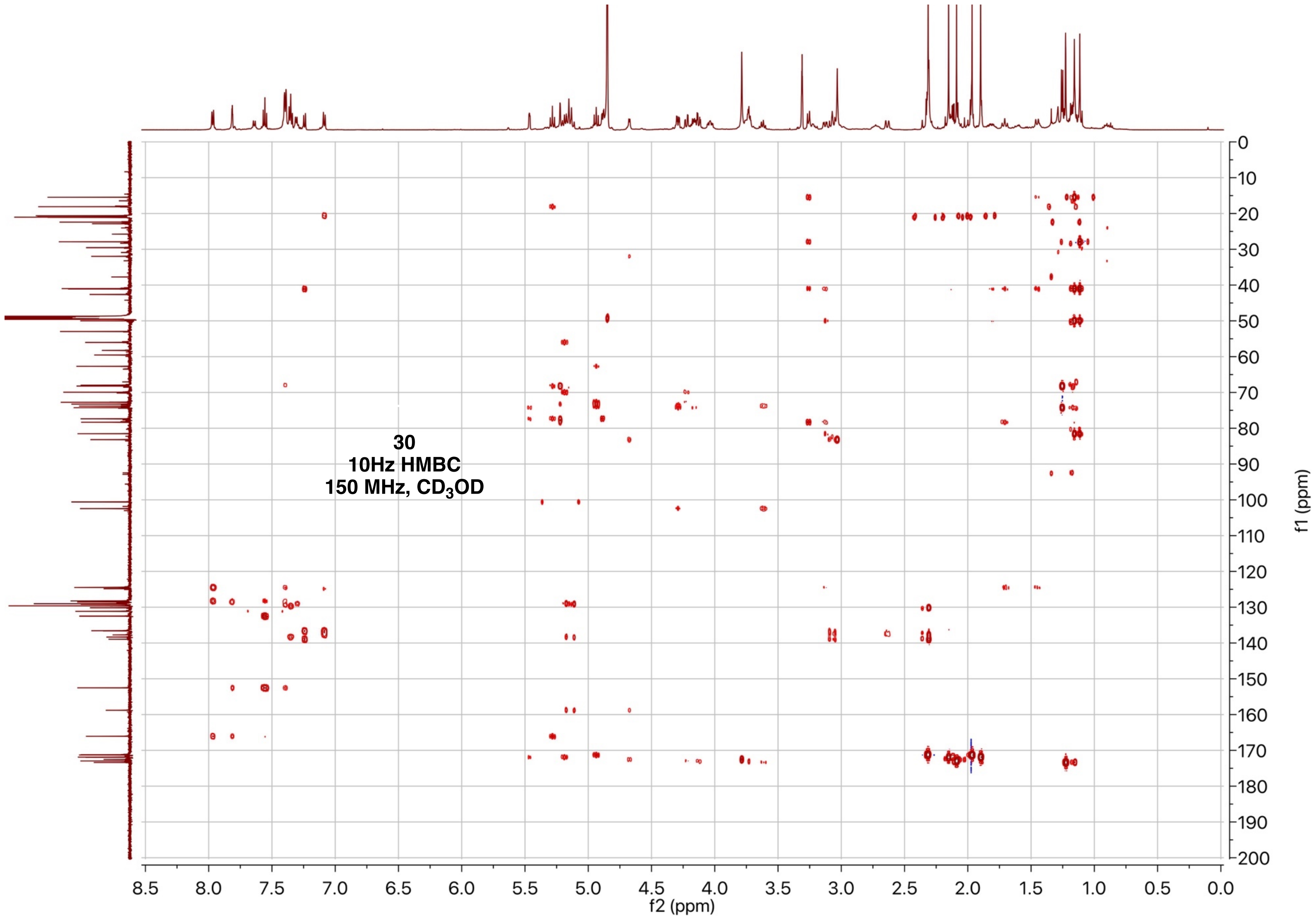
30
10Hz HMBC
150 MHz, CD3OD
f2 (ppm)
f1 (ppm)

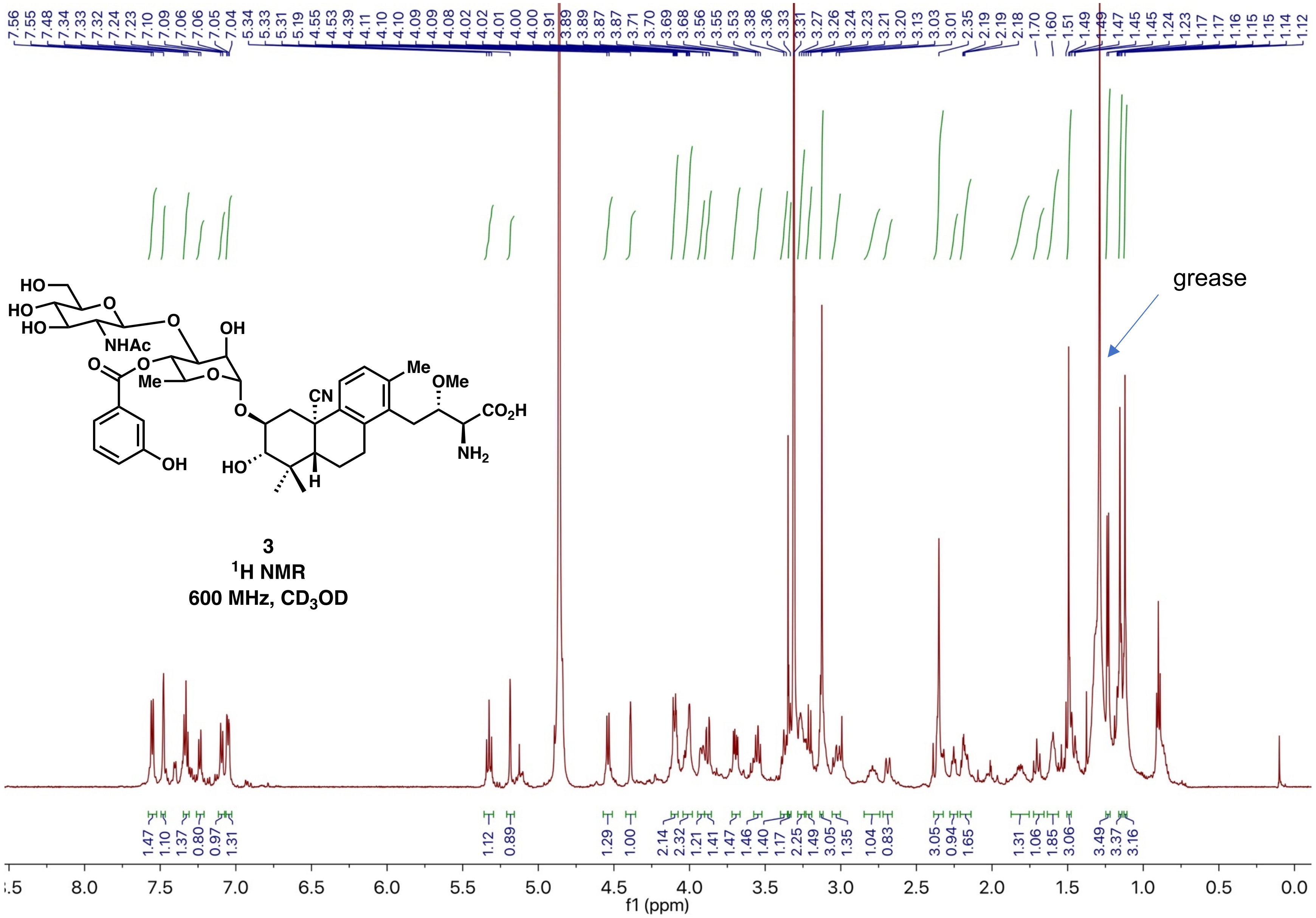
3
1H NMR
600 MHz, CD3OD
grease
f1 (ppm)

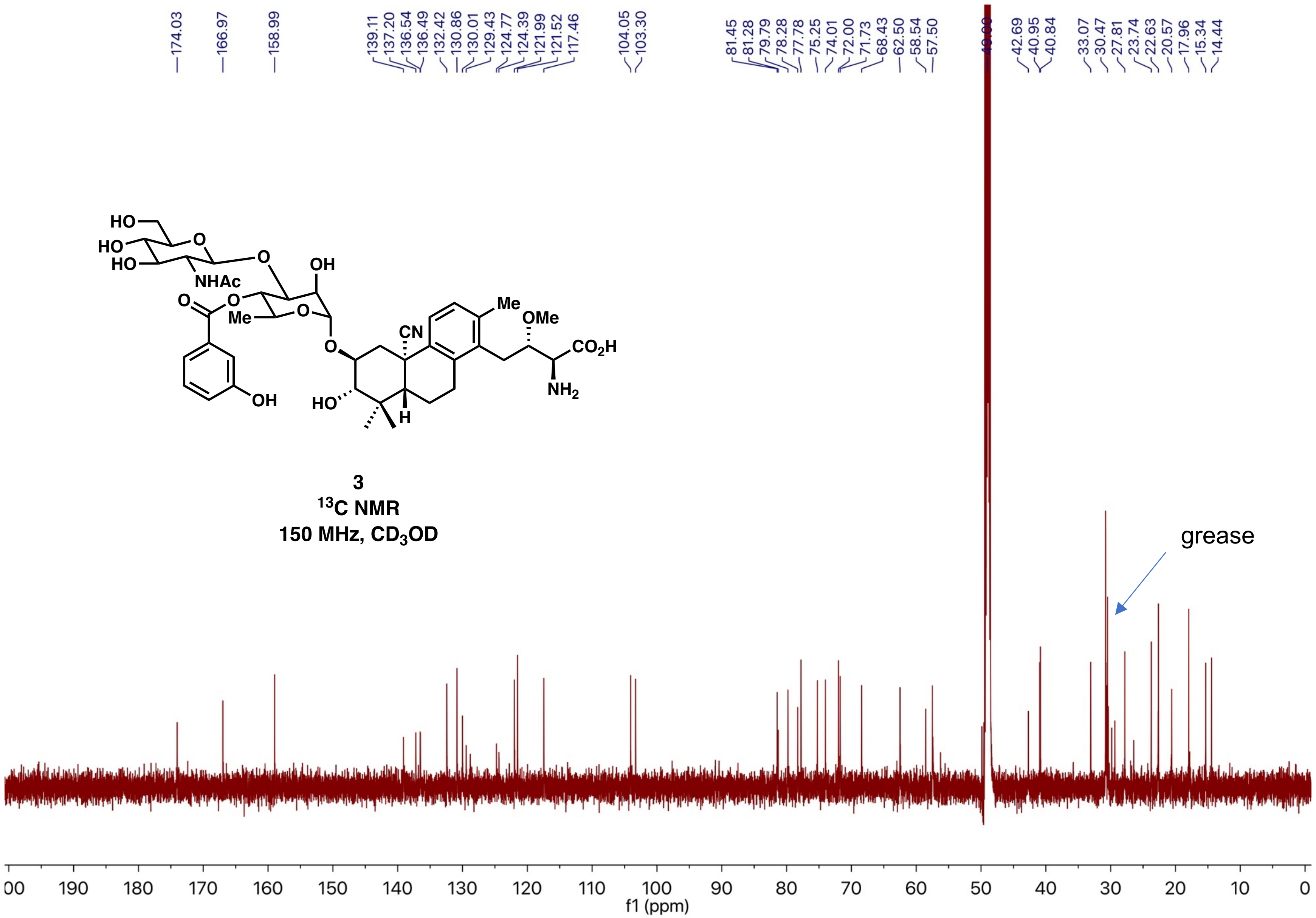

3
^{13}C NMR
150 MHz, CD_3OD
